# Supplementary figures and images for: SETD2 methyltransferase activity promotes correct transcription initiation and termination
Source: EMBO Rep. 2026 Mar 23;27(9):2218–42. doi: 10.1038/s44319-026-00744-1 (PMC13172057; doi:10.1038/s44319-026-00744-1)

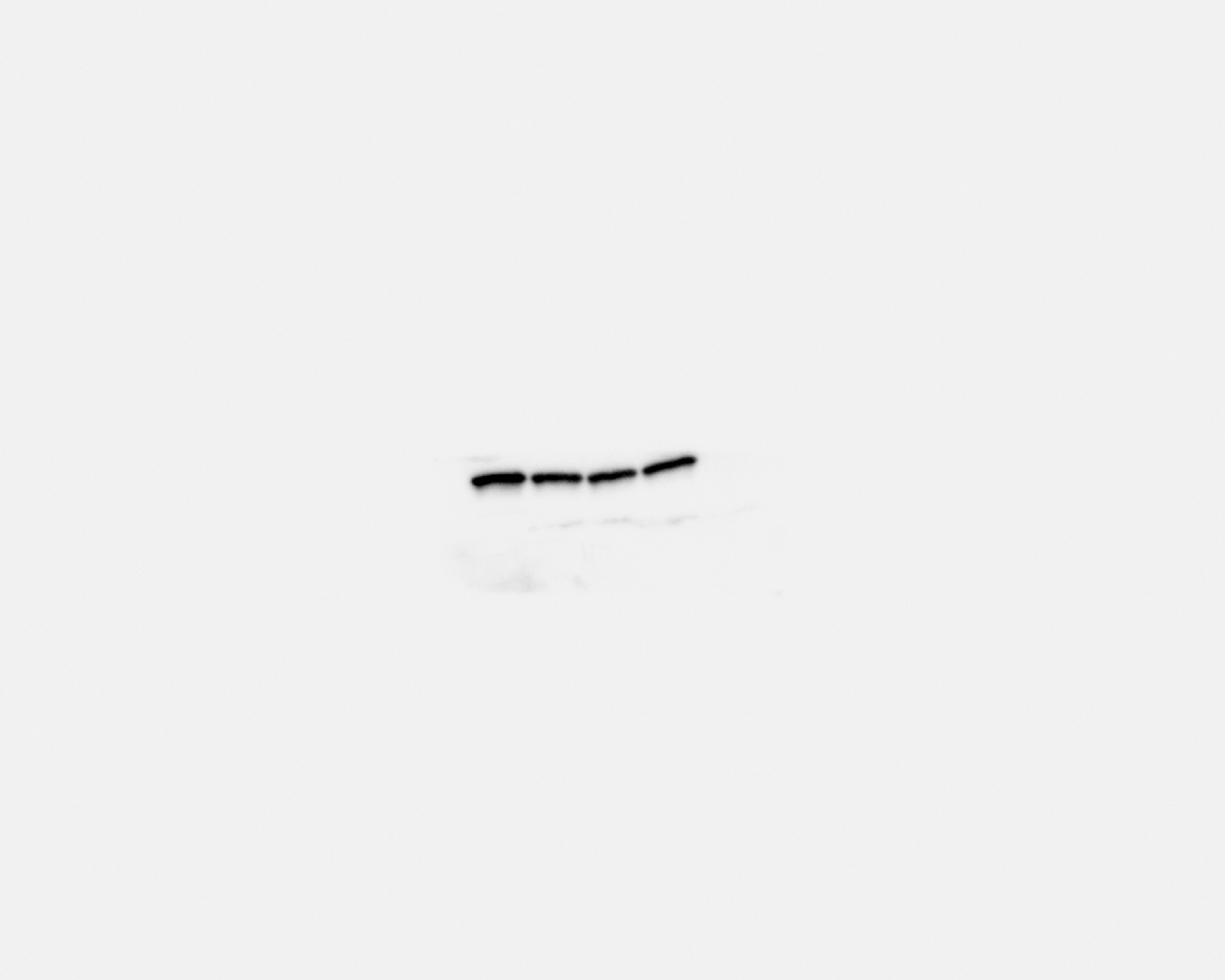

Supplement: Supplementary file 5 — Source data Fig. 1 [file 44319_2026_744_MOESM5_ESM.zip › Figure EV4/EV4A HEK293 + EPZ-719/2025-10-23 m hek293 h3 nuc(Chemiluminescence).jpg]

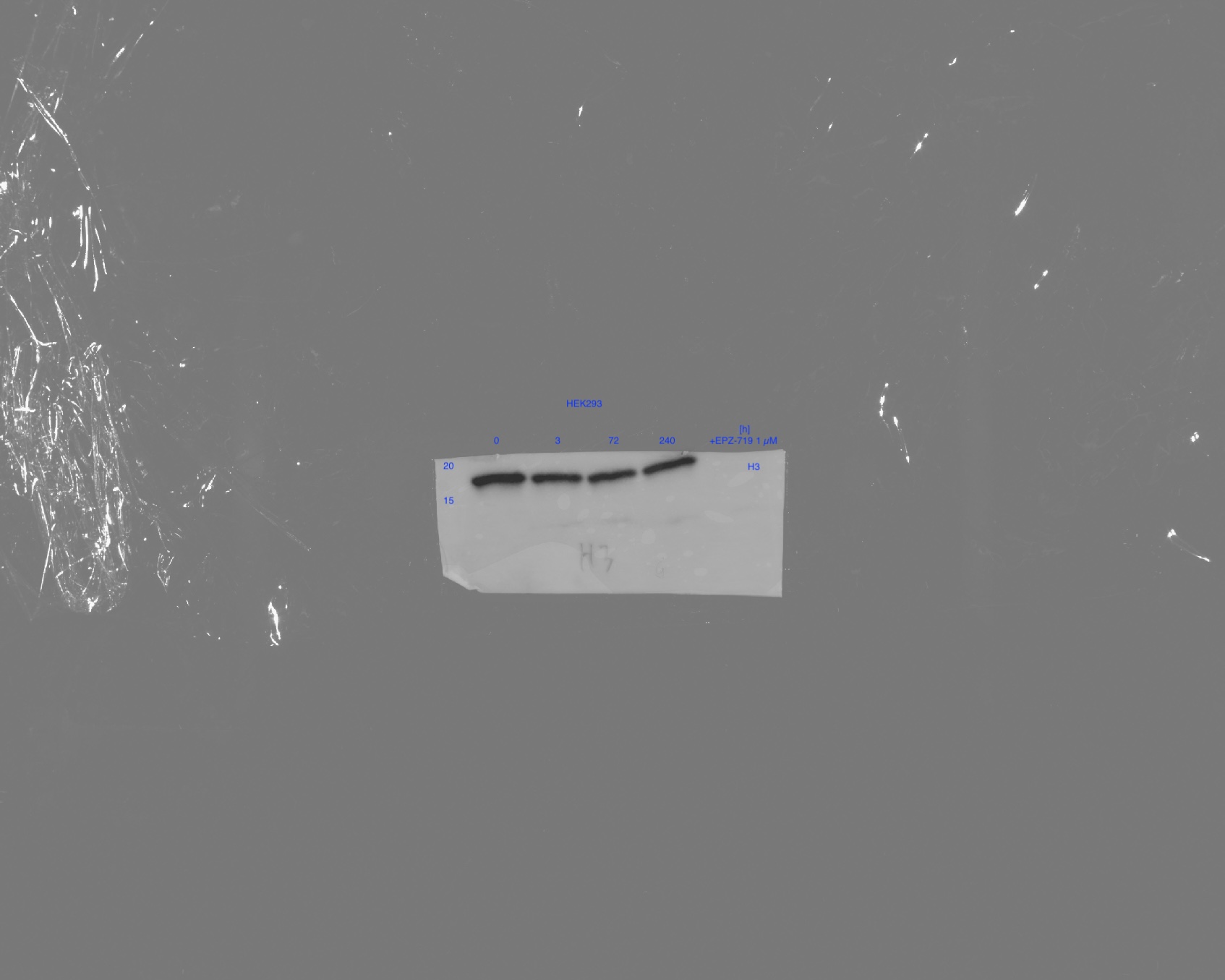

Supplement: Supplementary file 5 — Source data Fig. 1 [file 44319_2026_744_MOESM5_ESM.zip › Figure EV4/EV4A HEK293 + EPZ-719/2025-10-23 m hek293 h3 nuc(Composite).jpg]

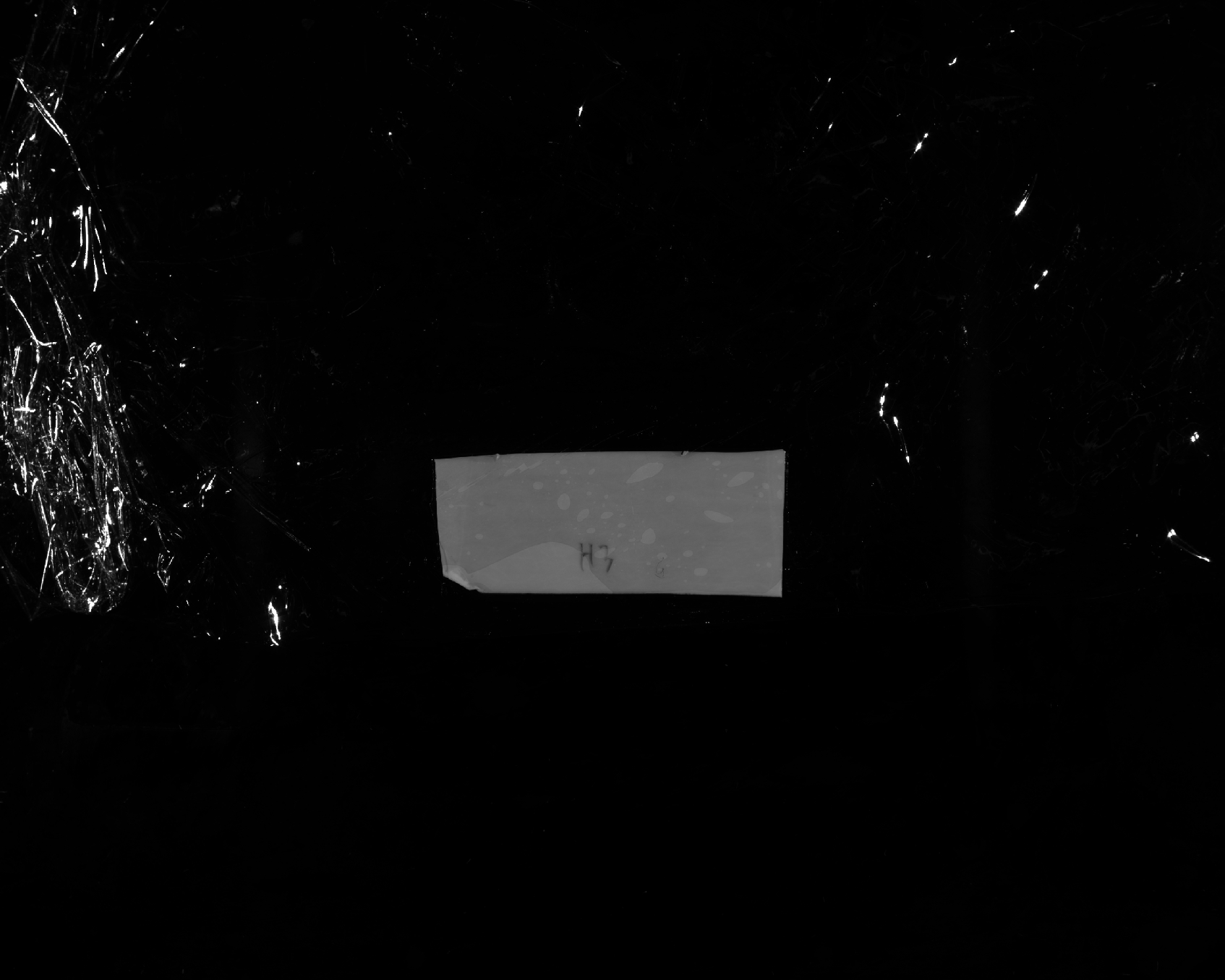

Supplement: Supplementary file 5 — Source data Fig. 1 [file 44319_2026_744_MOESM5_ESM.zip › Figure EV4/EV4A HEK293 + EPZ-719/2025-10-23 m hek293 h3 nuc(Ponceau S).jpg]

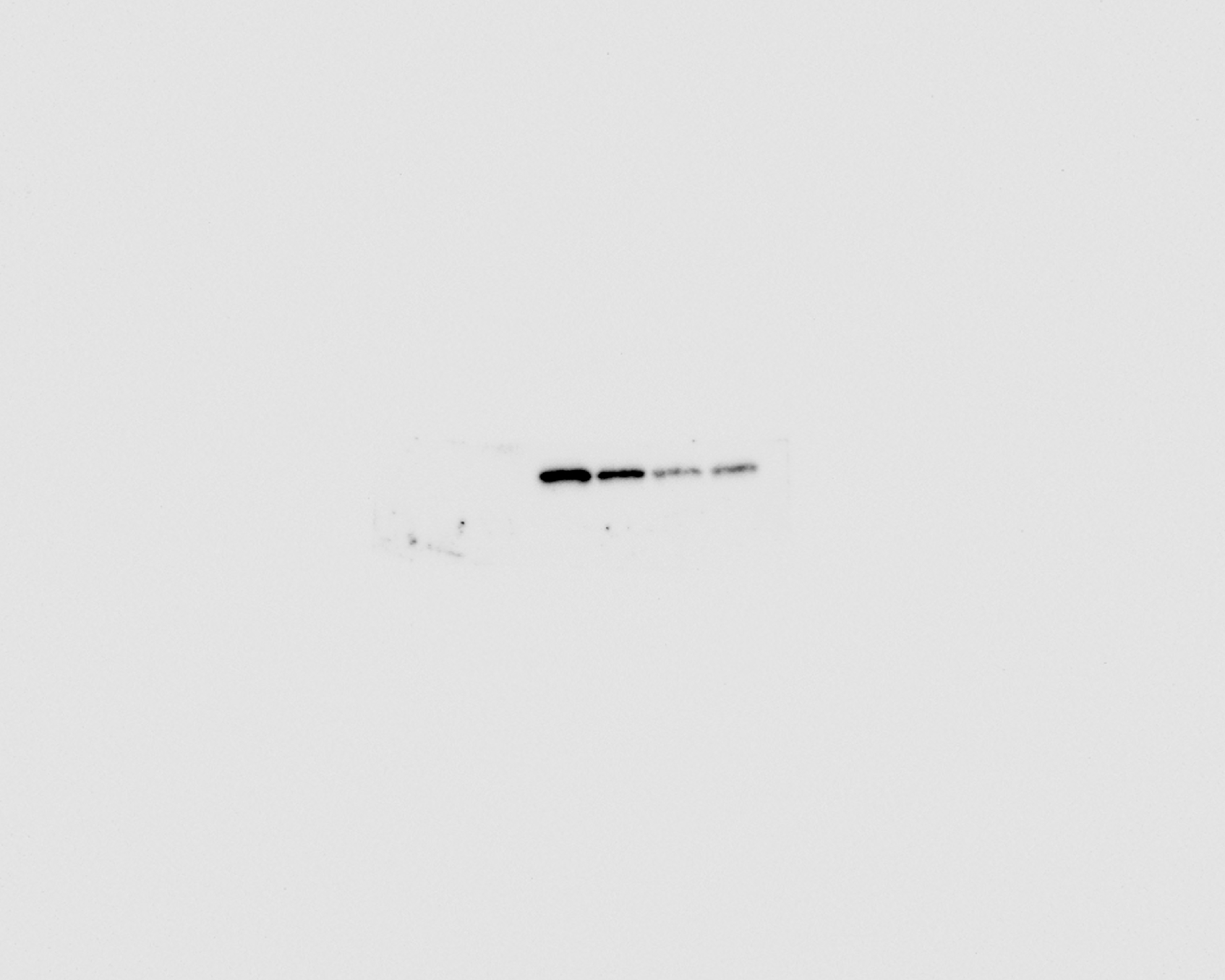

Supplement: Supplementary file 5 — Source data Fig. 1 [file 44319_2026_744_MOESM5_ESM.zip › Figure EV4/EV4A HEK293 + EPZ-719/2025-10-23 m hek293 h3k36me3 nuc(Chemiluminescence).jpg]

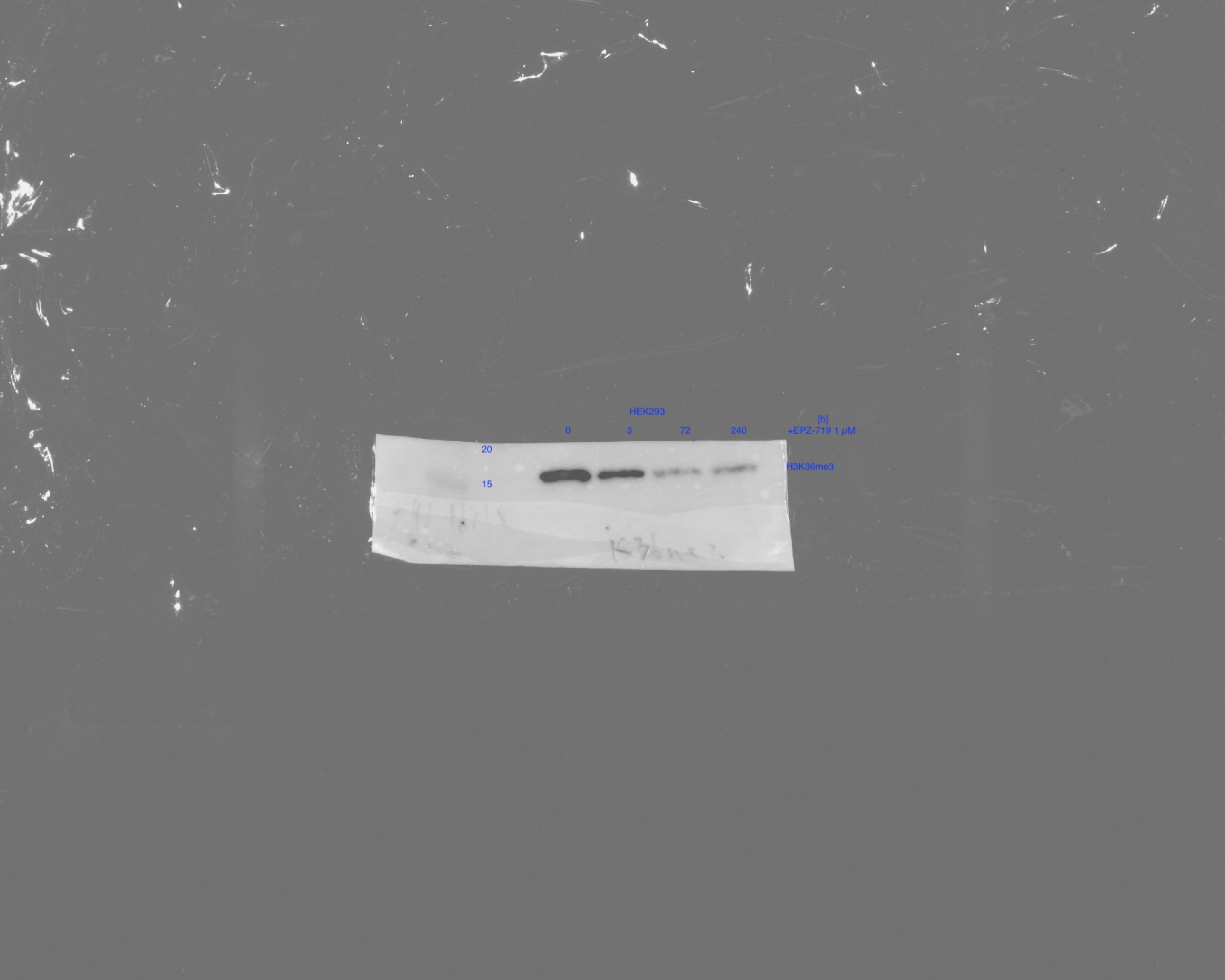

Supplement: Supplementary file 5 — Source data Fig. 1 [file 44319_2026_744_MOESM5_ESM.zip › Figure EV4/EV4A HEK293 + EPZ-719/2025-10-23 m hek293 h3k36me3 nuc(Composite).jpg]

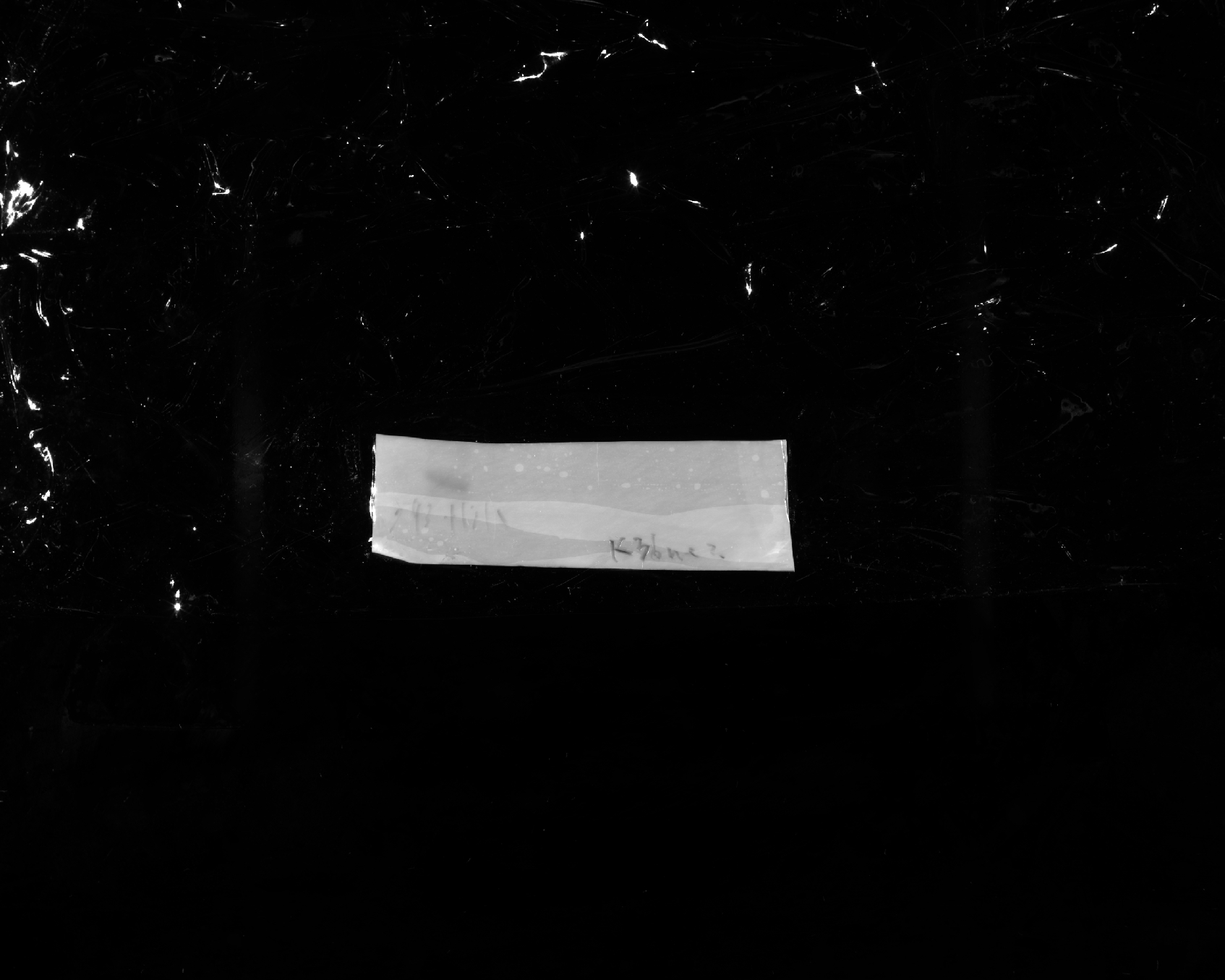

Supplement: Supplementary file 5 — Source data Fig. 1 [file 44319_2026_744_MOESM5_ESM.zip › Figure EV4/EV4A HEK293 + EPZ-719/2025-10-23 m hek293 h3k36me3 nuc(Ponceau S).jpg]

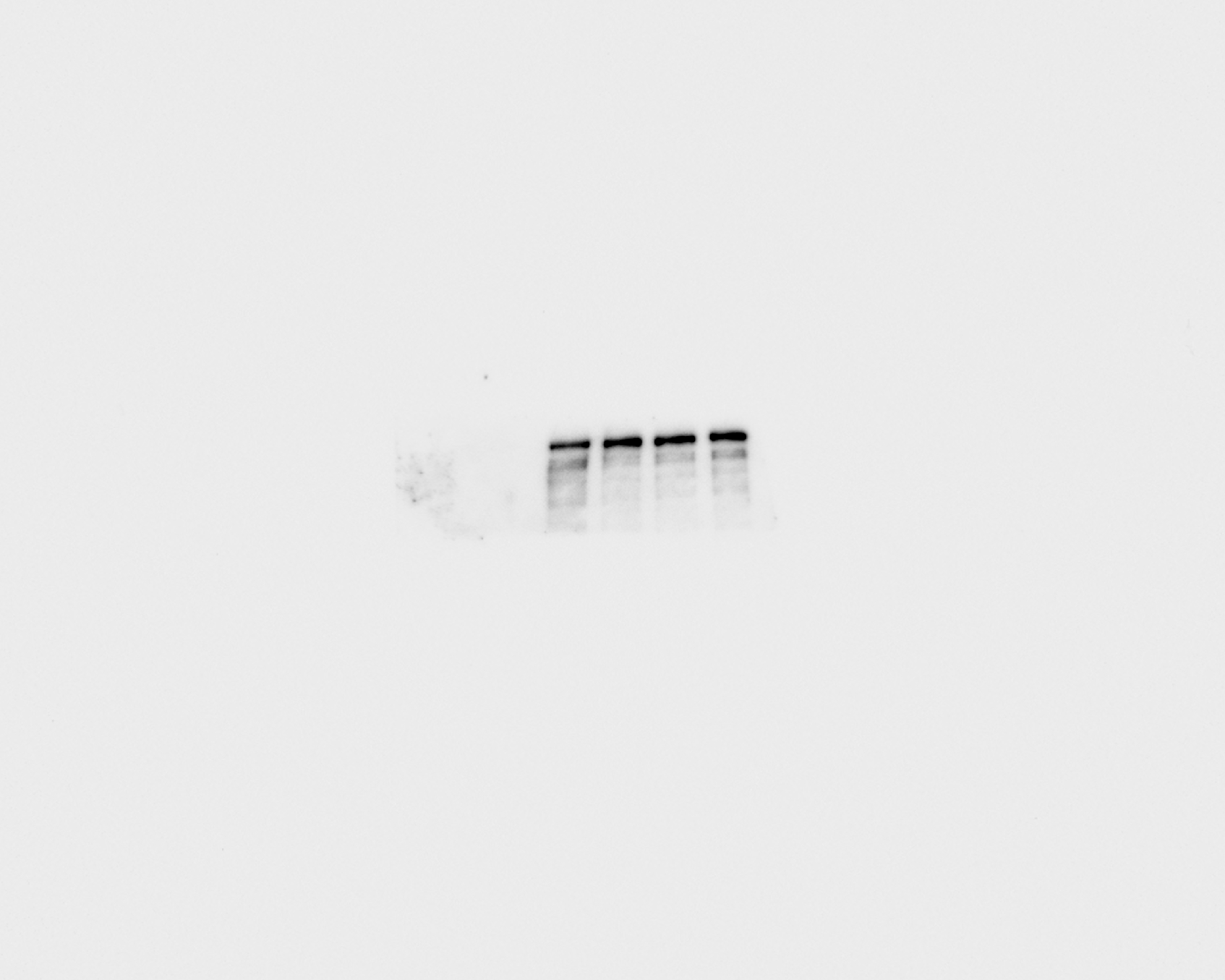

Supplement: Supplementary file 5 — Source data Fig. 1 [file 44319_2026_744_MOESM5_ESM.zip › Figure EV4/EV4A HEK293 + EPZ-719/2025-10-23 m hek293 setd2 nuc(Chemiluminescence).jpg]

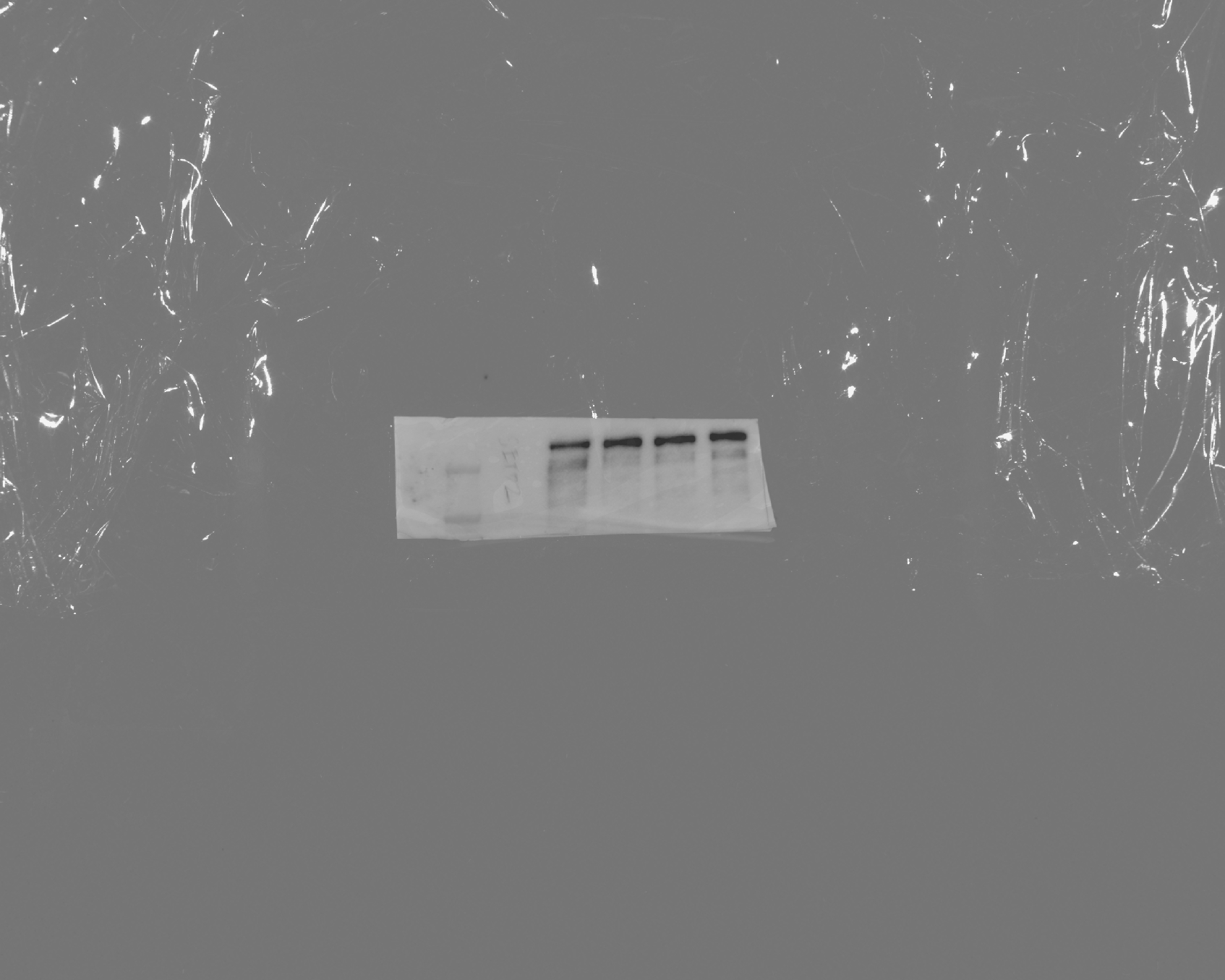

Supplement: Supplementary file 5 — Source data Fig. 1 [file 44319_2026_744_MOESM5_ESM.zip › Figure EV4/EV4A HEK293 + EPZ-719/2025-10-23 m hek293 setd2 nuc(Composite).jpg]

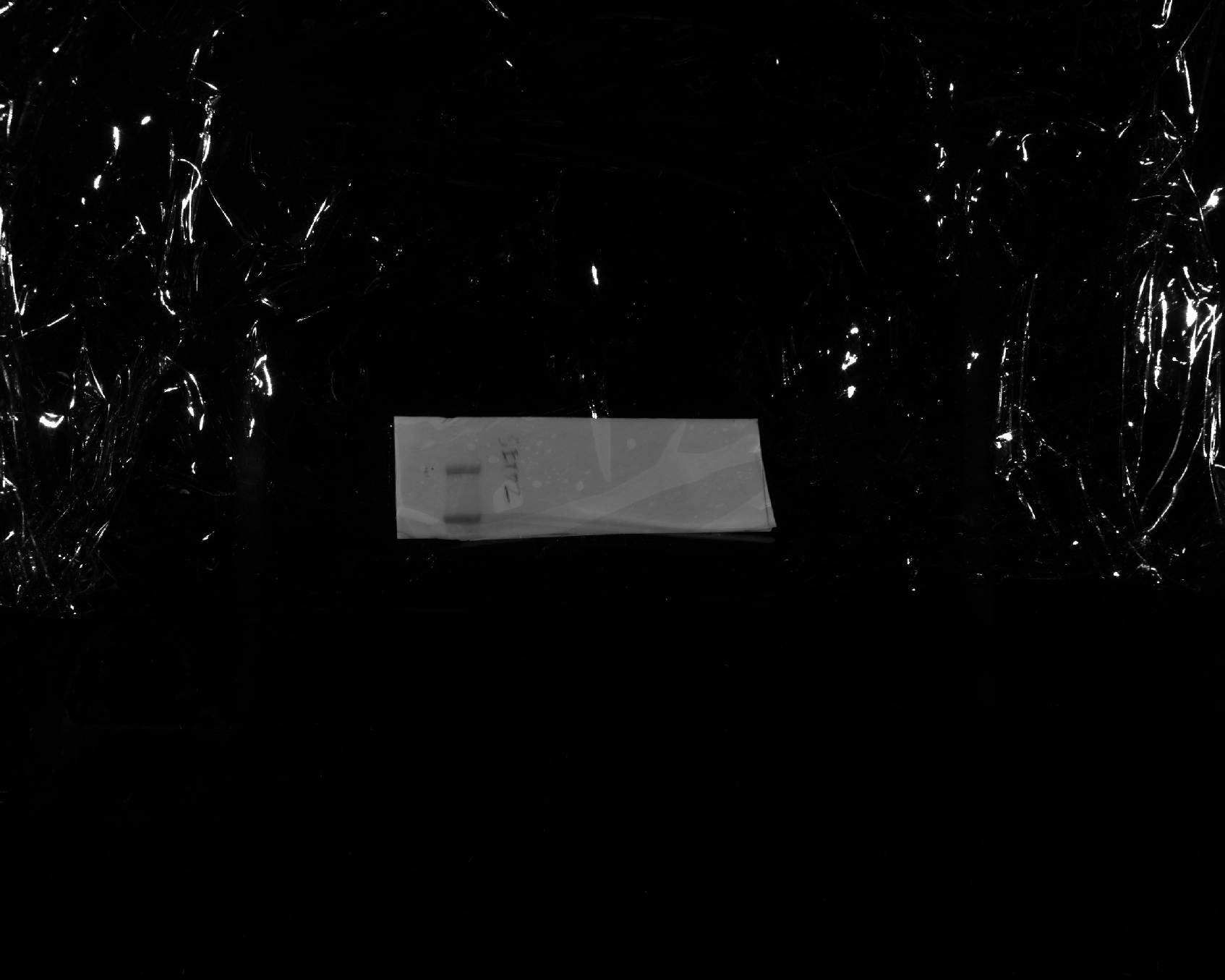

Supplement: Supplementary file 5 — Source data Fig. 1 [file 44319_2026_744_MOESM5_ESM.zip › Figure EV4/EV4A HEK293 + EPZ-719/2025-10-23 m hek293 setd2 nuc(Ponceau S).jpg]

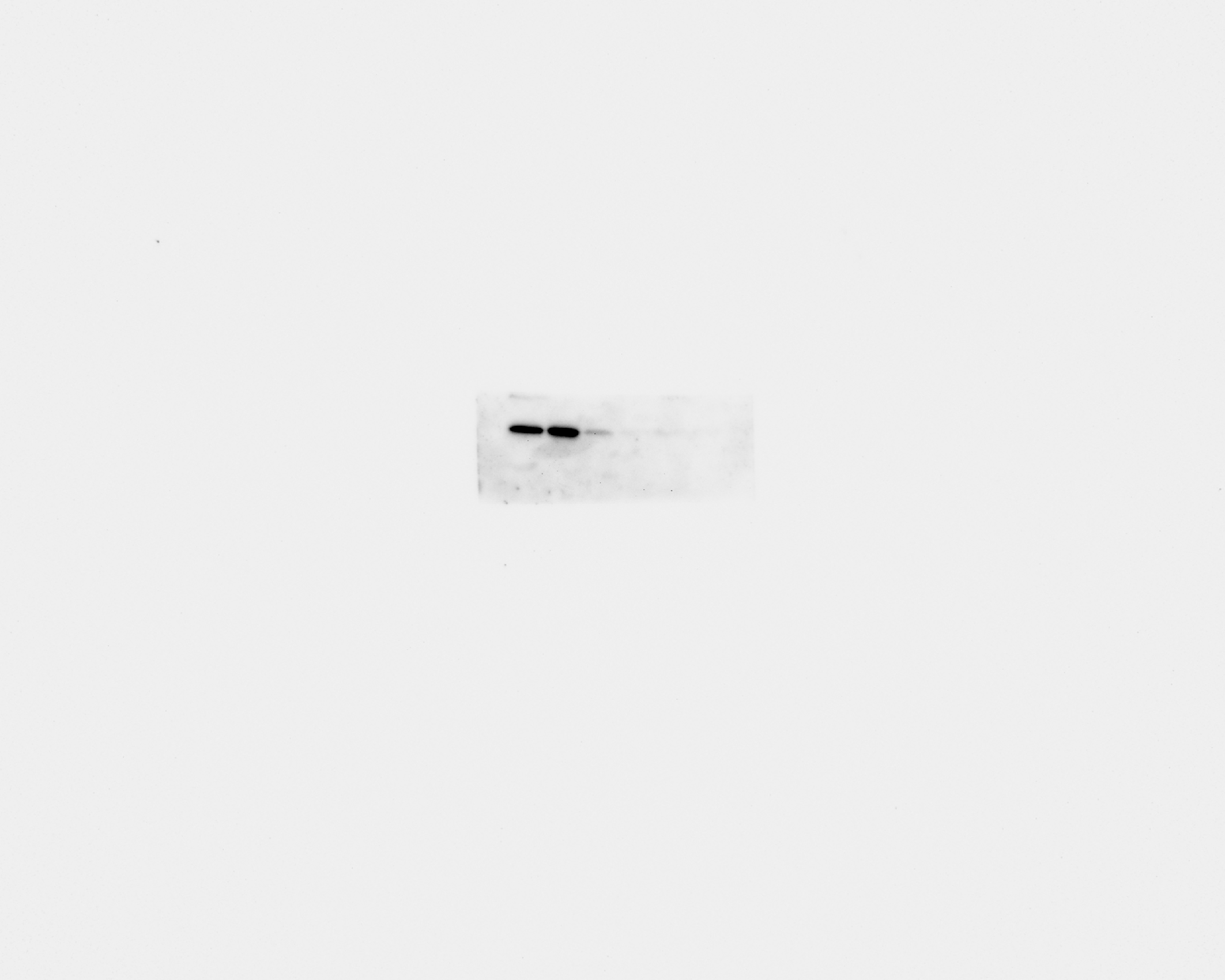

Supplement: Supplementary file 5 — Source data Fig. 1 [file 44319_2026_744_MOESM5_ESM.zip › Figure EV4/EV4A hTERT-RPE + EPZ-719/2025-11-21 rpe h3k36m3(Chemiluminescence).jpg]

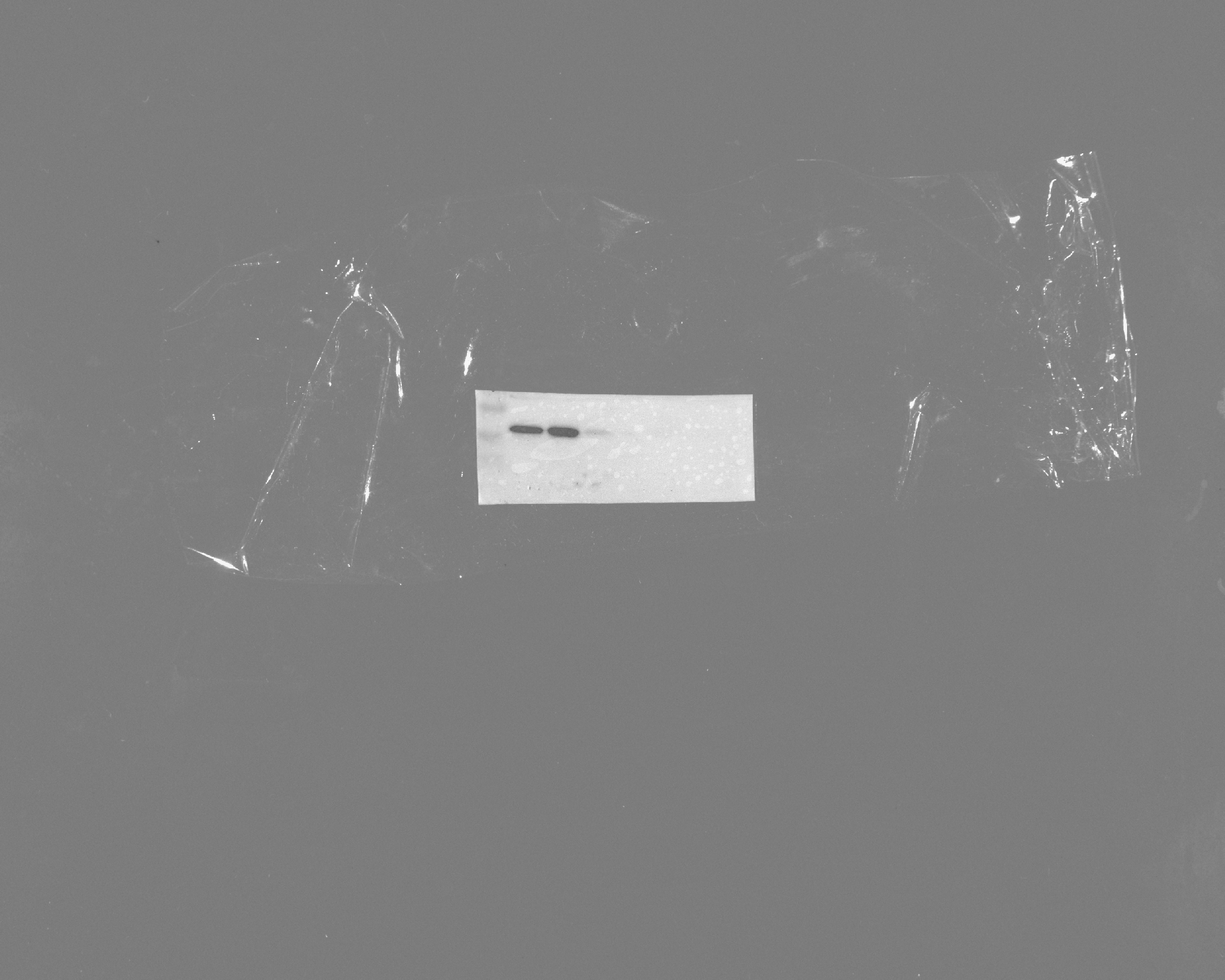

Supplement: Supplementary file 5 — Source data Fig. 1 [file 44319_2026_744_MOESM5_ESM.zip › Figure EV4/EV4A hTERT-RPE + EPZ-719/2025-11-21 rpe h3k36m3(Composite).jpg]

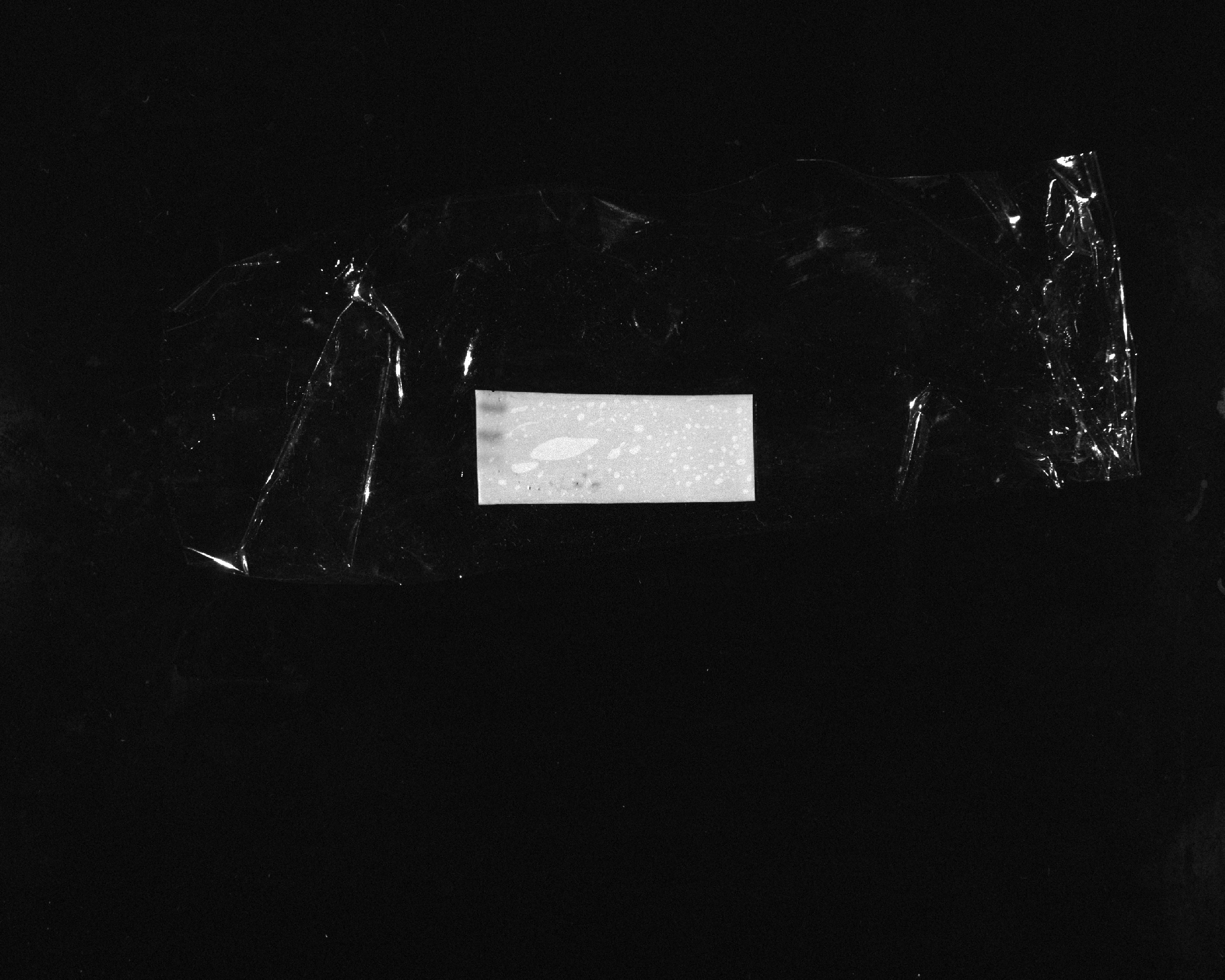

Supplement: Supplementary file 5 — Source data Fig. 1 [file 44319_2026_744_MOESM5_ESM.zip › Figure EV4/EV4A hTERT-RPE + EPZ-719/2025-11-21 rpe h3k36m3(Ponceau S).jpg]

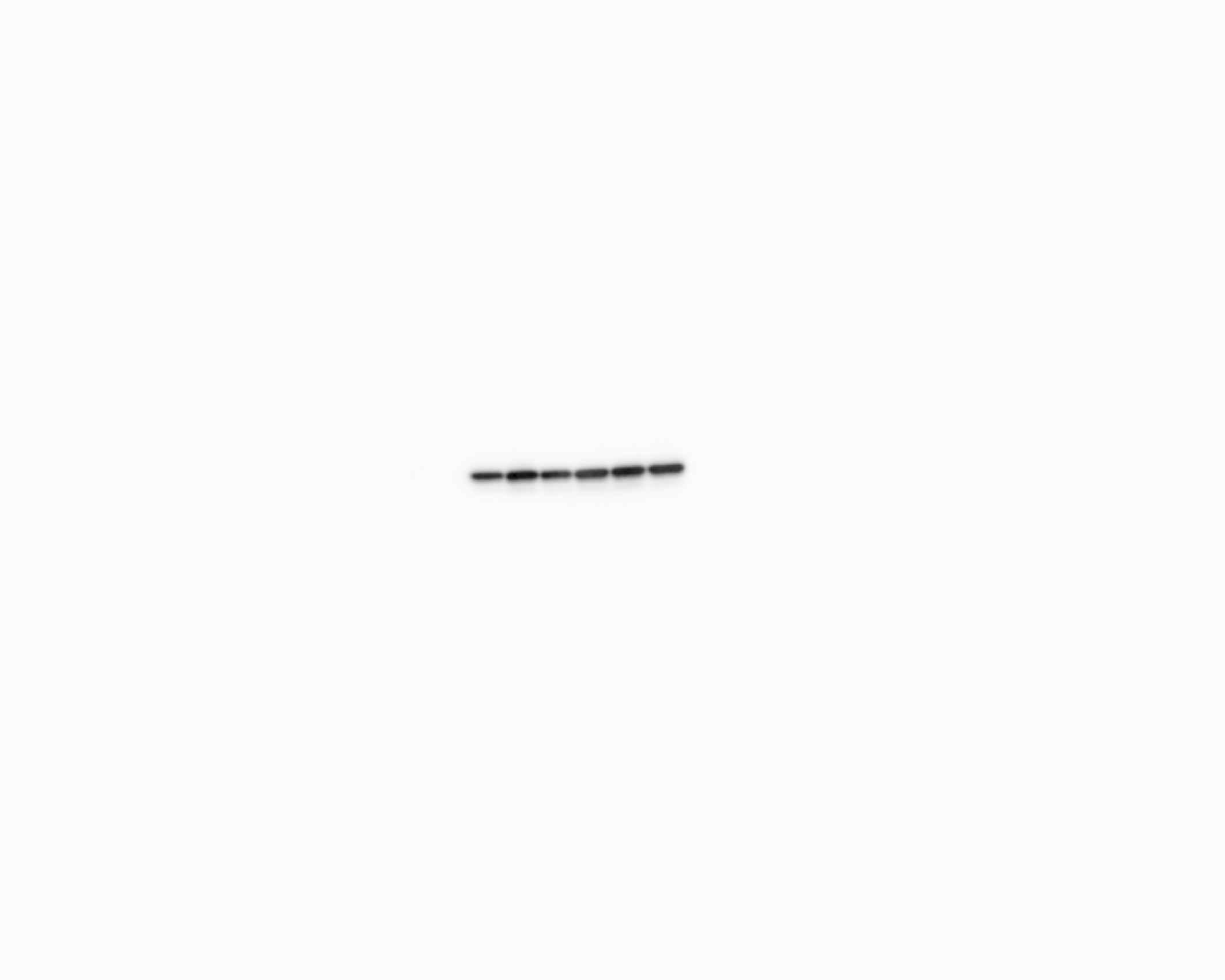

Supplement: Supplementary file 5 — Source data Fig. 1 [file 44319_2026_744_MOESM5_ESM.zip › Figure EV4/EV4A hTERT-RPE + EPZ-719/2025-11-27 rpe h3(Chemiluminescence).jpg]

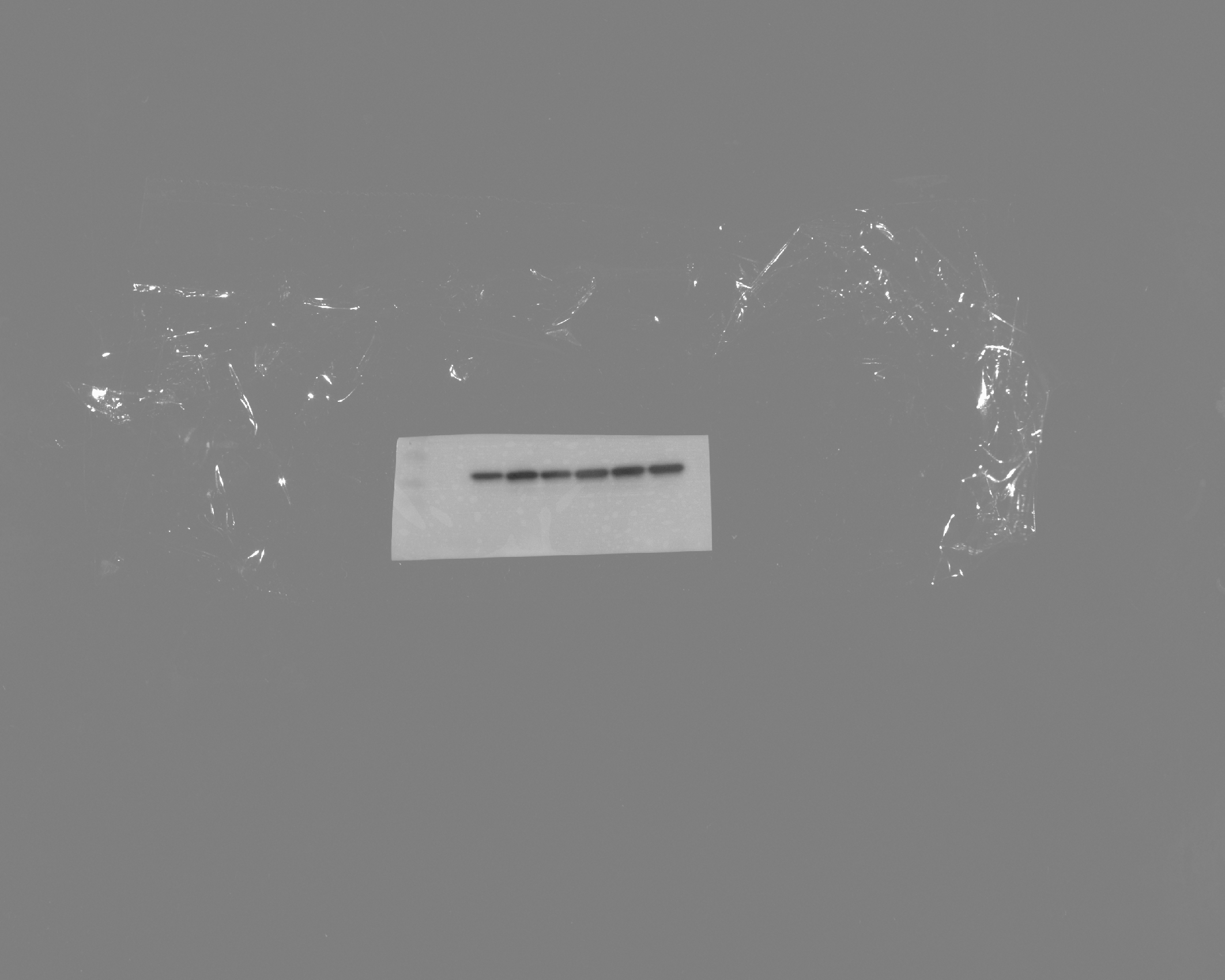

Supplement: Supplementary file 5 — Source data Fig. 1 [file 44319_2026_744_MOESM5_ESM.zip › Figure EV4/EV4A hTERT-RPE + EPZ-719/2025-11-27 rpe h3(Composite).jpg]

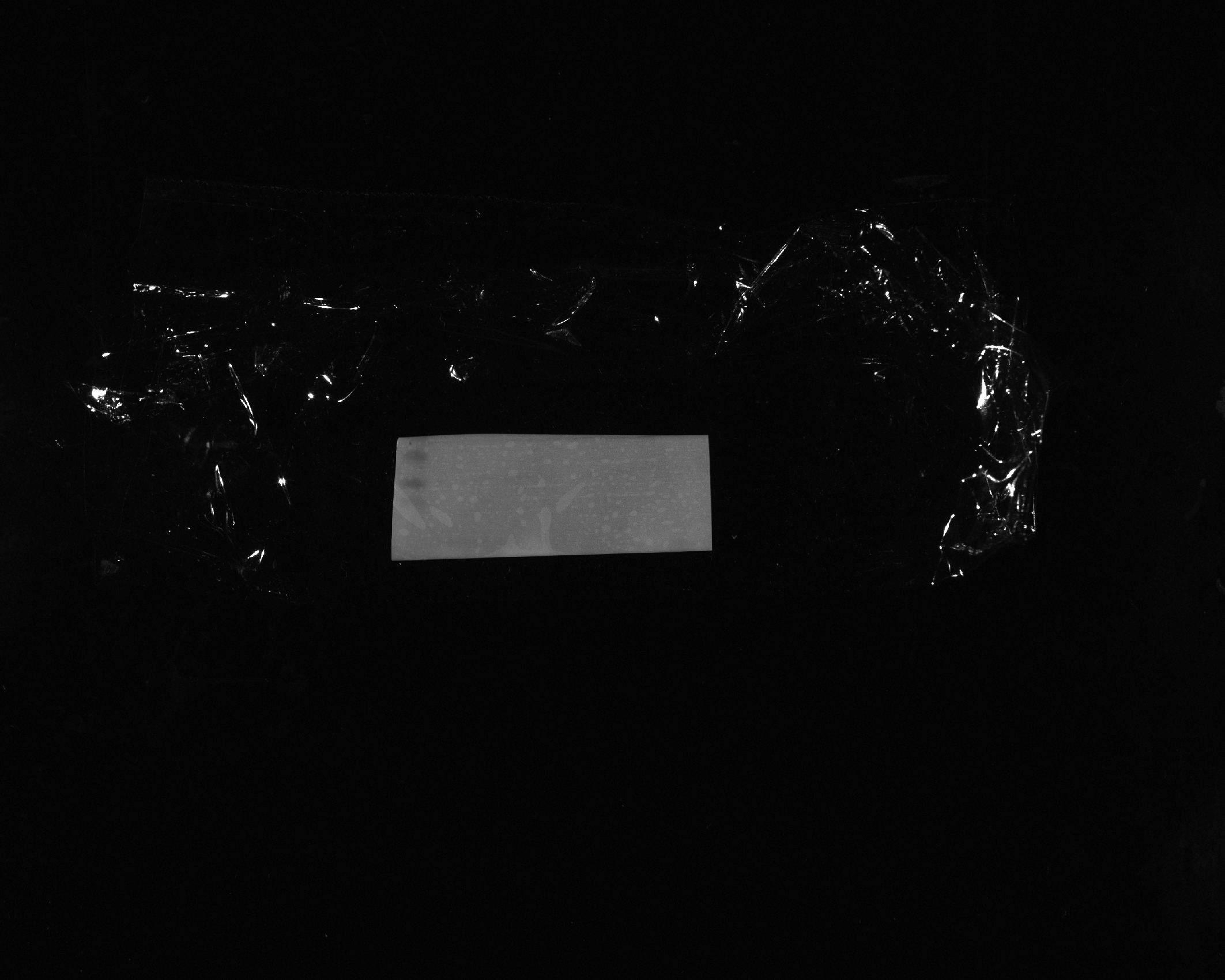

Supplement: Supplementary file 5 — Source data Fig. 1 [file 44319_2026_744_MOESM5_ESM.zip › Figure EV4/EV4A hTERT-RPE + EPZ-719/2025-11-27 rpe h3(Ponceau S).jpg]

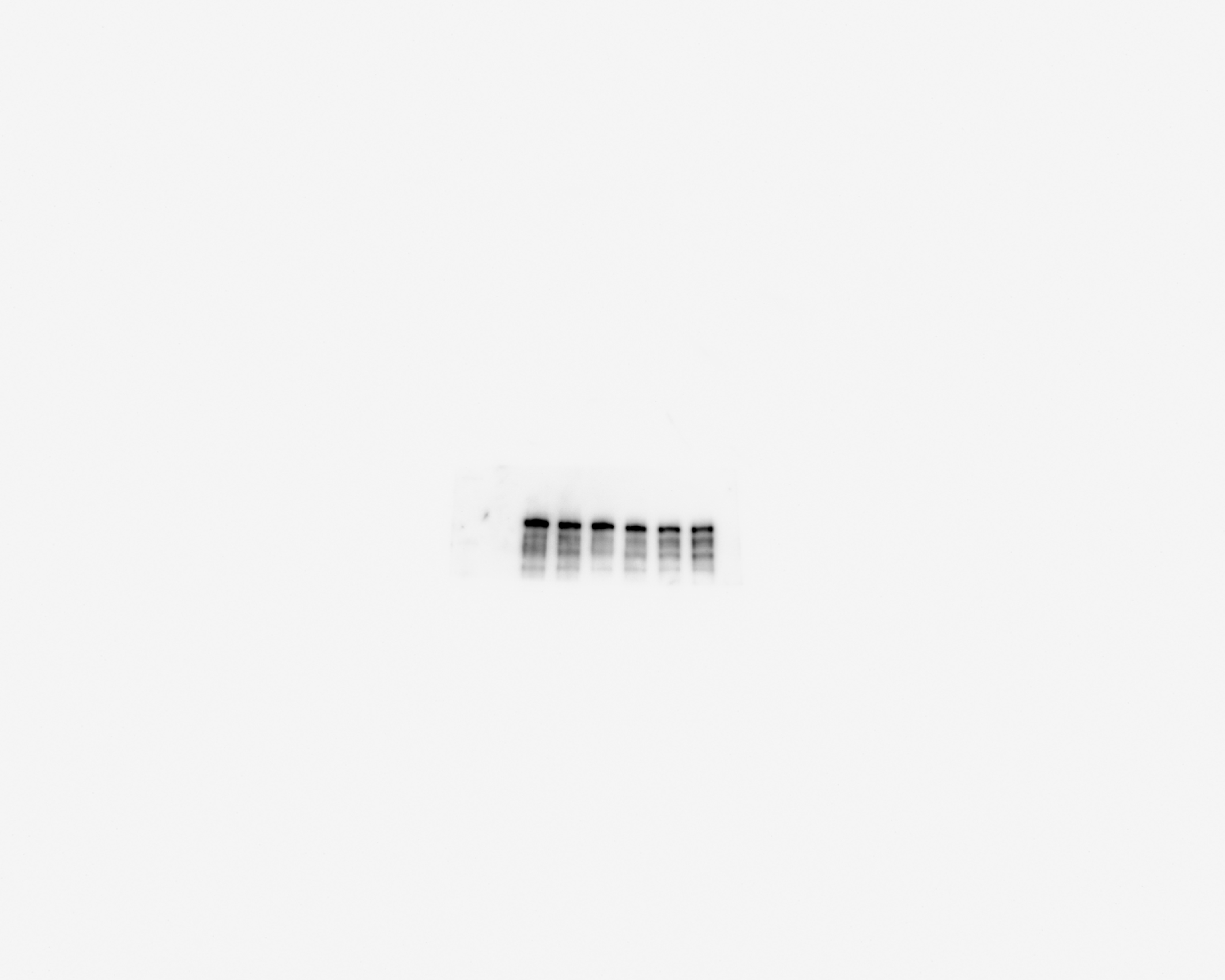

Supplement: Supplementary file 5 — Source data Fig. 1 [file 44319_2026_744_MOESM5_ESM.zip › Figure EV4/EV4A hTERT-RPE + EPZ-719/2025-11-27 setd2(Chemiluminescence).jpg]

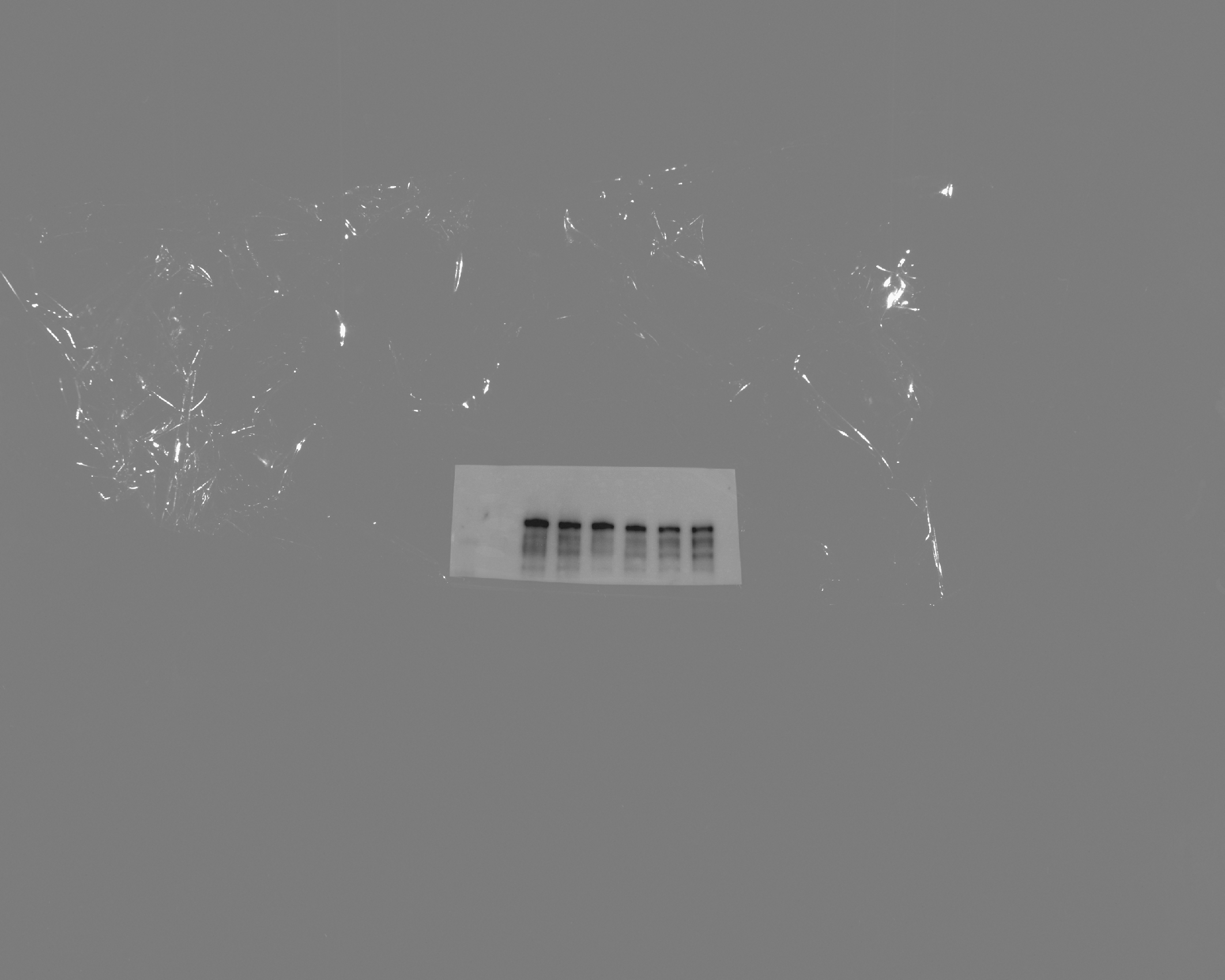

Supplement: Supplementary file 5 — Source data Fig. 1 [file 44319_2026_744_MOESM5_ESM.zip › Figure EV4/EV4A hTERT-RPE + EPZ-719/2025-11-27 setd2(Composite).jpg]

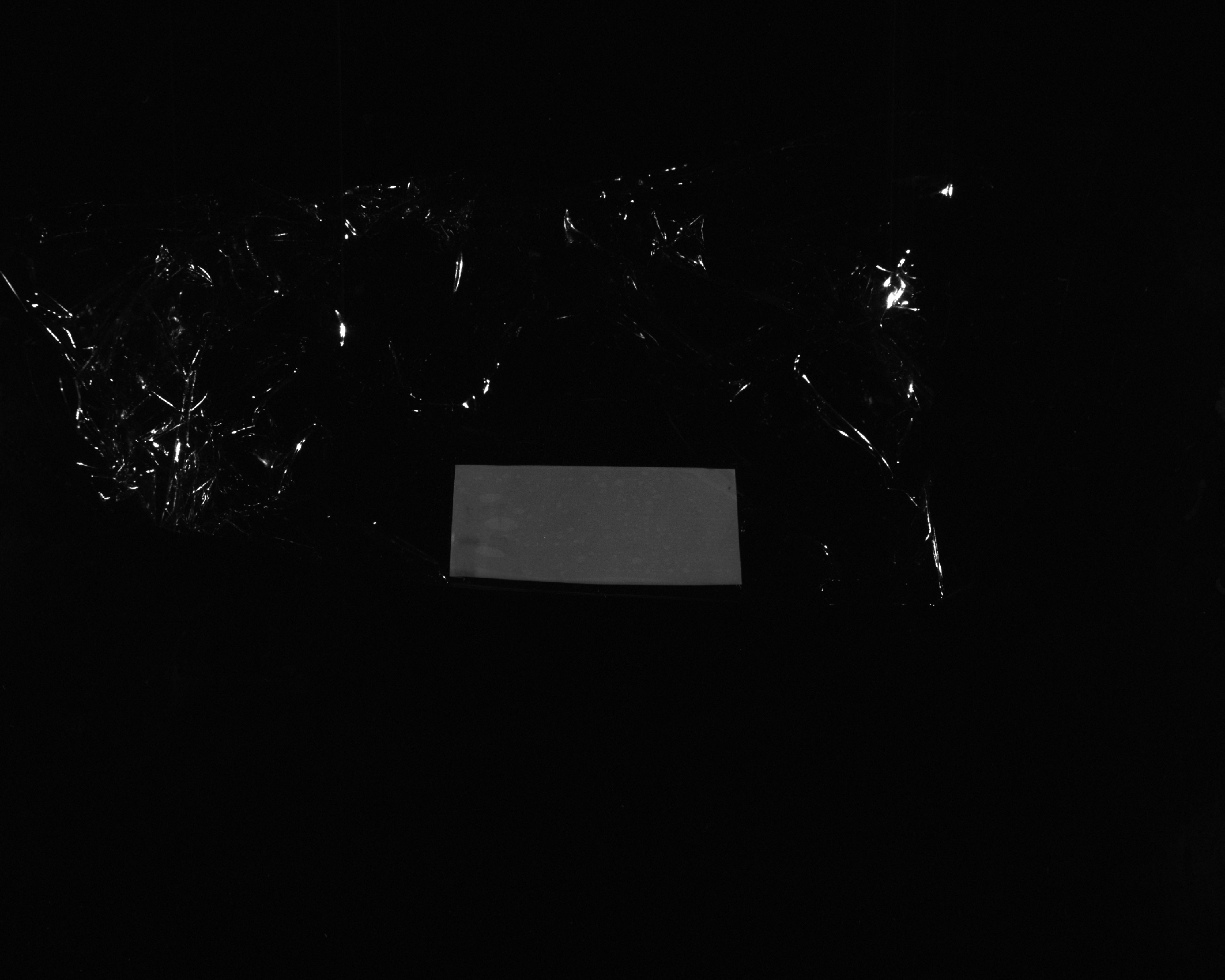

Supplement: Supplementary file 5 — Source data Fig. 1 [file 44319_2026_744_MOESM5_ESM.zip › Figure EV4/EV4A hTERT-RPE + EPZ-719/2025-11-27 setd2(Ponceau S).jpg]

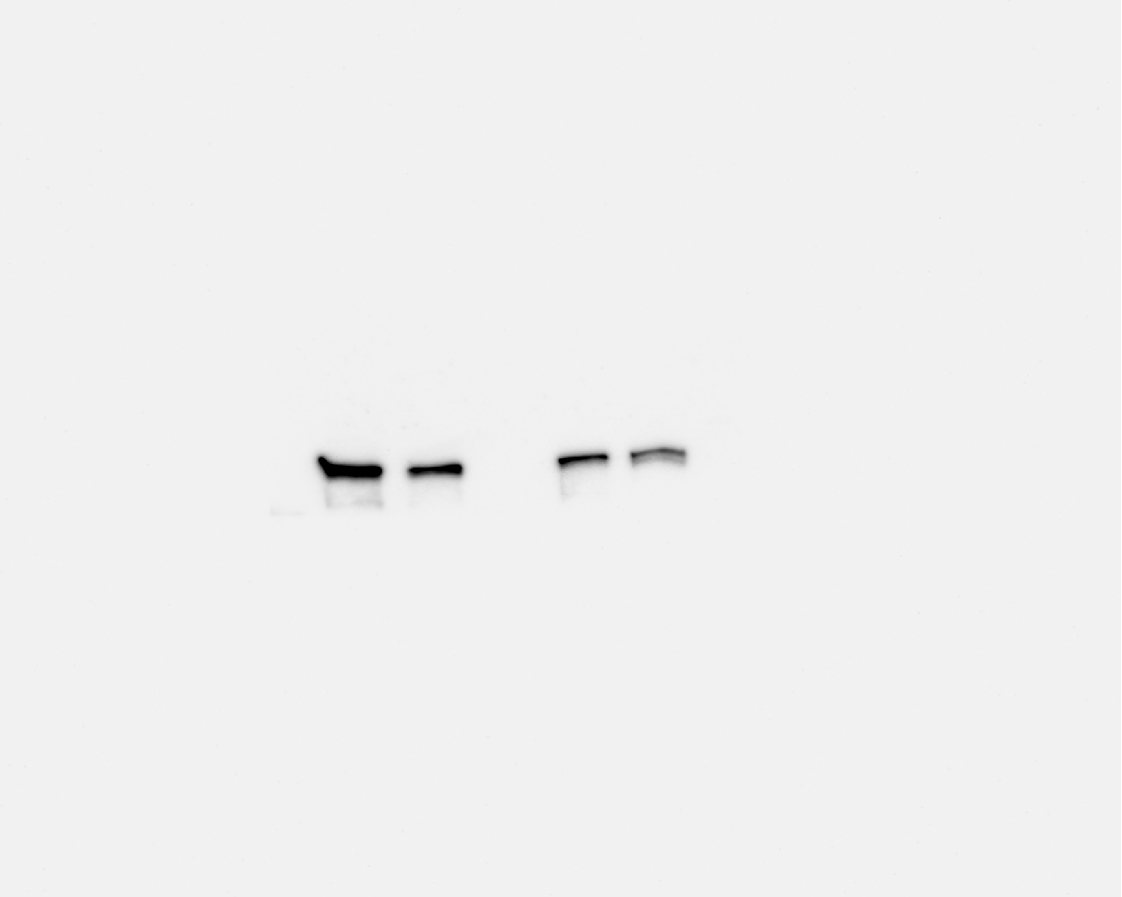

Supplement: Supplementary file 6 — Source data Fig. 4 [file 44319_2026_744_MOESM6_ESM.zip › Figure 1/1H/RCC/2024-05-08 achn,a498 setd2(Chemiluminescence).jpg]

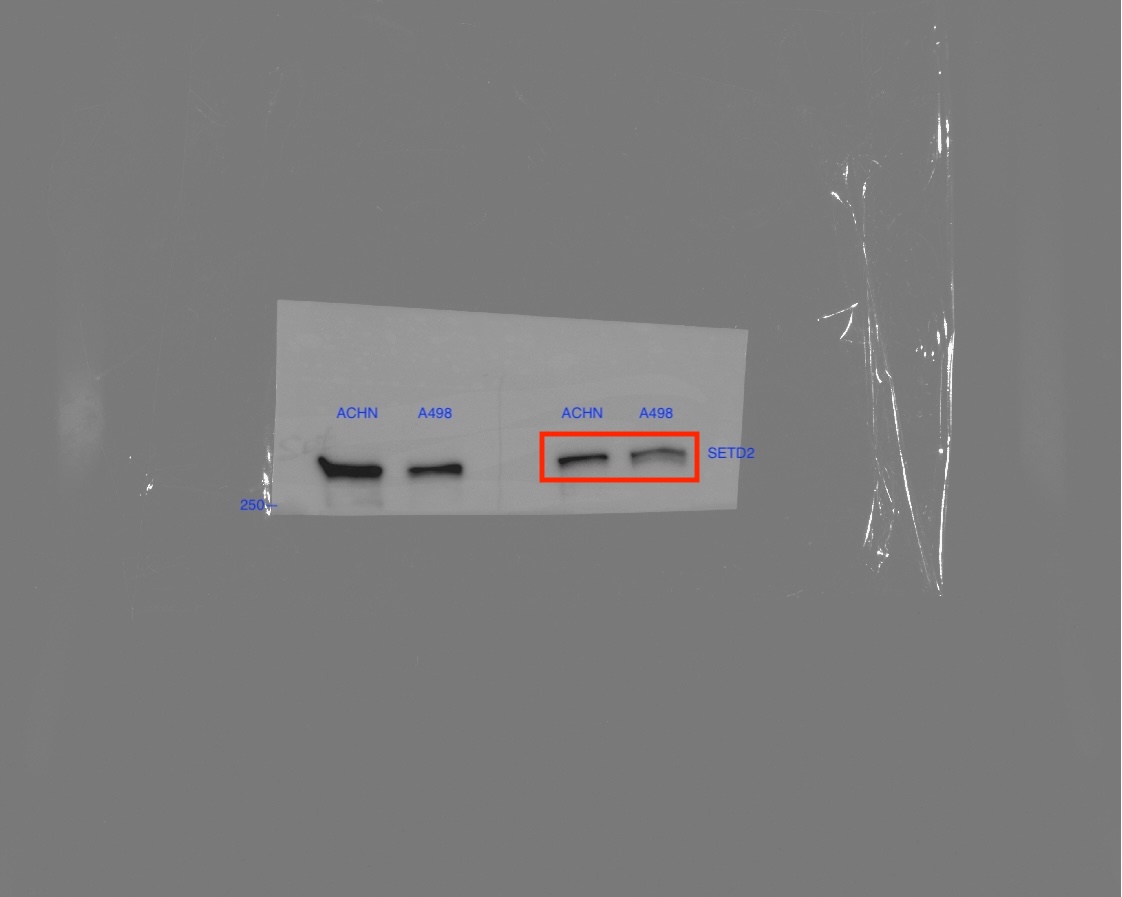

Supplement: Supplementary file 6 — Source data Fig. 4 [file 44319_2026_744_MOESM6_ESM.zip › Figure 1/1H/RCC/2024-05-08 achn,a498 setd2(Composite).jpg]

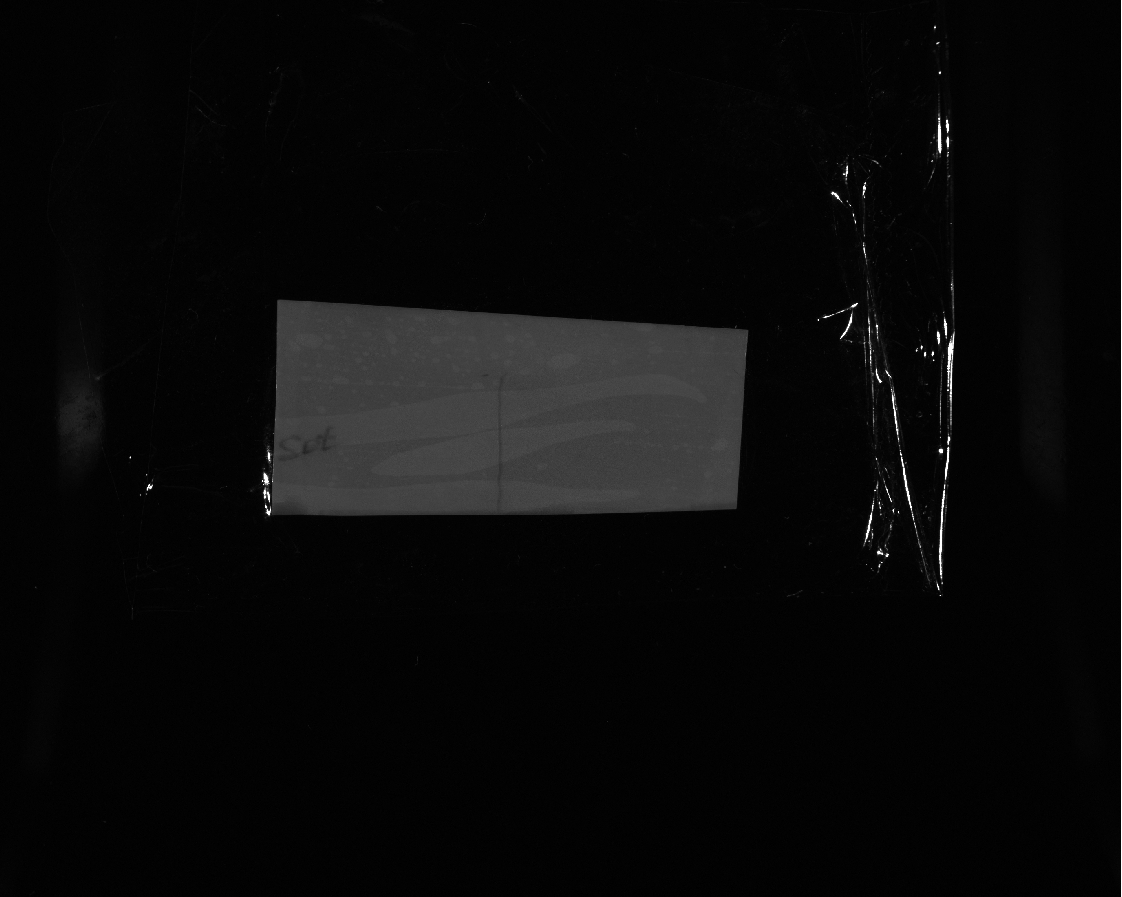

Supplement: Supplementary file 6 — Source data Fig. 4 [file 44319_2026_744_MOESM6_ESM.zip › Figure 1/1H/RCC/2024-05-08 achn,a498 setd2(Ponceau S).jpg]

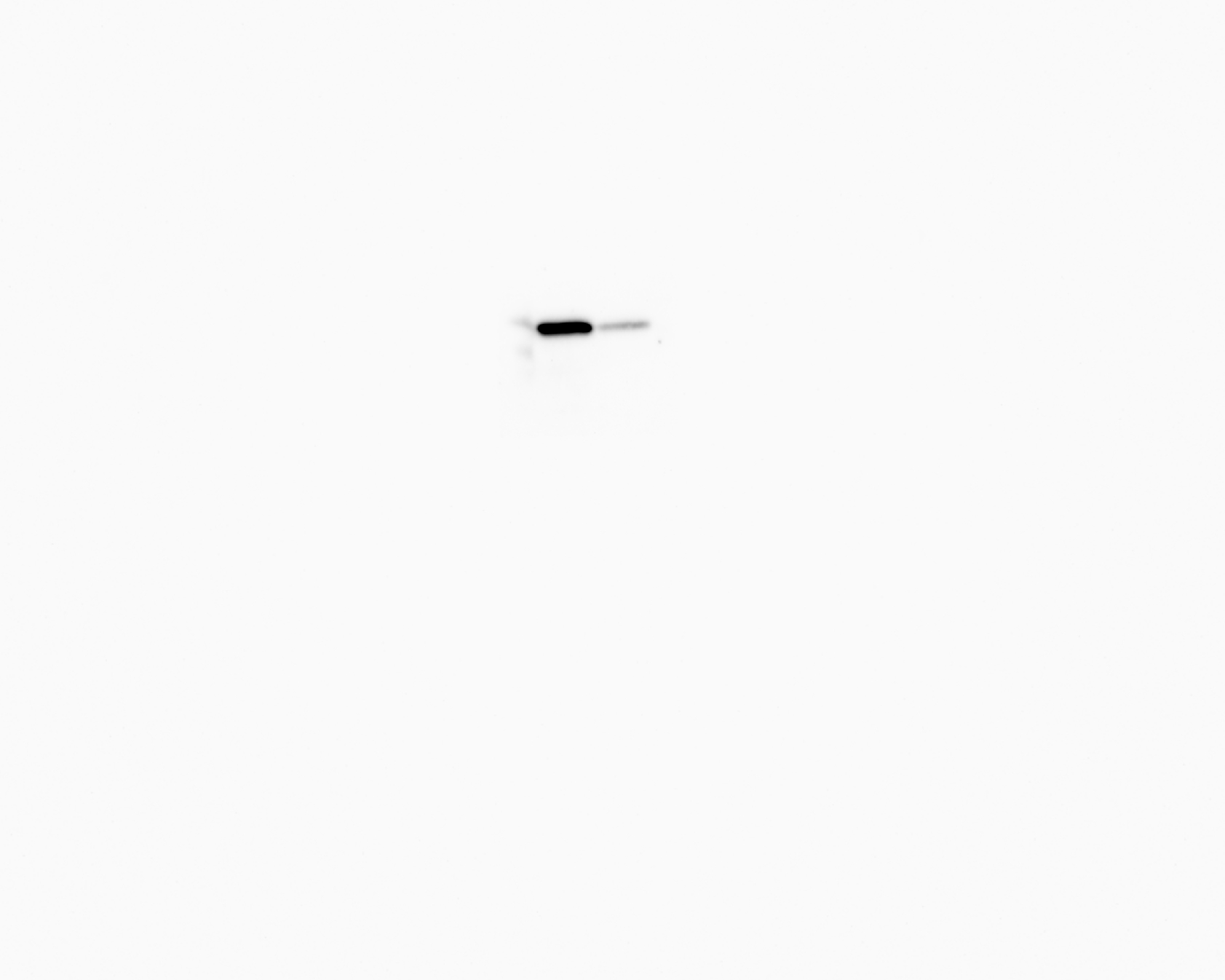

Supplement: Supplementary file 6 — Source data Fig. 4 [file 44319_2026_744_MOESM6_ESM.zip › Figure 1/1H/RCC/2024-12-03 achn,a498 k36m3(Chemiluminescence).jpg]

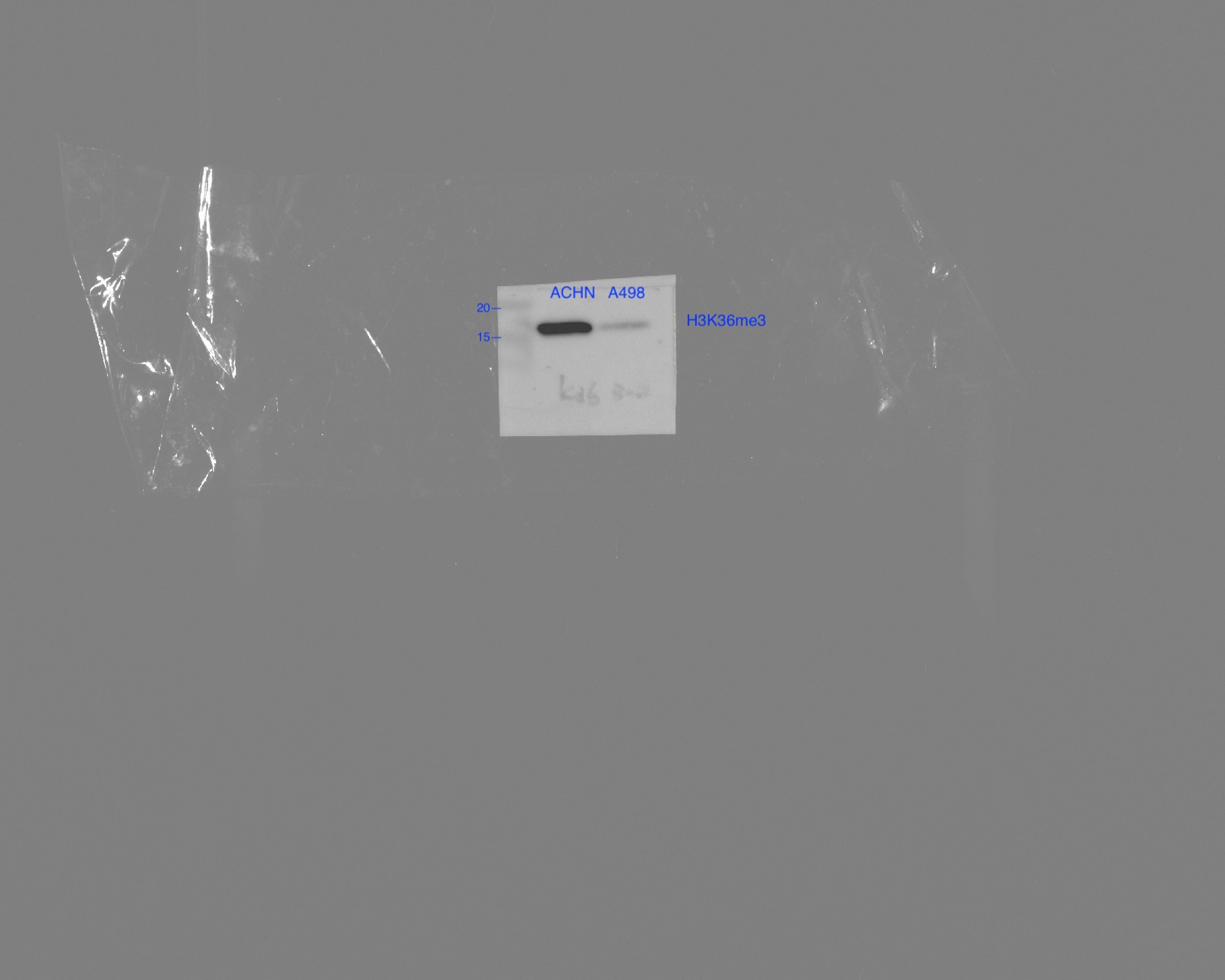

Supplement: Supplementary file 6 — Source data Fig. 4 [file 44319_2026_744_MOESM6_ESM.zip › Figure 1/1H/RCC/2024-12-03 achn,a498 k36m3(Composite).jpg]

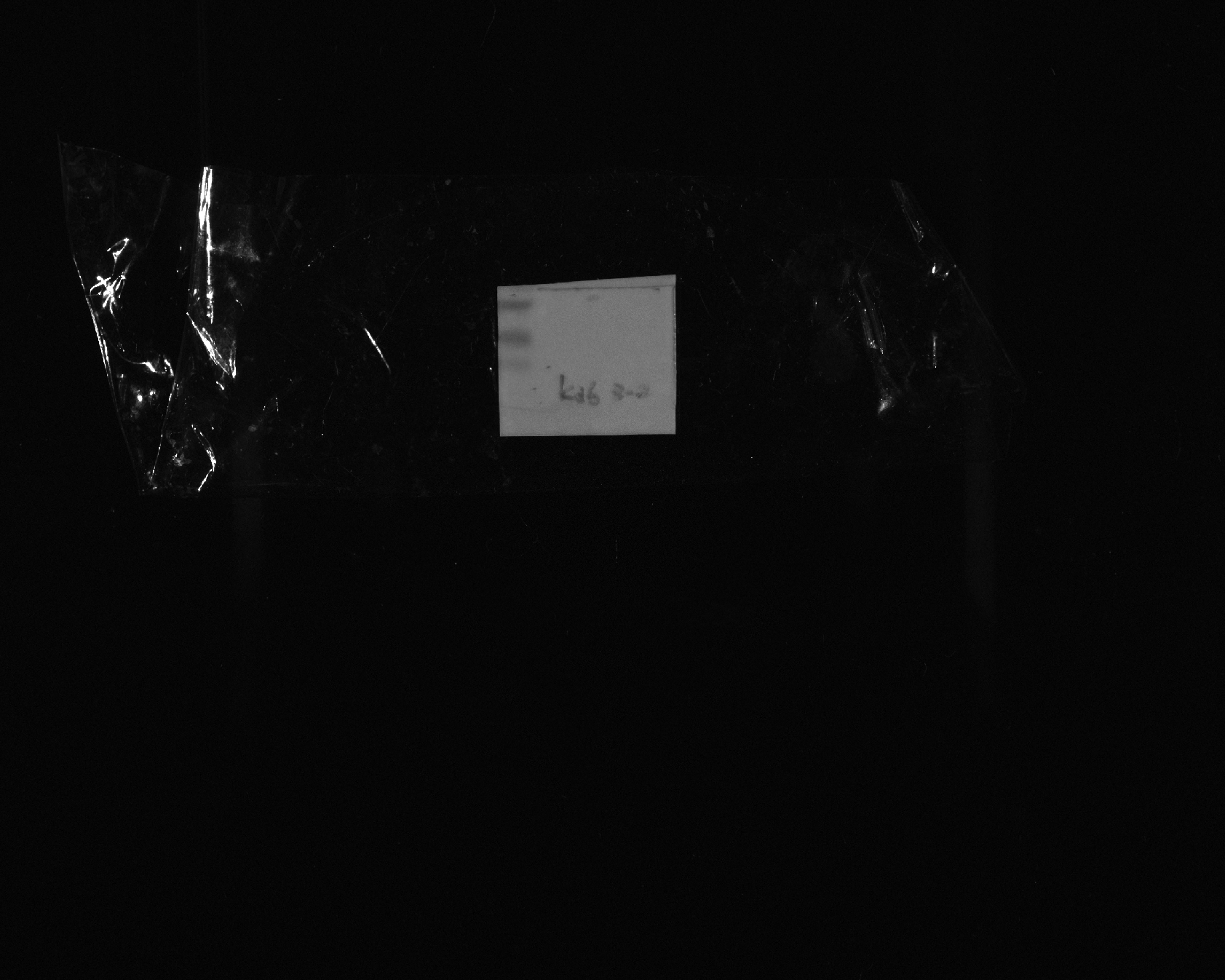

Supplement: Supplementary file 6 — Source data Fig. 4 [file 44319_2026_744_MOESM6_ESM.zip › Figure 1/1H/RCC/2024-12-03 achn,a498 k36m3(Ponceau S).jpg]

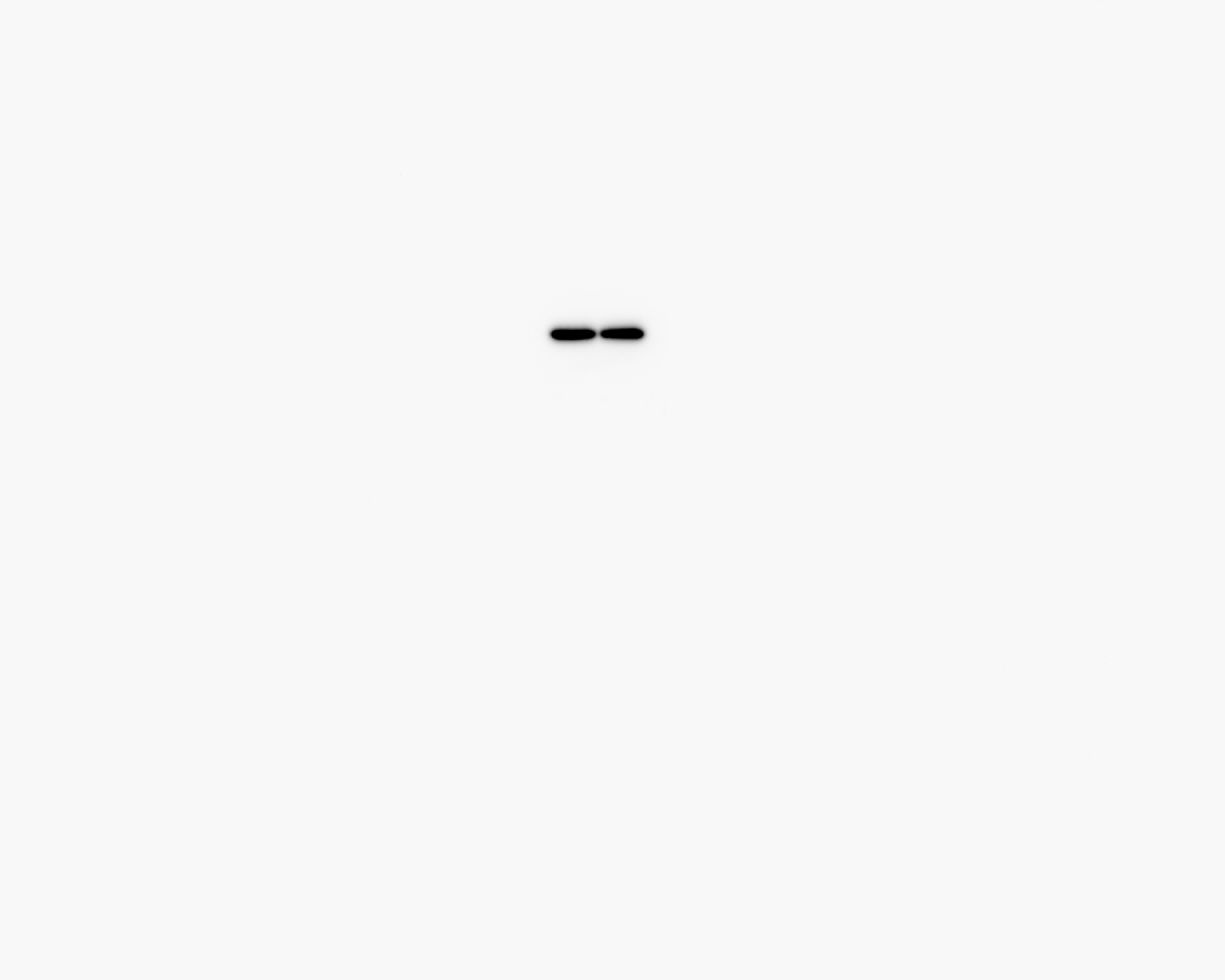

Supplement: Supplementary file 6 — Source data Fig. 4 [file 44319_2026_744_MOESM6_ESM.zip › Figure 1/1H/RCC/2024-12-10 achn,a498 rep4 h3-2(Chemiluminescence).jpg]

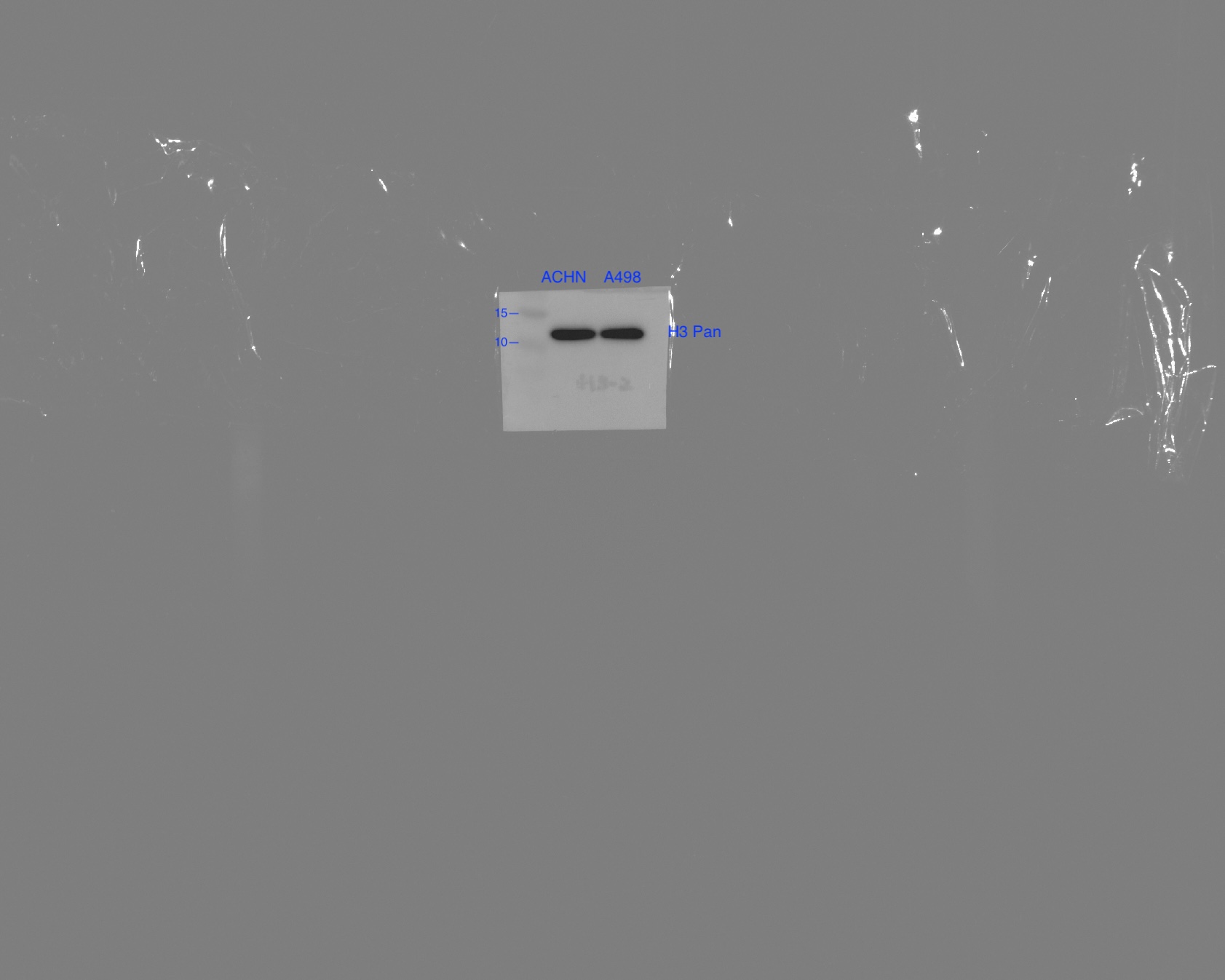

Supplement: Supplementary file 6 — Source data Fig. 4 [file 44319_2026_744_MOESM6_ESM.zip › Figure 1/1H/RCC/2024-12-10 achn,a498 rep4 h3-2(Composite).jpg]

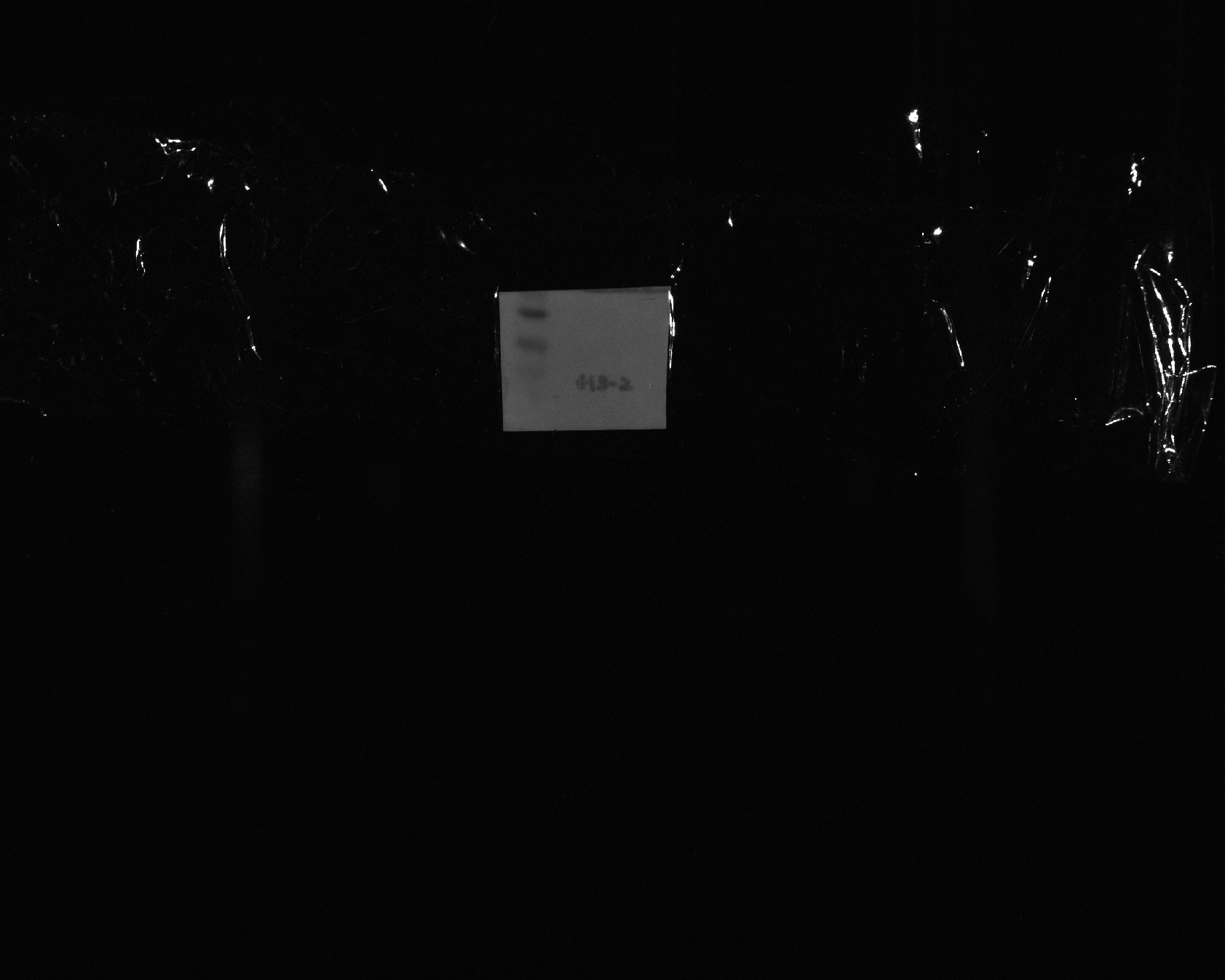

Supplement: Supplementary file 6 — Source data Fig. 4 [file 44319_2026_744_MOESM6_ESM.zip › Figure 1/1H/RCC/2024-12-10 achn,a498 rep4 h3-2(Ponceau S).jpg]

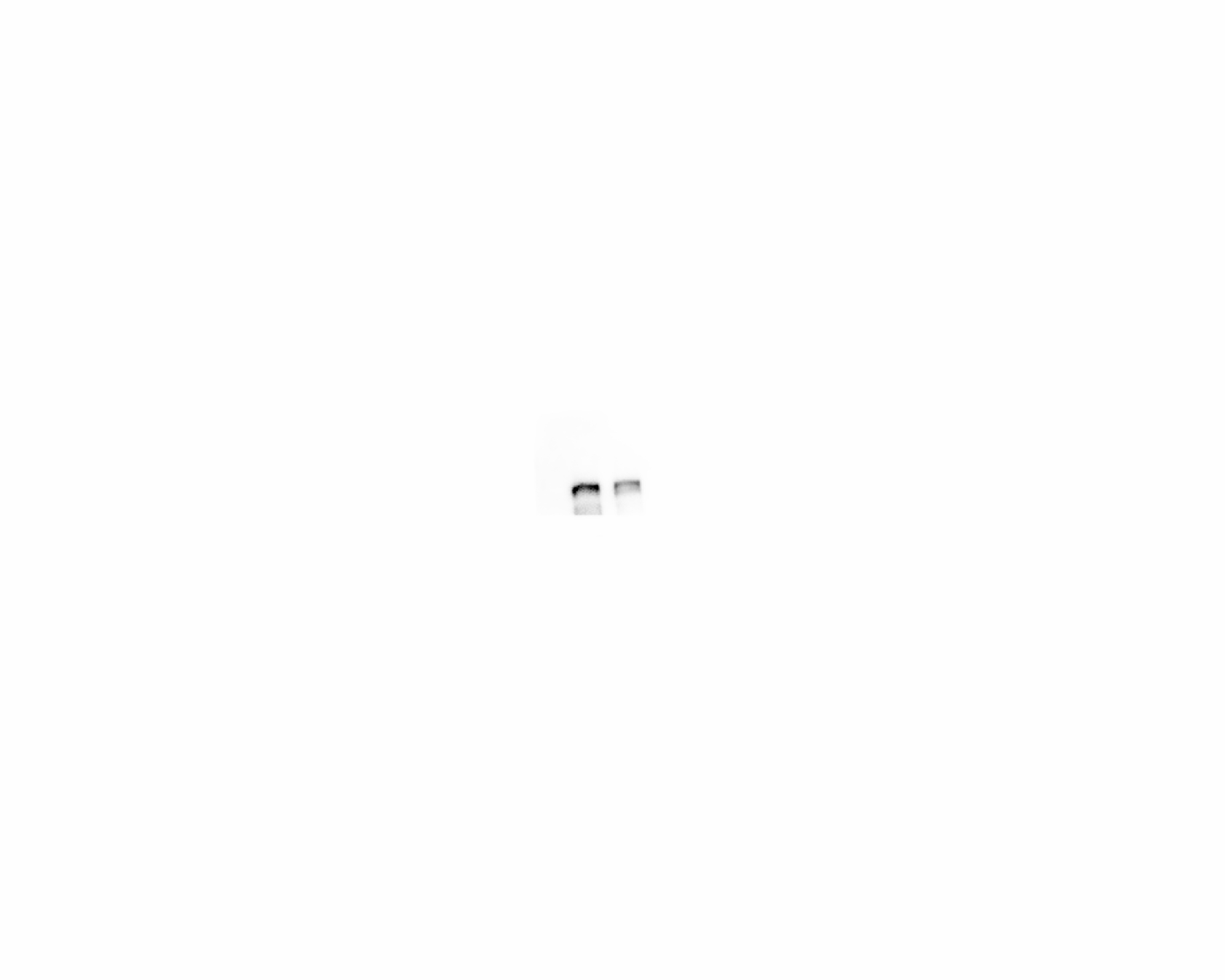

Supplement: Supplementary file 6 — Source data Fig. 4 [file 44319_2026_744_MOESM6_ESM.zip › Figure 1/1H/RCC/2024-12-11 achn,a498 rep4 setd2(Chemiluminescence).jpg]

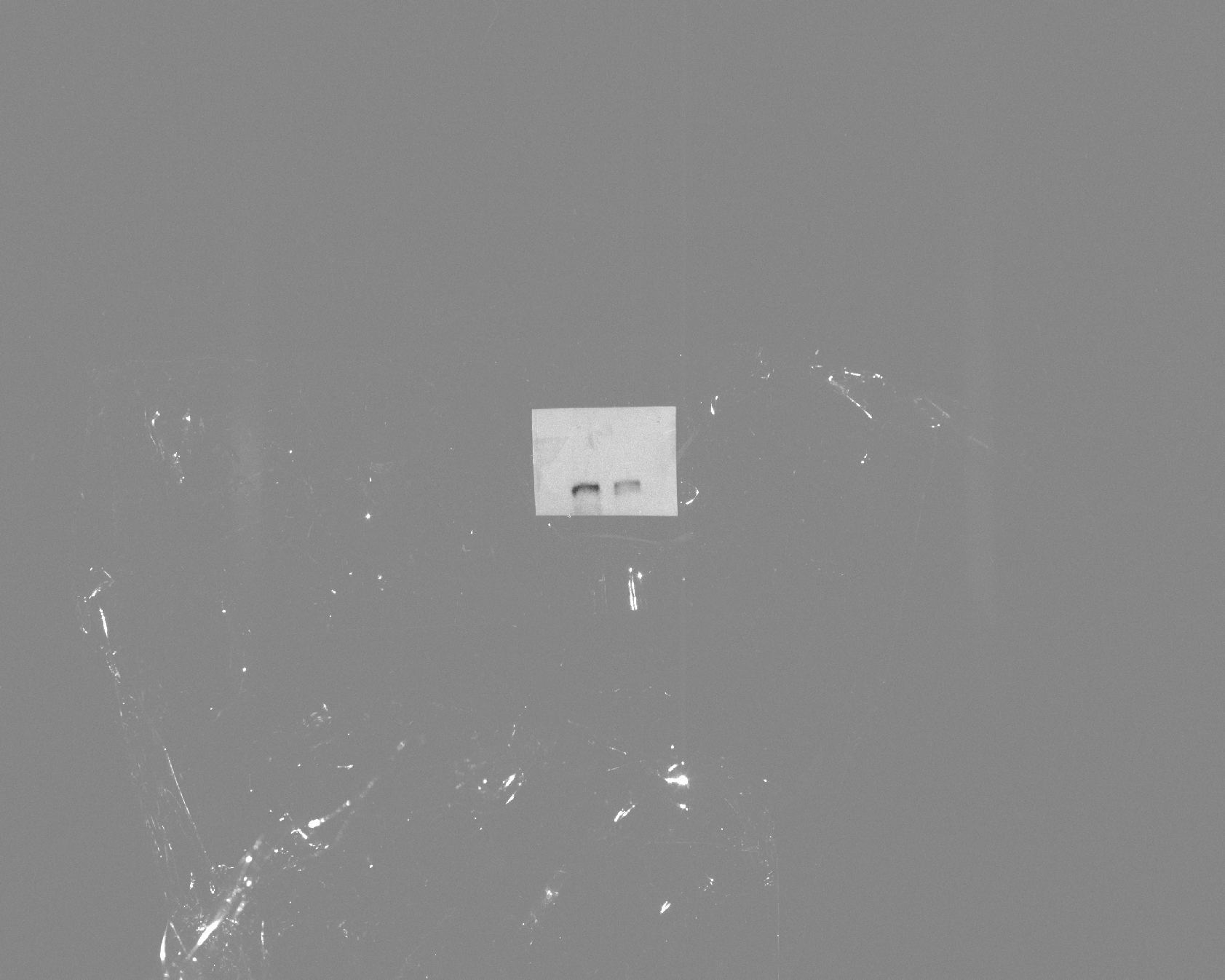

Supplement: Supplementary file 6 — Source data Fig. 4 [file 44319_2026_744_MOESM6_ESM.zip › Figure 1/1H/RCC/2024-12-11 achn,a498 rep4 setd2(Composite).jpg]

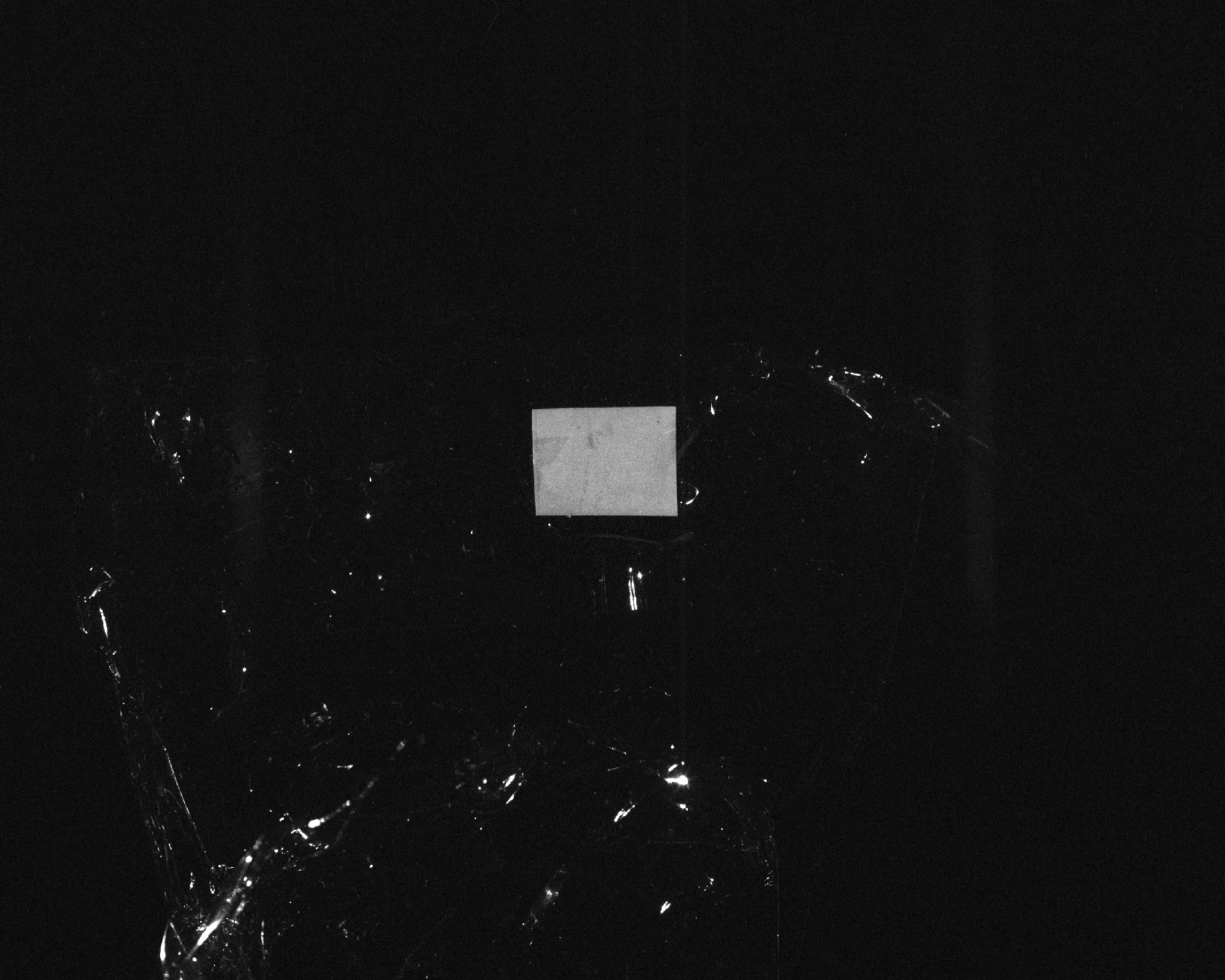

Supplement: Supplementary file 6 — Source data Fig. 4 [file 44319_2026_744_MOESM6_ESM.zip › Figure 1/1H/RCC/2024-12-11 achn,a498 rep4 setd2(Ponceau S).jpg]

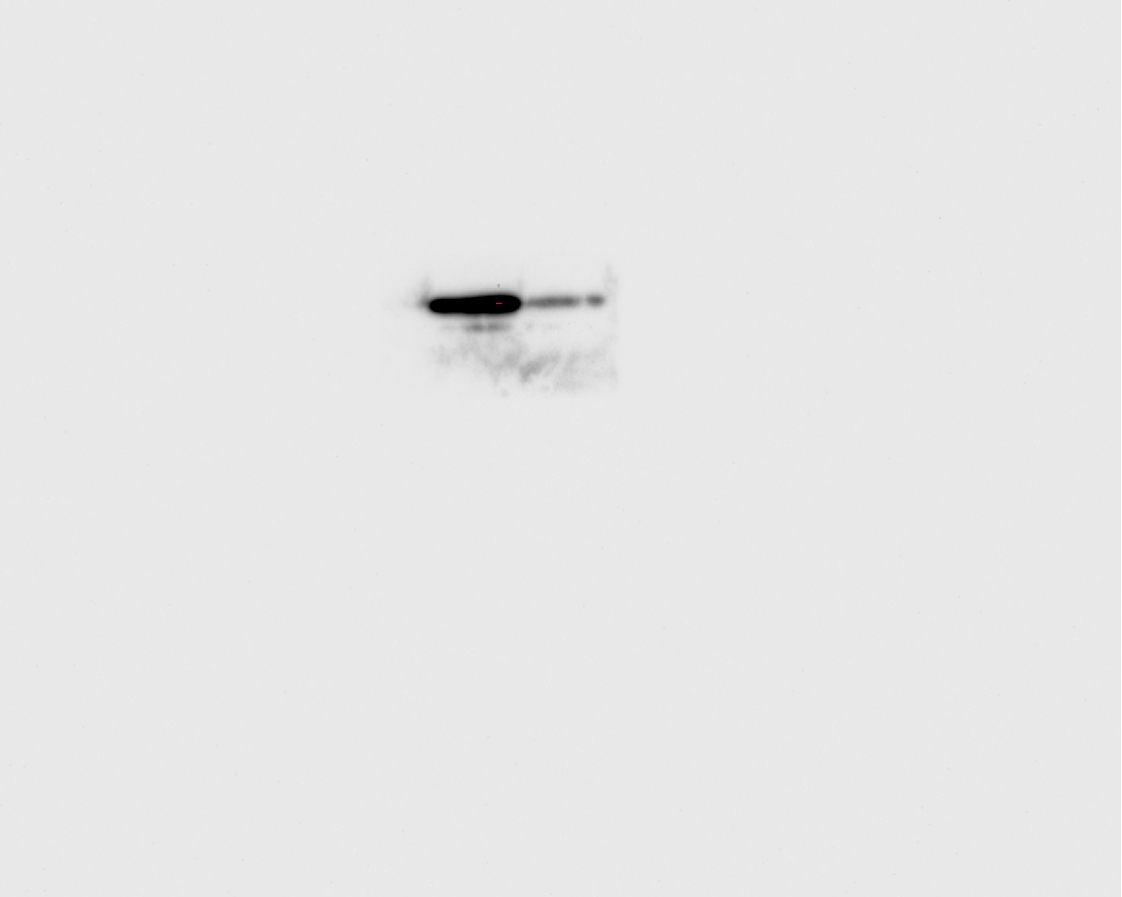

Supplement: Supplementary file 6 — Source data Fig. 4 [file 44319_2026_744_MOESM6_ESM.zip › Figure 1/1H/RCC/Other reps for quantification/2024-05-08 achn,a498 H3K36me3(Chemiluminescence).jpg]

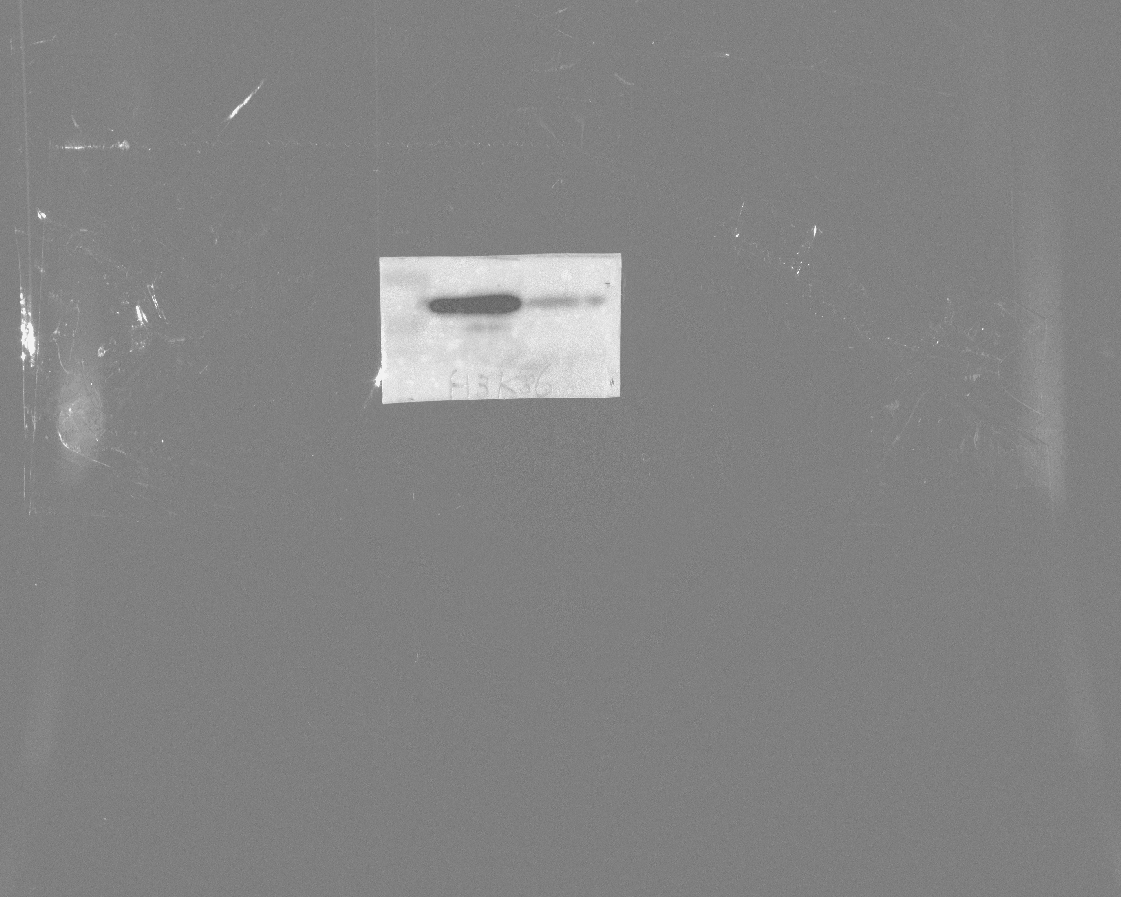

Supplement: Supplementary file 6 — Source data Fig. 4 [file 44319_2026_744_MOESM6_ESM.zip › Figure 1/1H/RCC/Other reps for quantification/2024-05-08 achn,a498 H3K36me3(Composite).jpg]

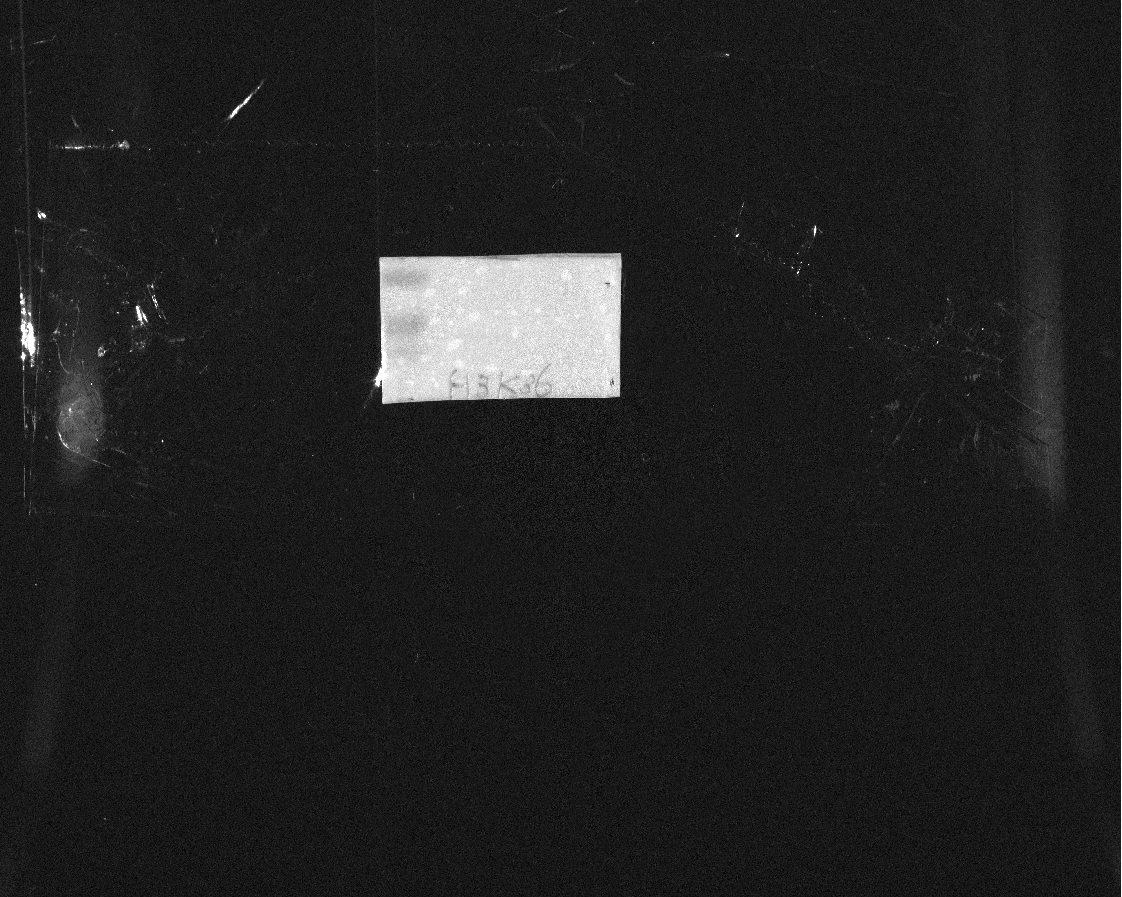

Supplement: Supplementary file 6 — Source data Fig. 4 [file 44319_2026_744_MOESM6_ESM.zip › Figure 1/1H/RCC/Other reps for quantification/2024-05-08 achn,a498 H3K36me3(Ponceau S).jpg]

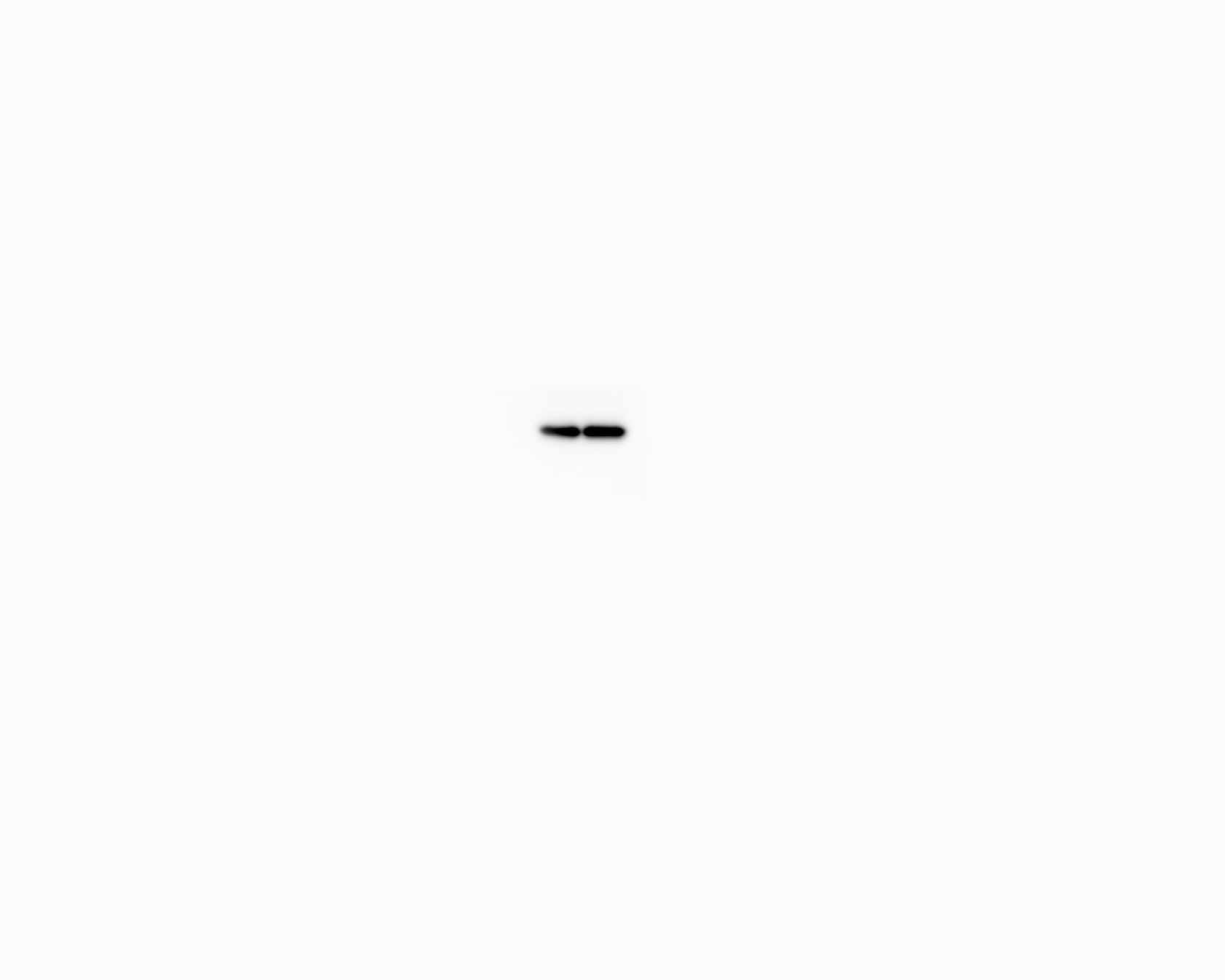

Supplement: Supplementary file 6 — Source data Fig. 4 [file 44319_2026_744_MOESM6_ESM.zip › Figure 1/1H/RCC/Other reps for quantification/2024-12-05 achn,a498 r2 h3(Chemiluminescence).jpg]

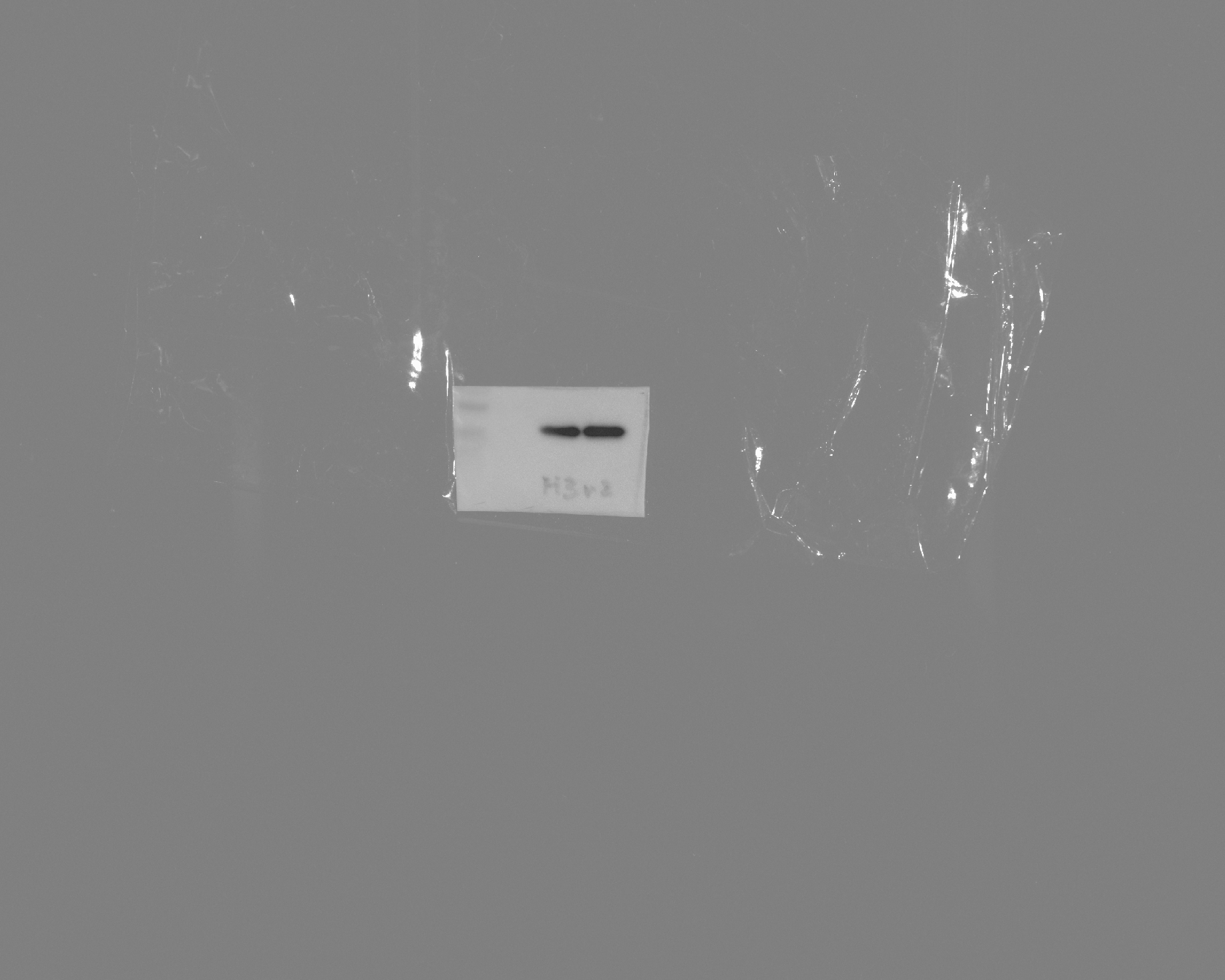

Supplement: Supplementary file 6 — Source data Fig. 4 [file 44319_2026_744_MOESM6_ESM.zip › Figure 1/1H/RCC/Other reps for quantification/2024-12-05 achn,a498 r2 h3(Composite).jpg]

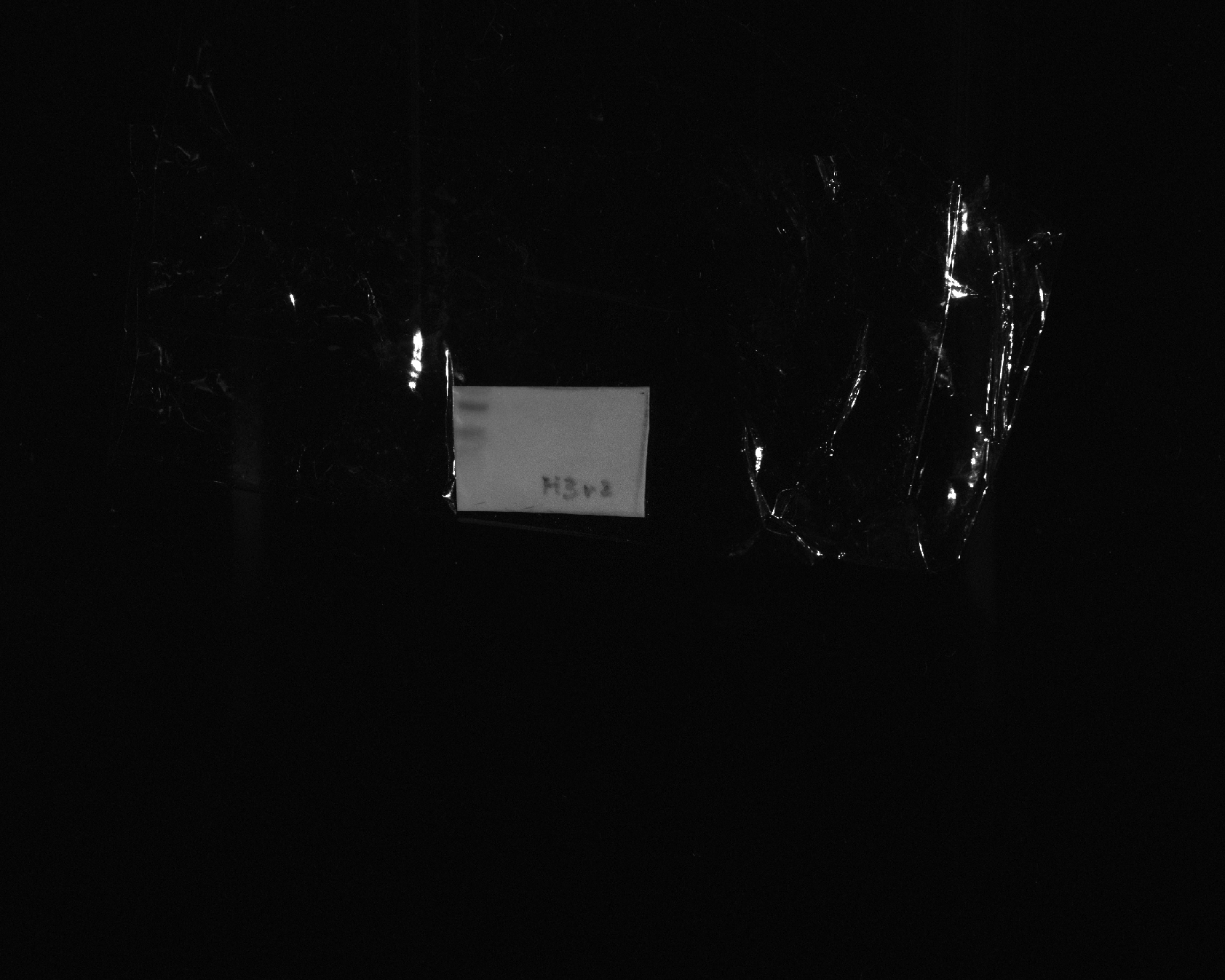

Supplement: Supplementary file 6 — Source data Fig. 4 [file 44319_2026_744_MOESM6_ESM.zip › Figure 1/1H/RCC/Other reps for quantification/2024-12-05 achn,a498 r2 h3(Ponceau S).jpg]

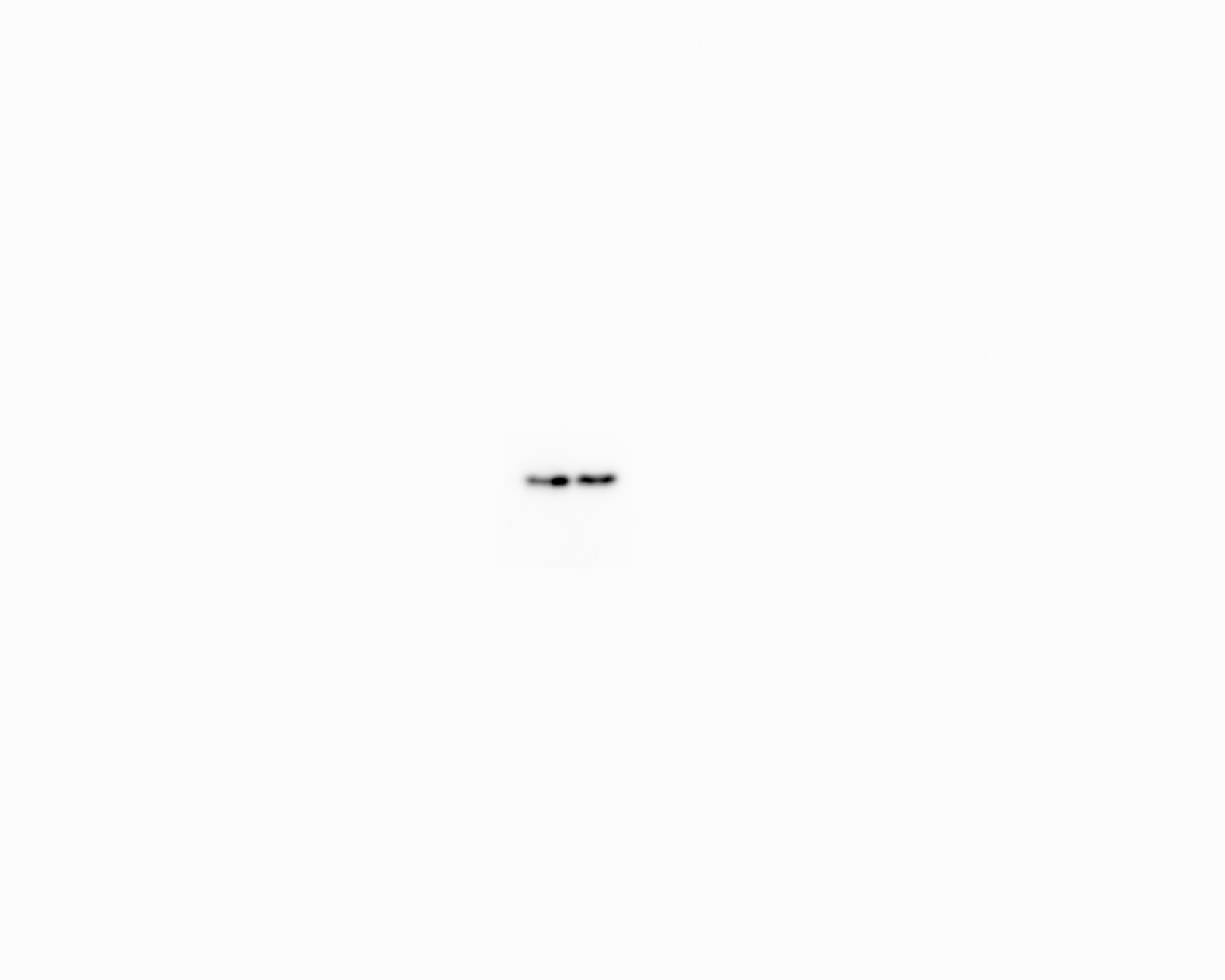

Supplement: Supplementary file 6 — Source data Fig. 4 [file 44319_2026_744_MOESM6_ESM.zip › Figure 1/1H/RCC/Other reps for quantification/2024-12-05 achn,a498 r3 h3(Chemiluminescence).jpg]

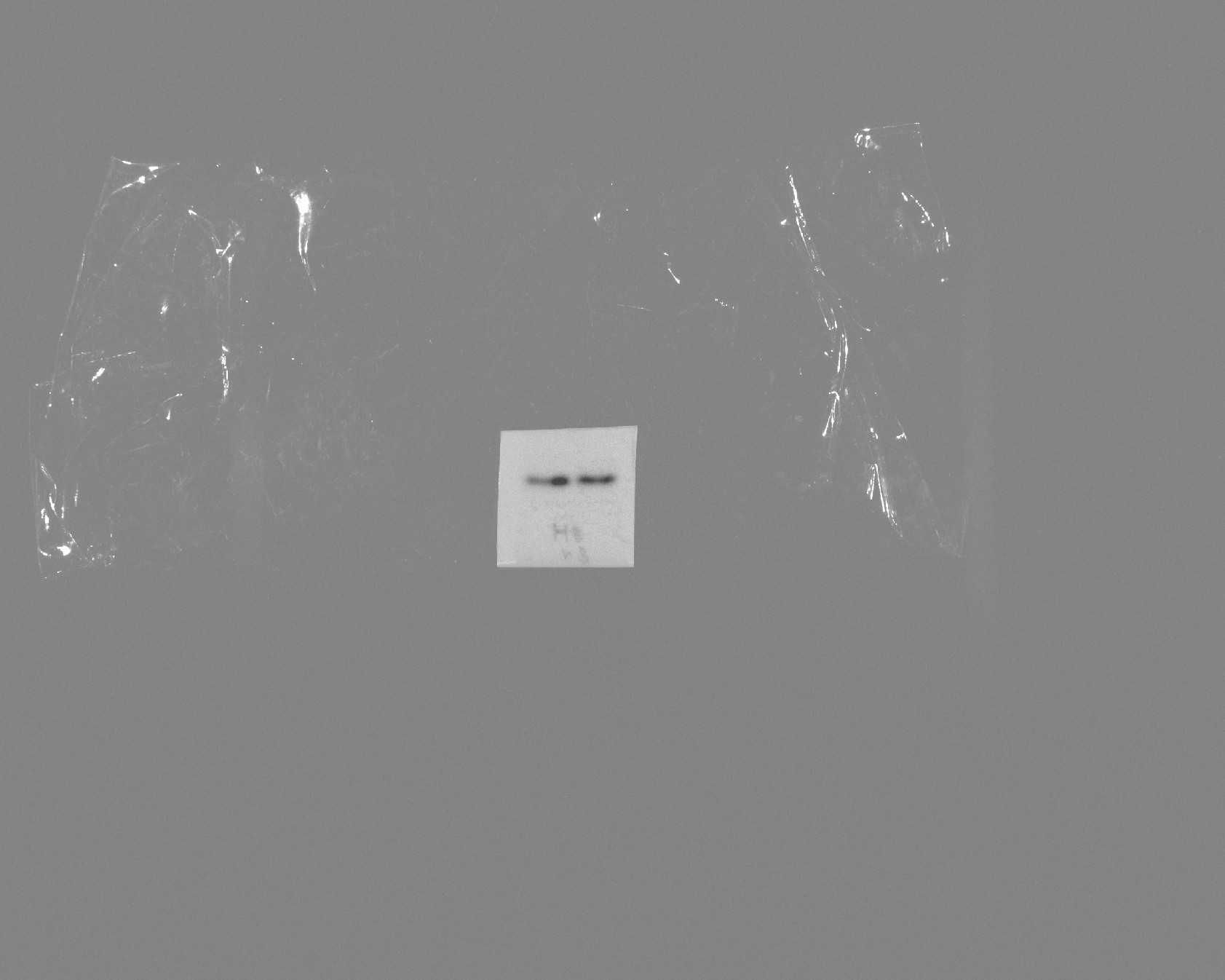

Supplement: Supplementary file 6 — Source data Fig. 4 [file 44319_2026_744_MOESM6_ESM.zip › Figure 1/1H/RCC/Other reps for quantification/2024-12-05 achn,a498 r3 h3(Composite).jpg]

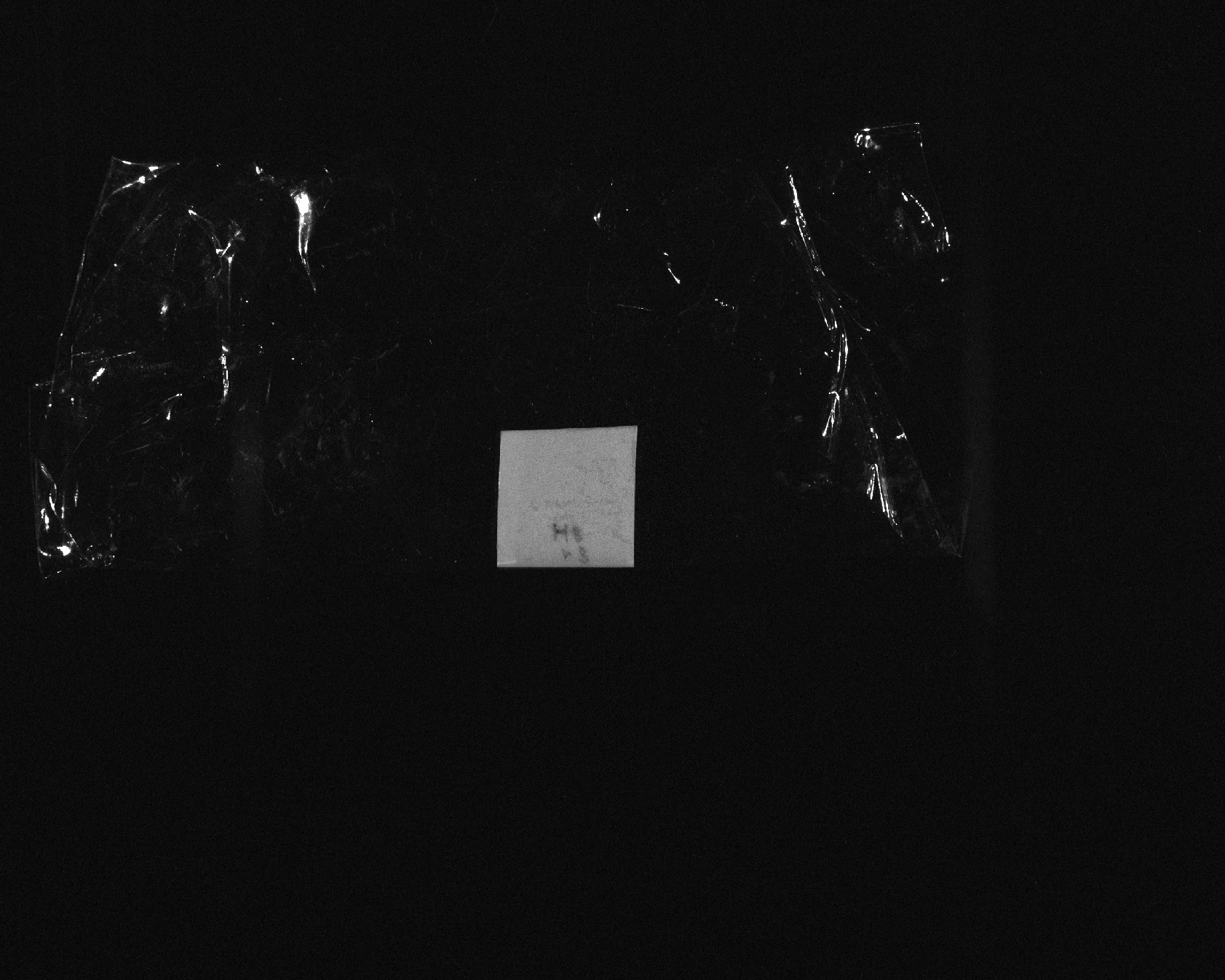

Supplement: Supplementary file 6 — Source data Fig. 4 [file 44319_2026_744_MOESM6_ESM.zip › Figure 1/1H/RCC/Other reps for quantification/2024-12-05 achn,a498 r3 h3(Ponceau S).jpg]

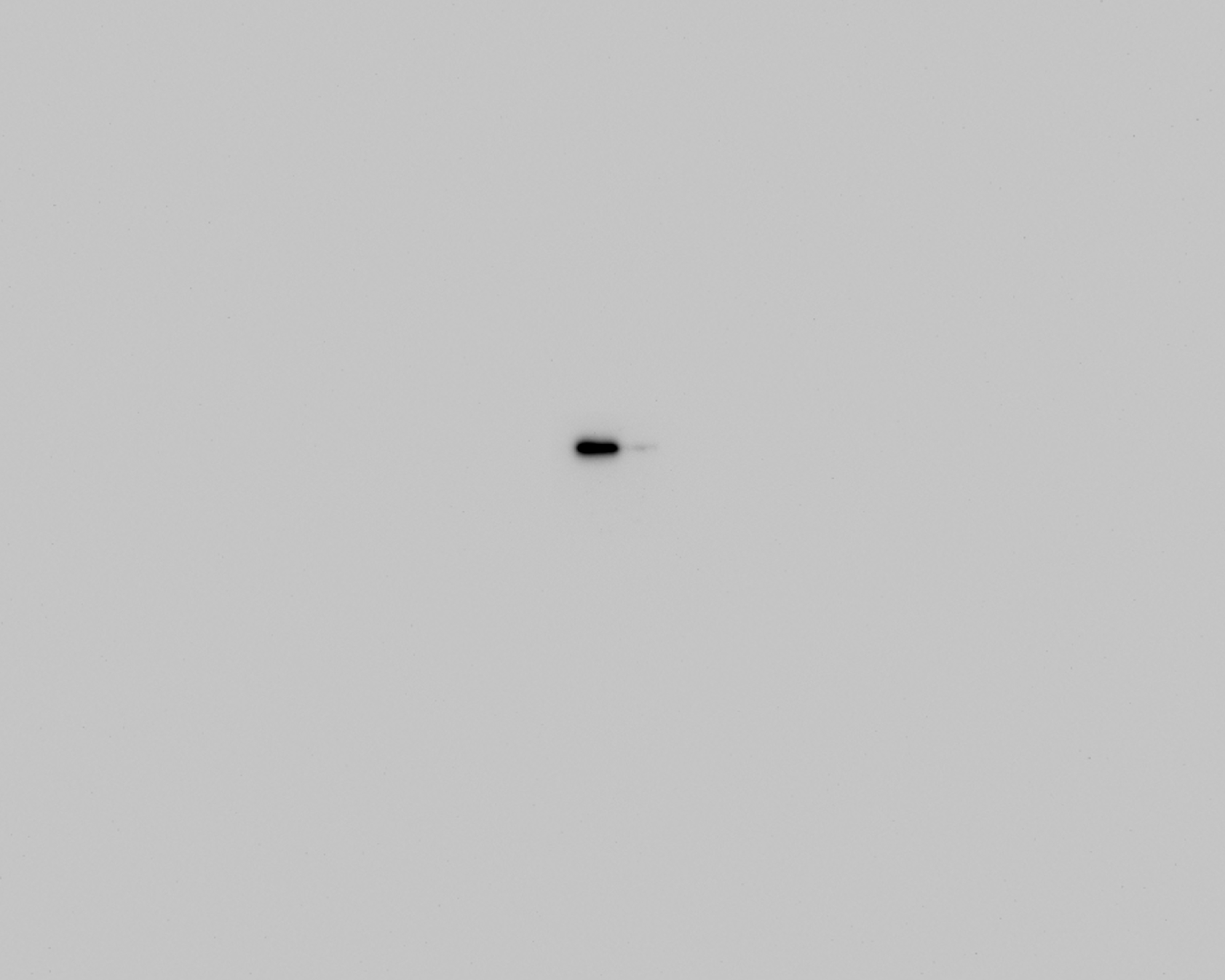

Supplement: Supplementary file 6 — Source data Fig. 4 [file 44319_2026_744_MOESM6_ESM.zip › Figure 1/1H/RCC/Other reps for quantification/2024-12-10 achn,a498 rep4 k36m3(Chemiluminescence).jpg]

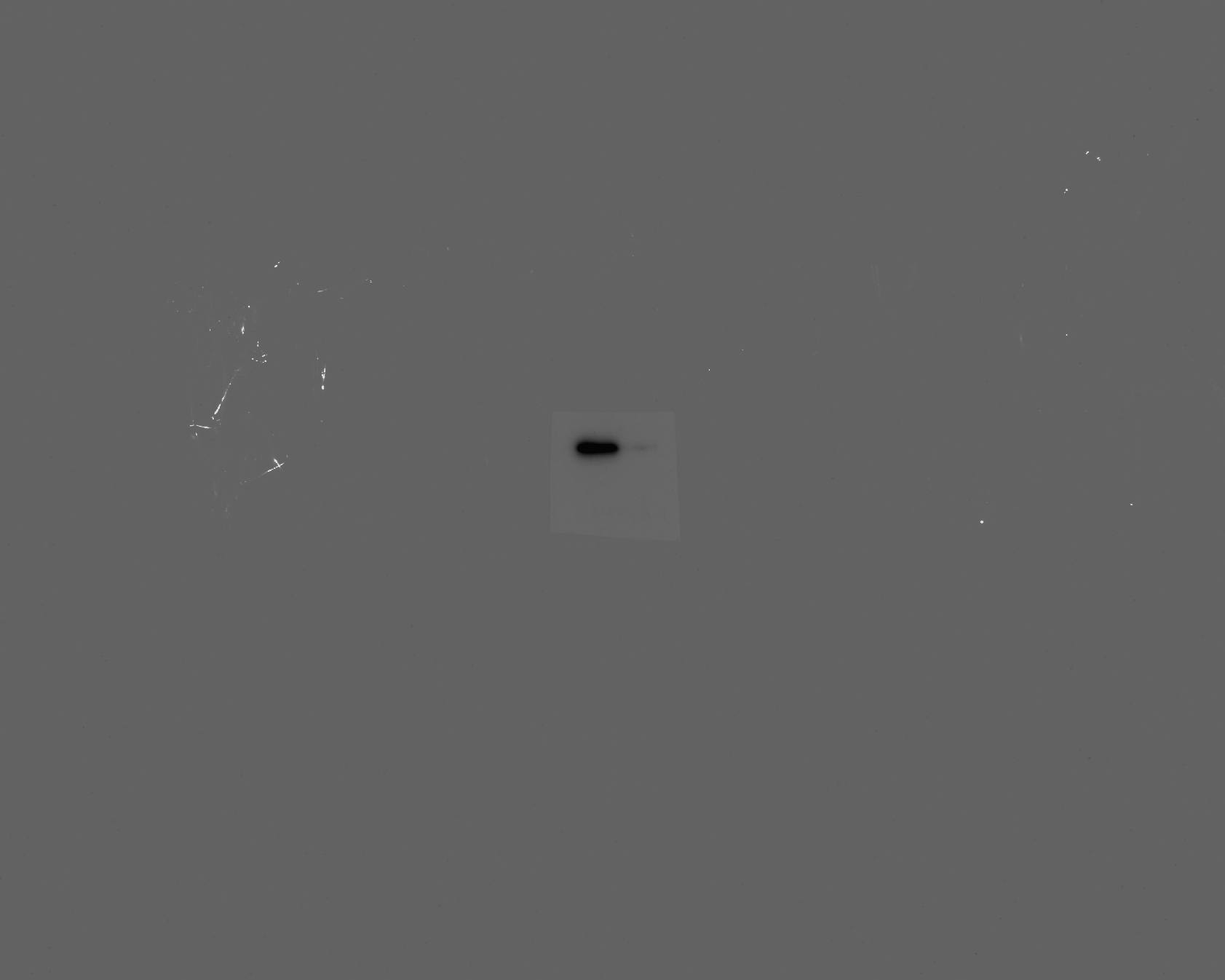

Supplement: Supplementary file 6 — Source data Fig. 4 [file 44319_2026_744_MOESM6_ESM.zip › Figure 1/1H/RCC/Other reps for quantification/2024-12-10 achn,a498 rep4 k36m3(Composite).jpg]

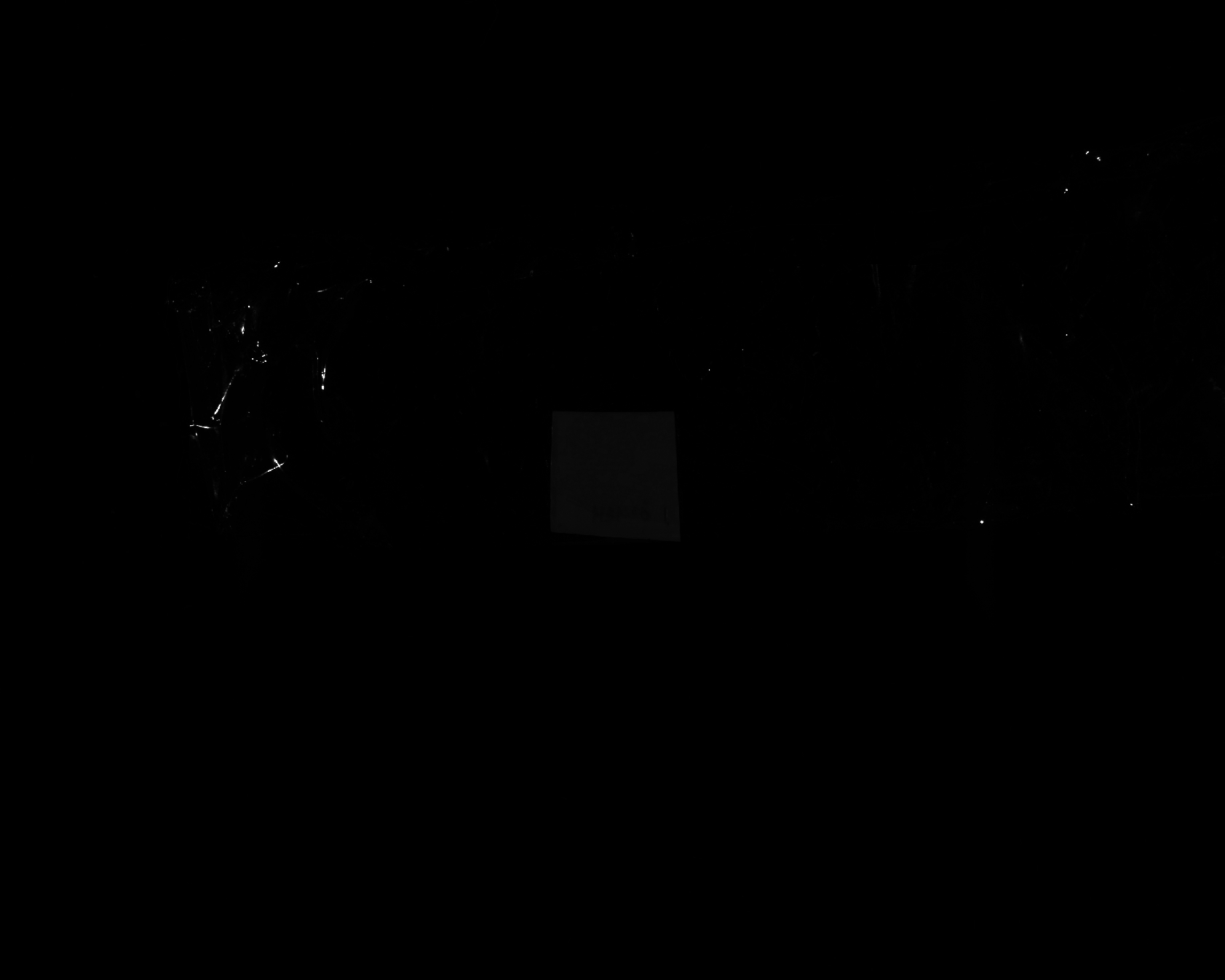

Supplement: Supplementary file 6 — Source data Fig. 4 [file 44319_2026_744_MOESM6_ESM.zip › Figure 1/1H/RCC/Other reps for quantification/2024-12-10 achn,a498 rep4 k36m3(Ponceau S).jpg]

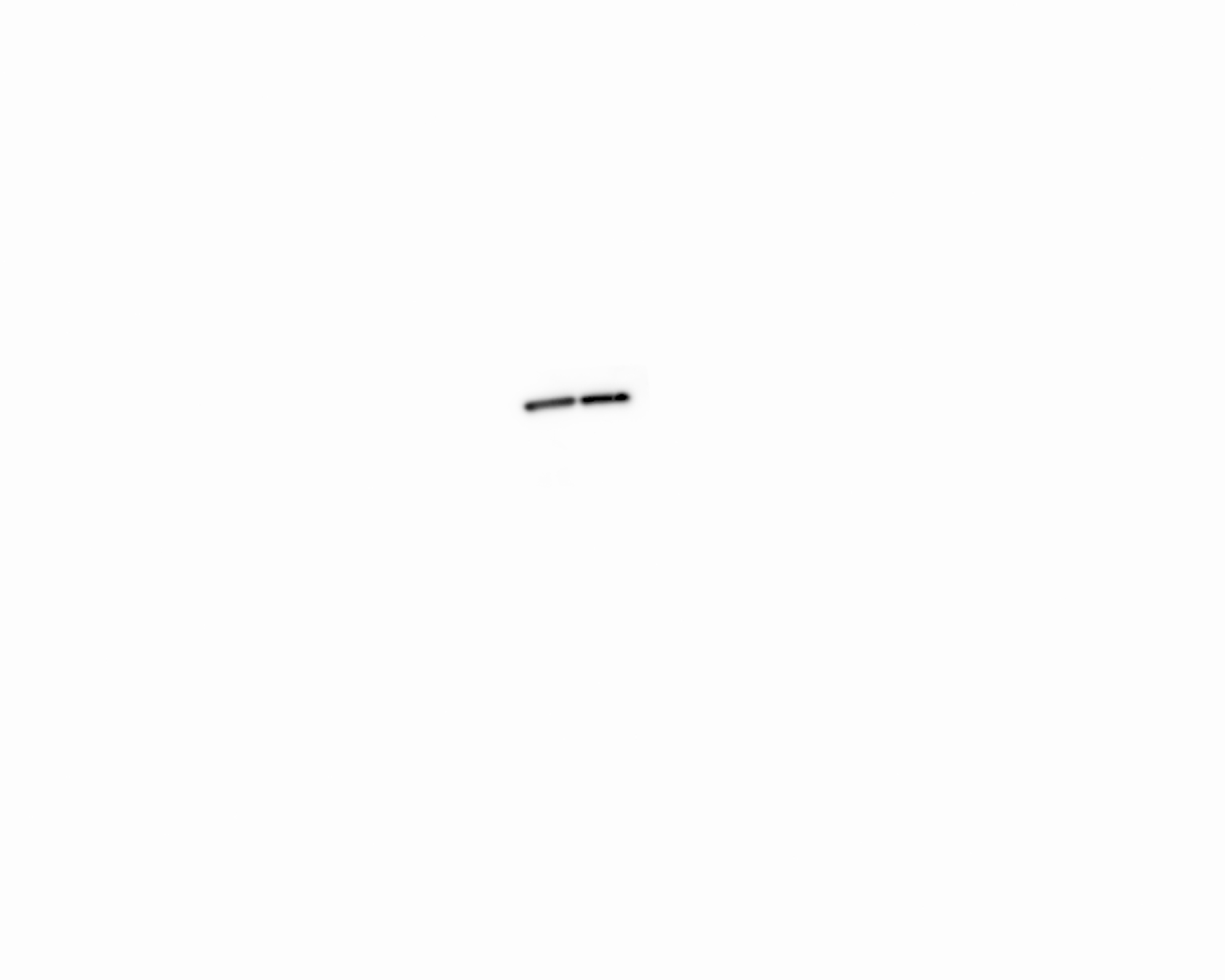

Supplement: Supplementary file 6 — Source data Fig. 4 [file 44319_2026_744_MOESM6_ESM.zip › Figure 1/1H/U2OS/2024-12-03 u2os,ko h3-1(Chemiluminescence).jpg]

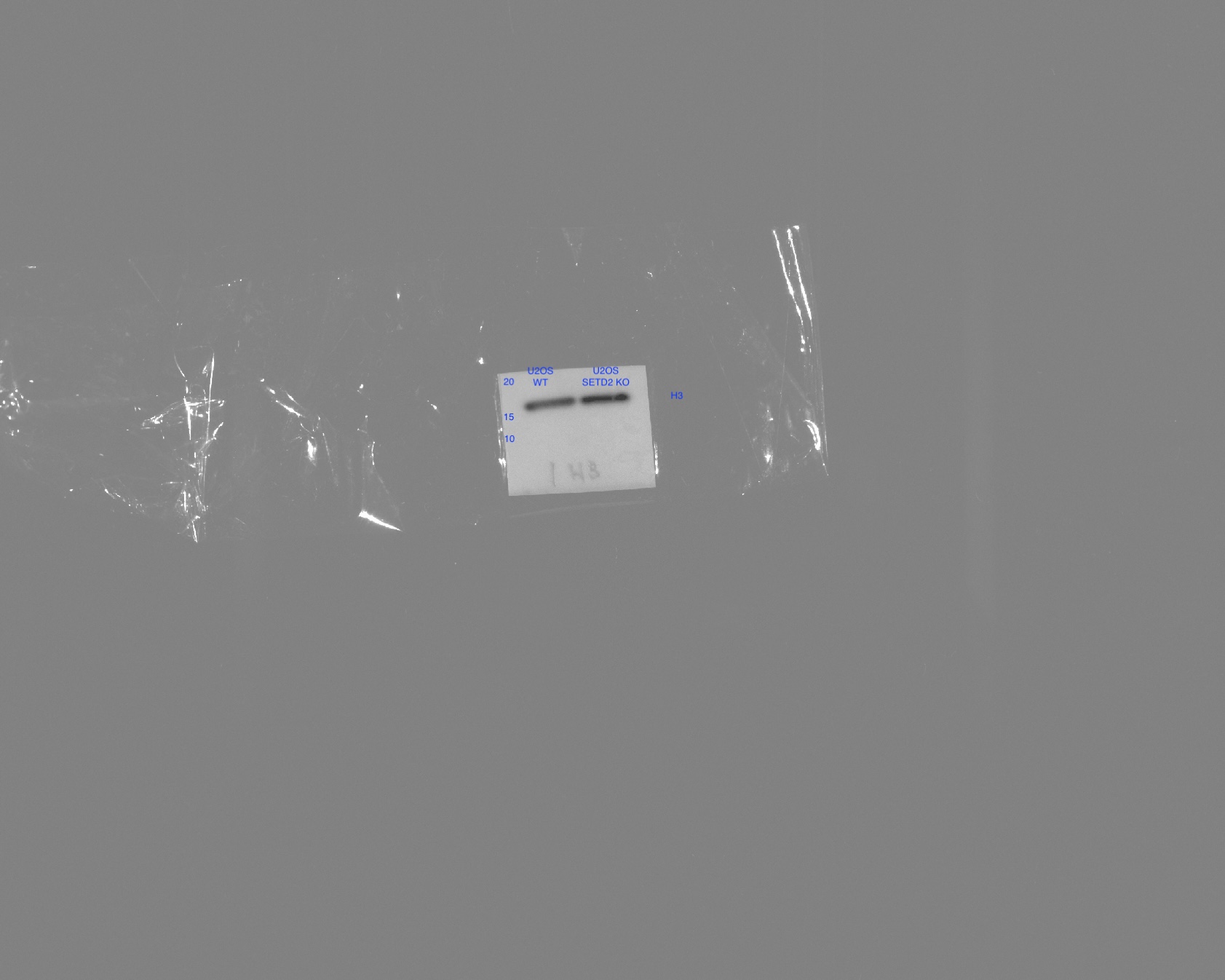

Supplement: Supplementary file 6 — Source data Fig. 4 [file 44319_2026_744_MOESM6_ESM.zip › Figure 1/1H/U2OS/2024-12-03 u2os,ko h3-1(Composite).jpg]

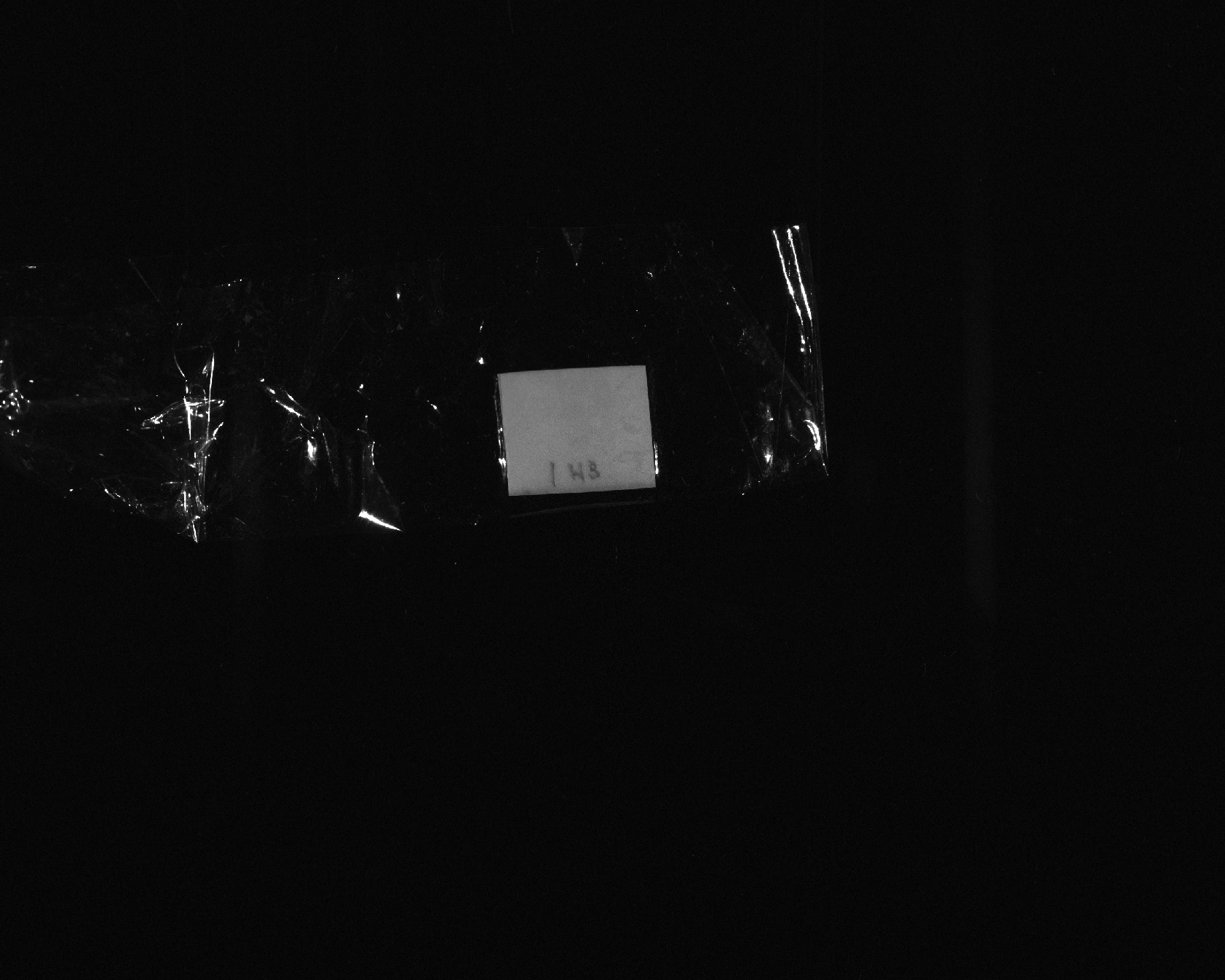

Supplement: Supplementary file 6 — Source data Fig. 4 [file 44319_2026_744_MOESM6_ESM.zip › Figure 1/1H/U2OS/2024-12-03 u2os,ko h3-1(Ponceau S).jpg]

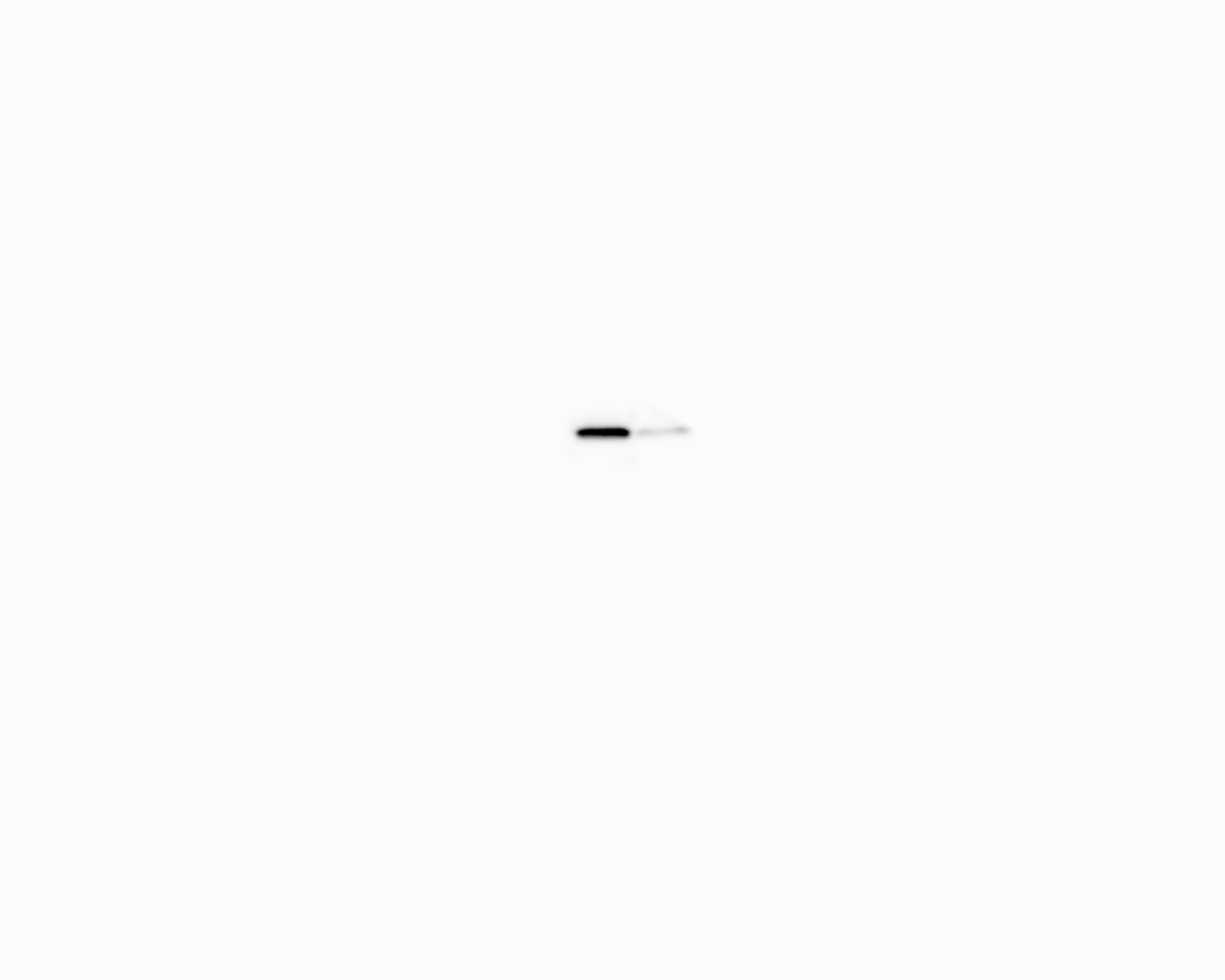

Supplement: Supplementary file 6 — Source data Fig. 4 [file 44319_2026_744_MOESM6_ESM.zip › Figure 1/1H/U2OS/2024-12-03 u2os,ko k36m3(Chemiluminescence).jpg]

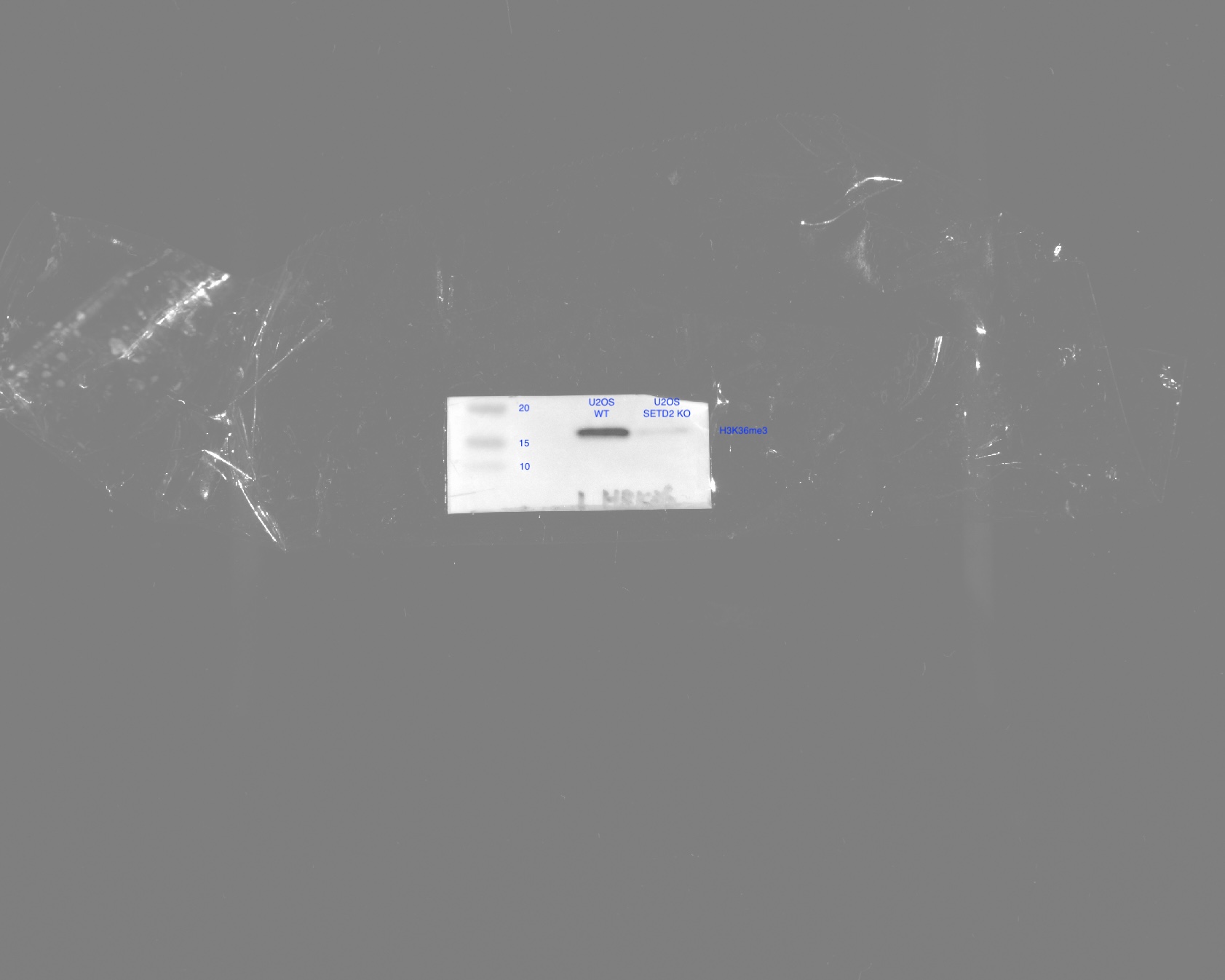

Supplement: Supplementary file 6 — Source data Fig. 4 [file 44319_2026_744_MOESM6_ESM.zip › Figure 1/1H/U2OS/2024-12-03 u2os,ko k36m3(Composite).jpg]

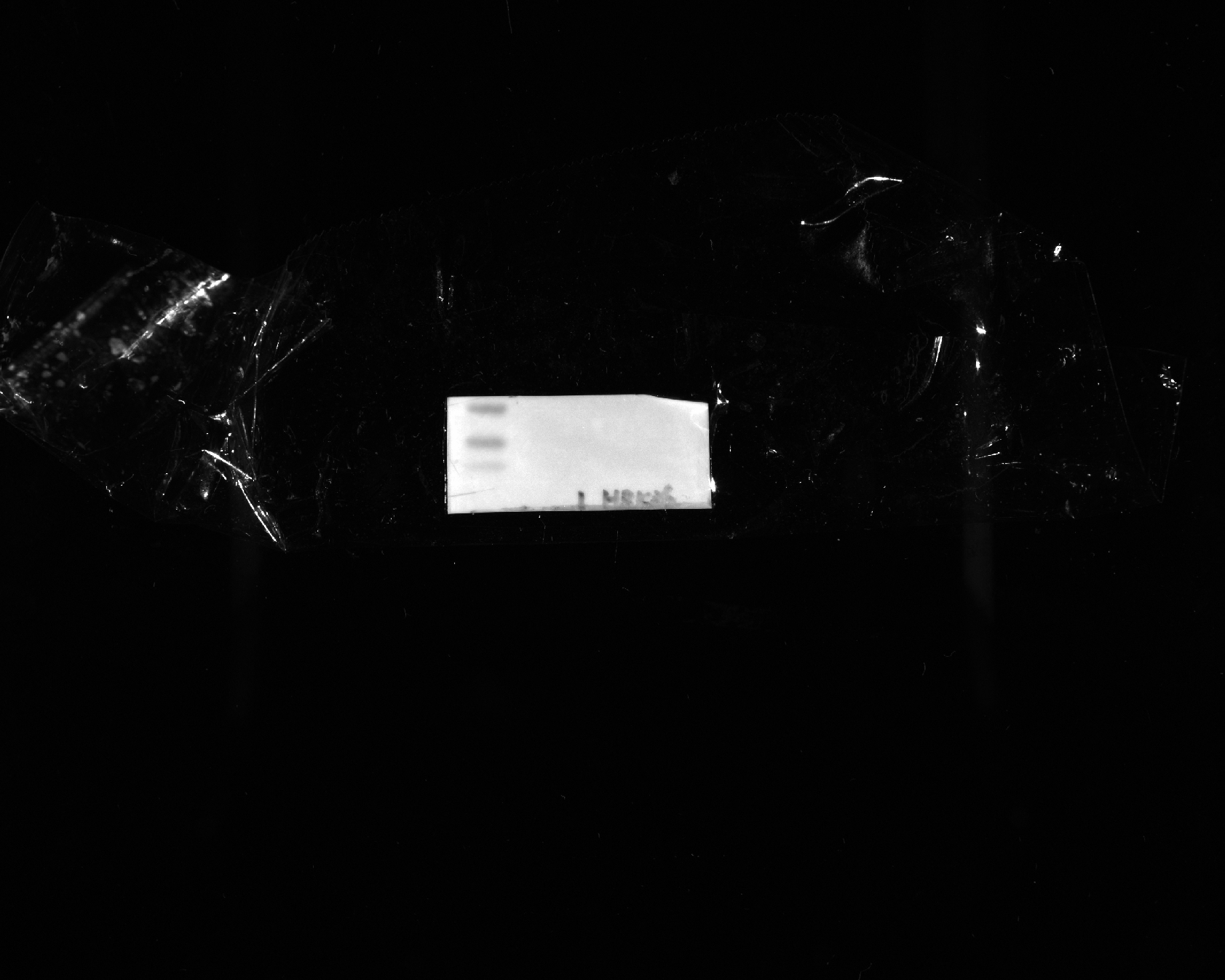

Supplement: Supplementary file 6 — Source data Fig. 4 [file 44319_2026_744_MOESM6_ESM.zip › Figure 1/1H/U2OS/2024-12-03 u2os,ko k36m3(Ponceau S).jpg]

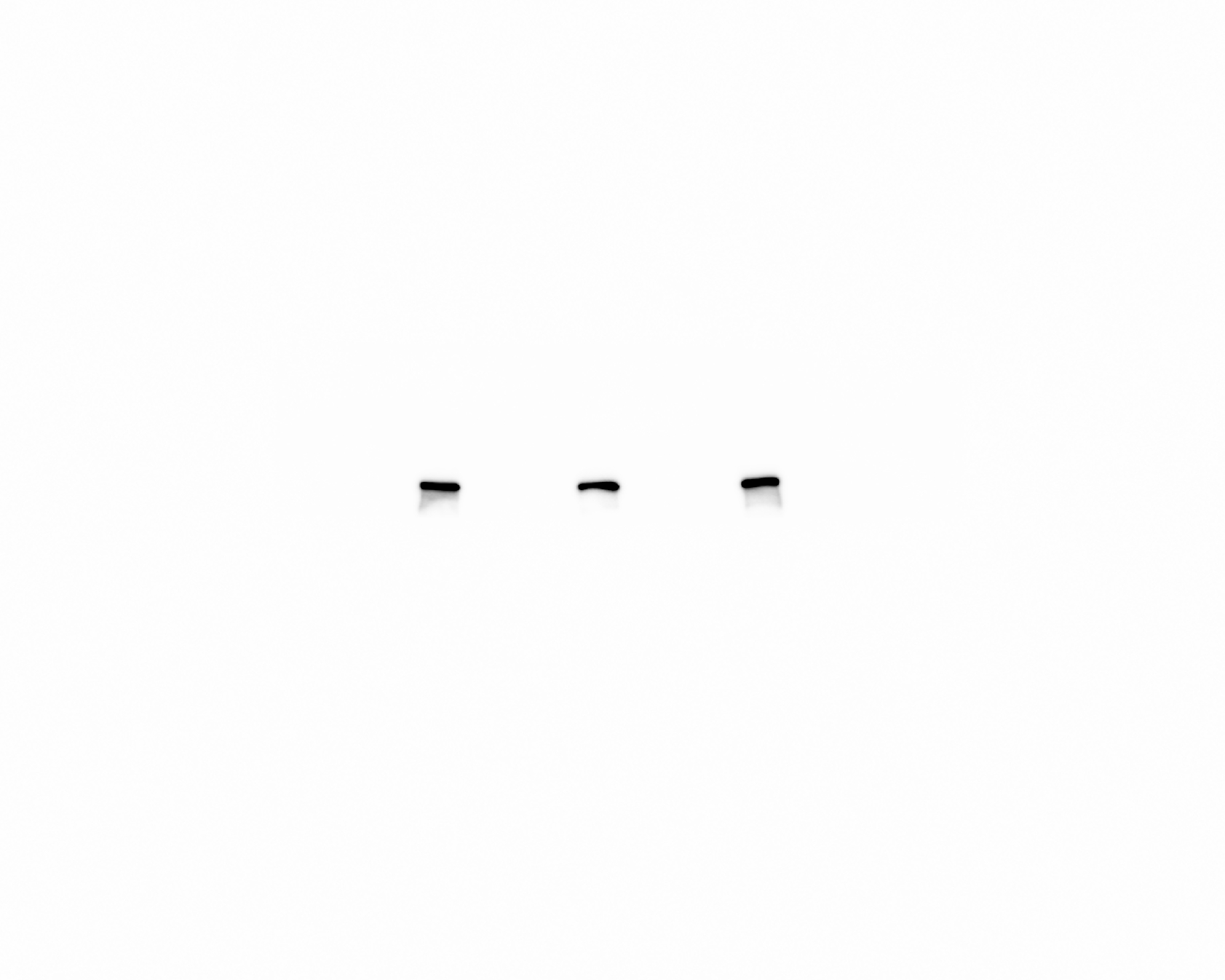

Supplement: Supplementary file 6 — Source data Fig. 4 [file 44319_2026_744_MOESM6_ESM.zip › Figure 1/1H/U2OS/2024-12-03 u2os,ko setd2-1(Chemiluminescence).jpg]

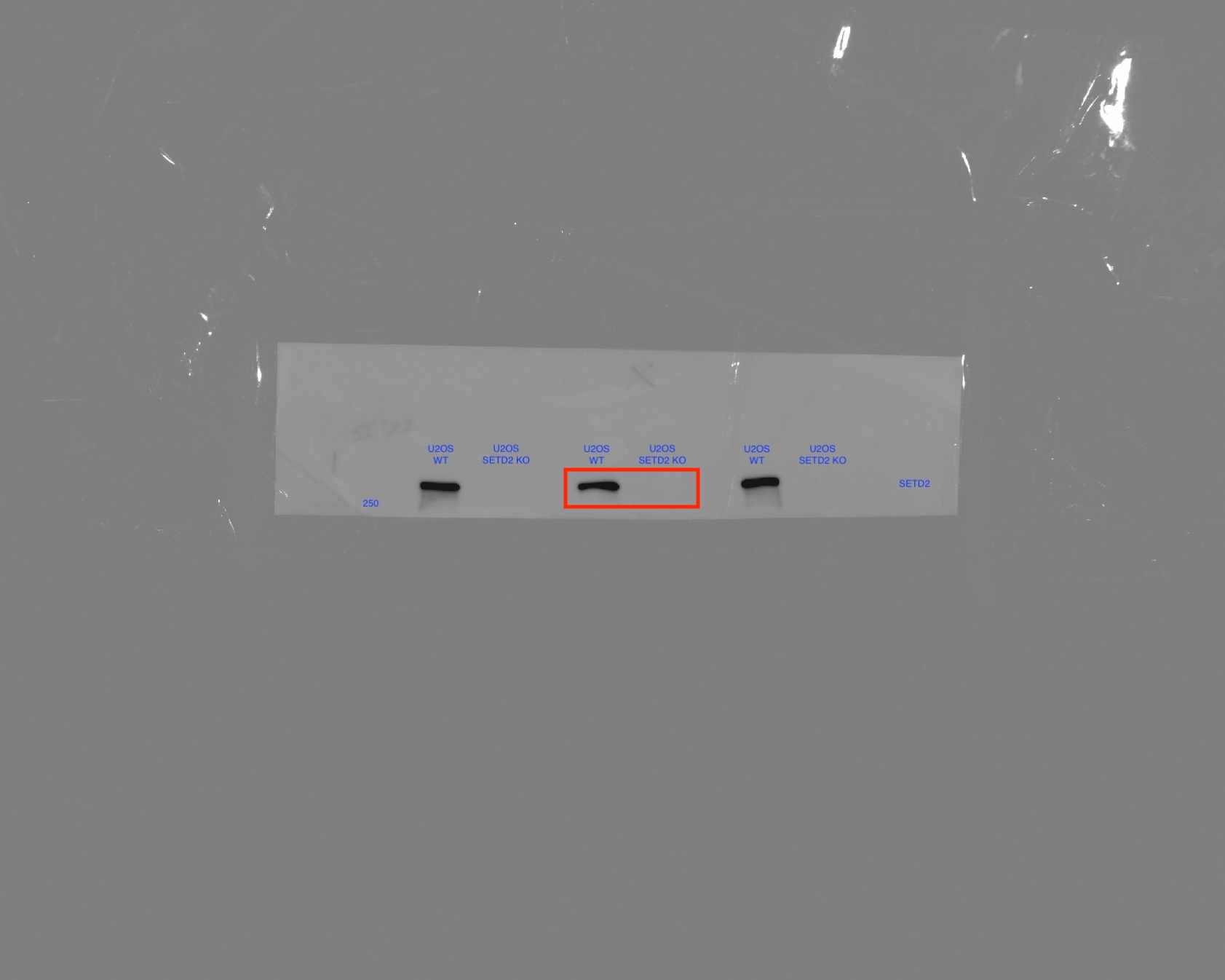

Supplement: Supplementary file 6 — Source data Fig. 4 [file 44319_2026_744_MOESM6_ESM.zip › Figure 1/1H/U2OS/2024-12-03 u2os,ko setd2-1(Composite).jpg]

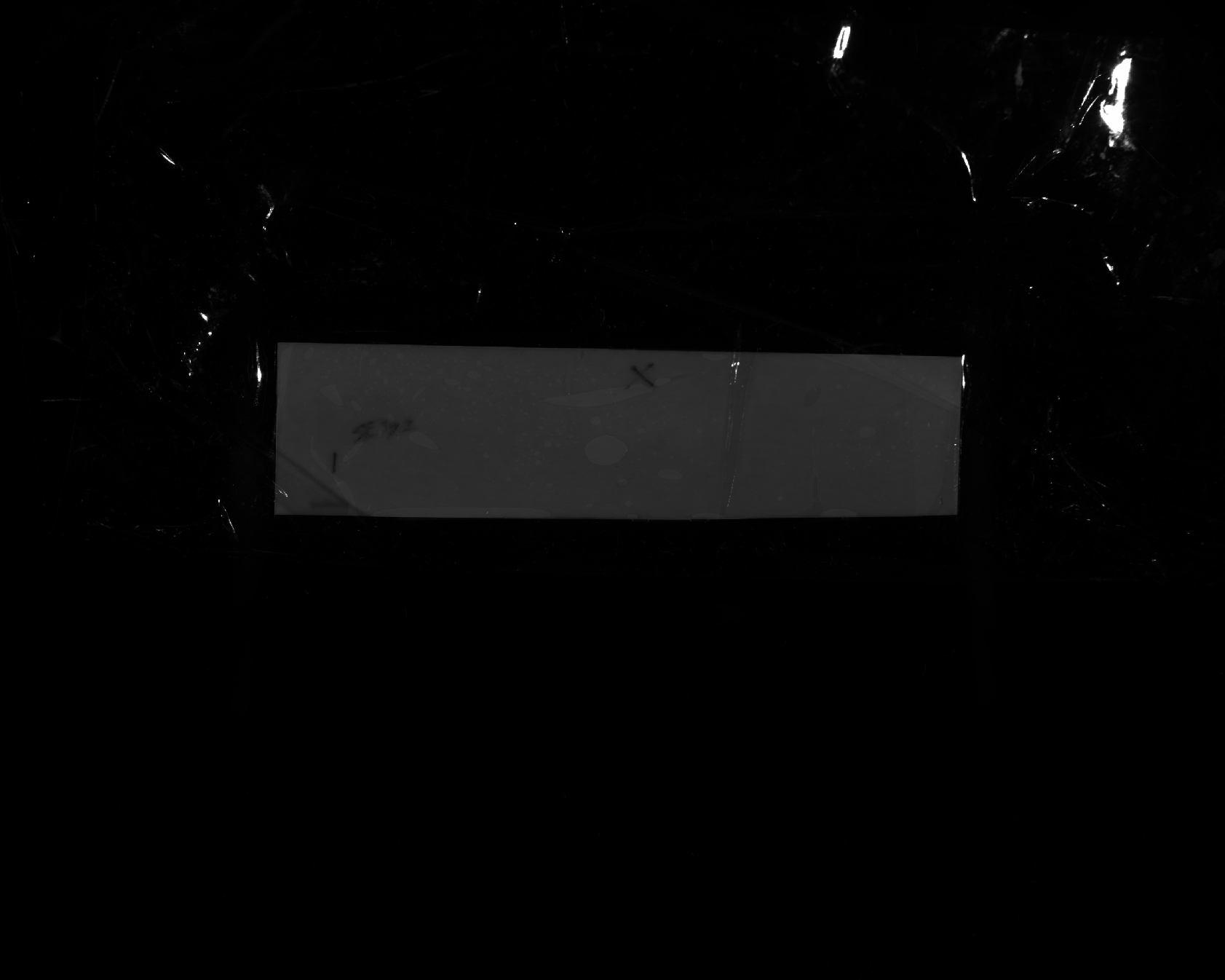

Supplement: Supplementary file 6 — Source data Fig. 4 [file 44319_2026_744_MOESM6_ESM.zip › Figure 1/1H/U2OS/2024-12-03 u2os,ko setd2-1(Ponceau S).jpg]

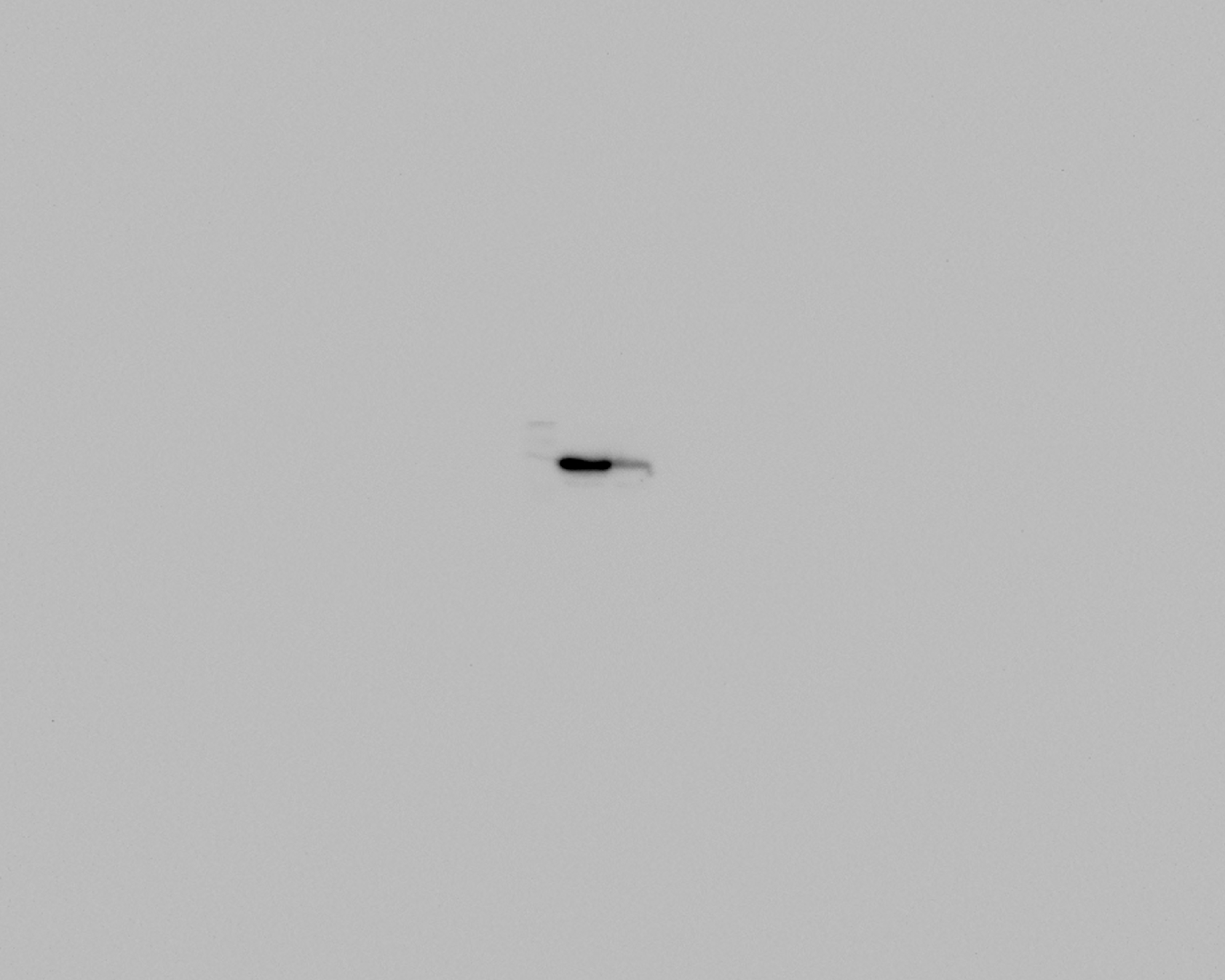

Supplement: Supplementary file 7 — Figure EV4 Source Data [file 44319_2026_744_MOESM7_ESM.zip › Figure 4/4A/day3/2025-01-09 h3k36m3 d3(Chemiluminescence).jpg]

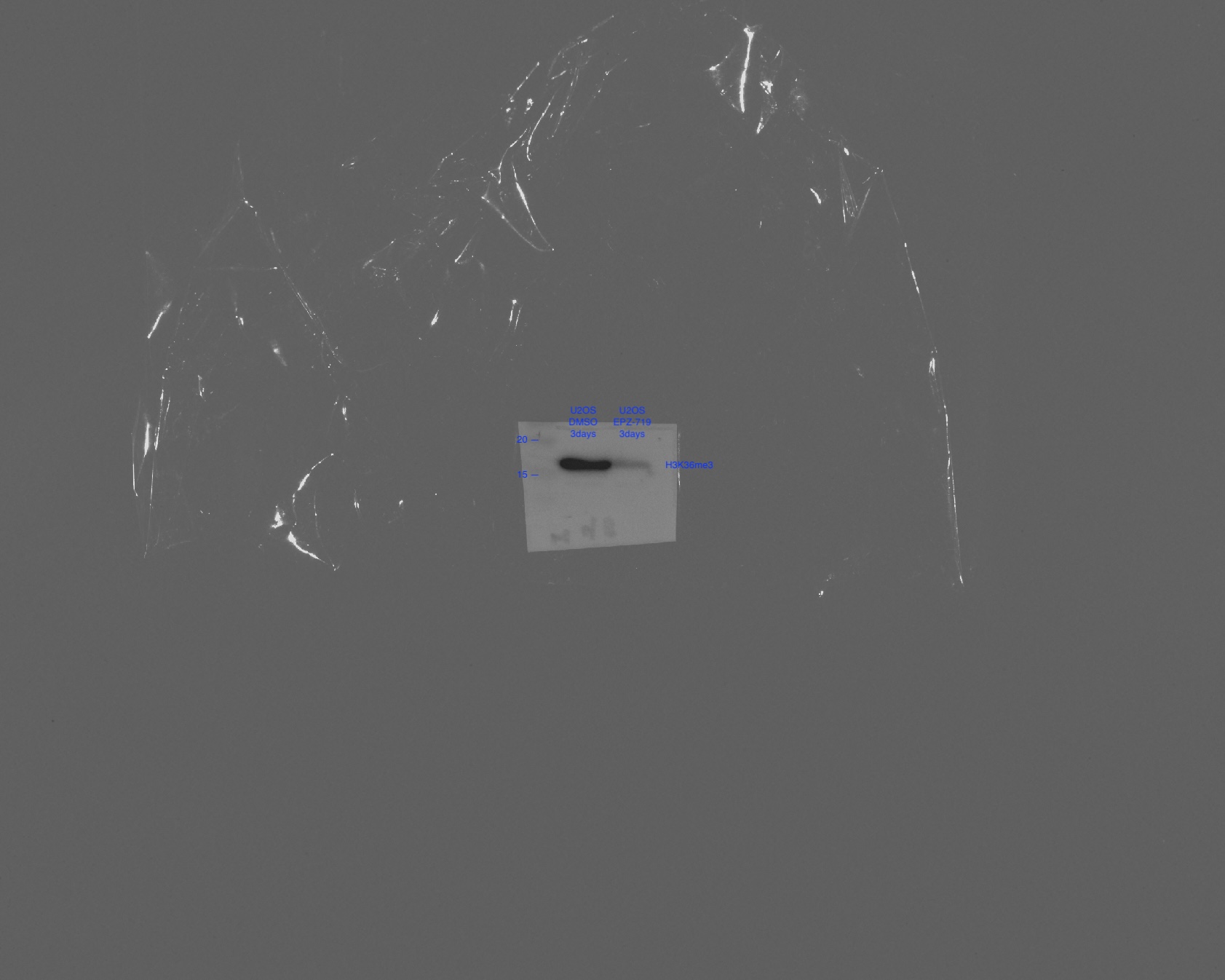

Supplement: Supplementary file 7 — Figure EV4 Source Data [file 44319_2026_744_MOESM7_ESM.zip › Figure 4/4A/day3/2025-01-09 h3k36m3 d3(Composite).jpg]

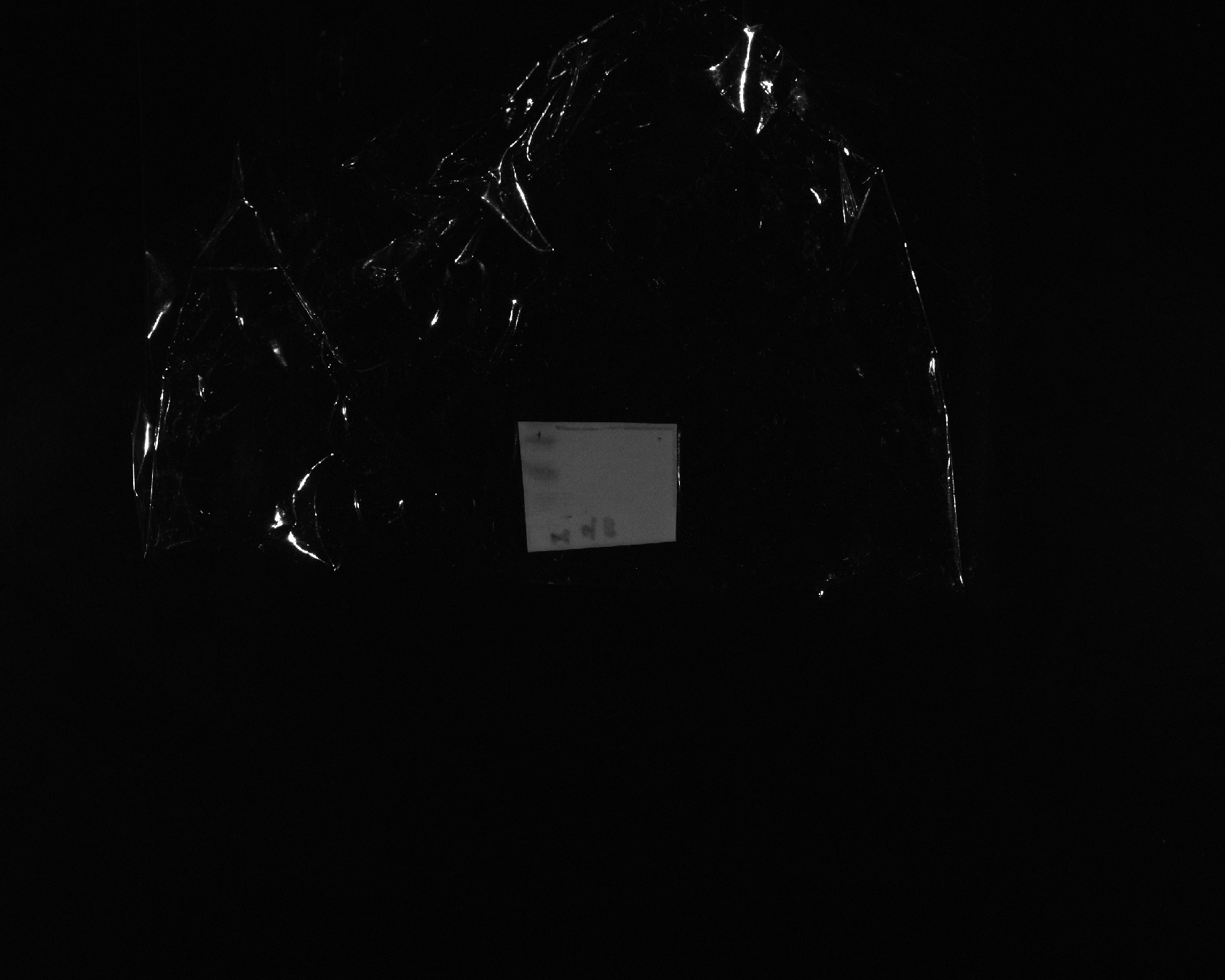

Supplement: Supplementary file 7 — Figure EV4 Source Data [file 44319_2026_744_MOESM7_ESM.zip › Figure 4/4A/day3/2025-01-09 h3k36m3 d3(Ponceau S).jpg]

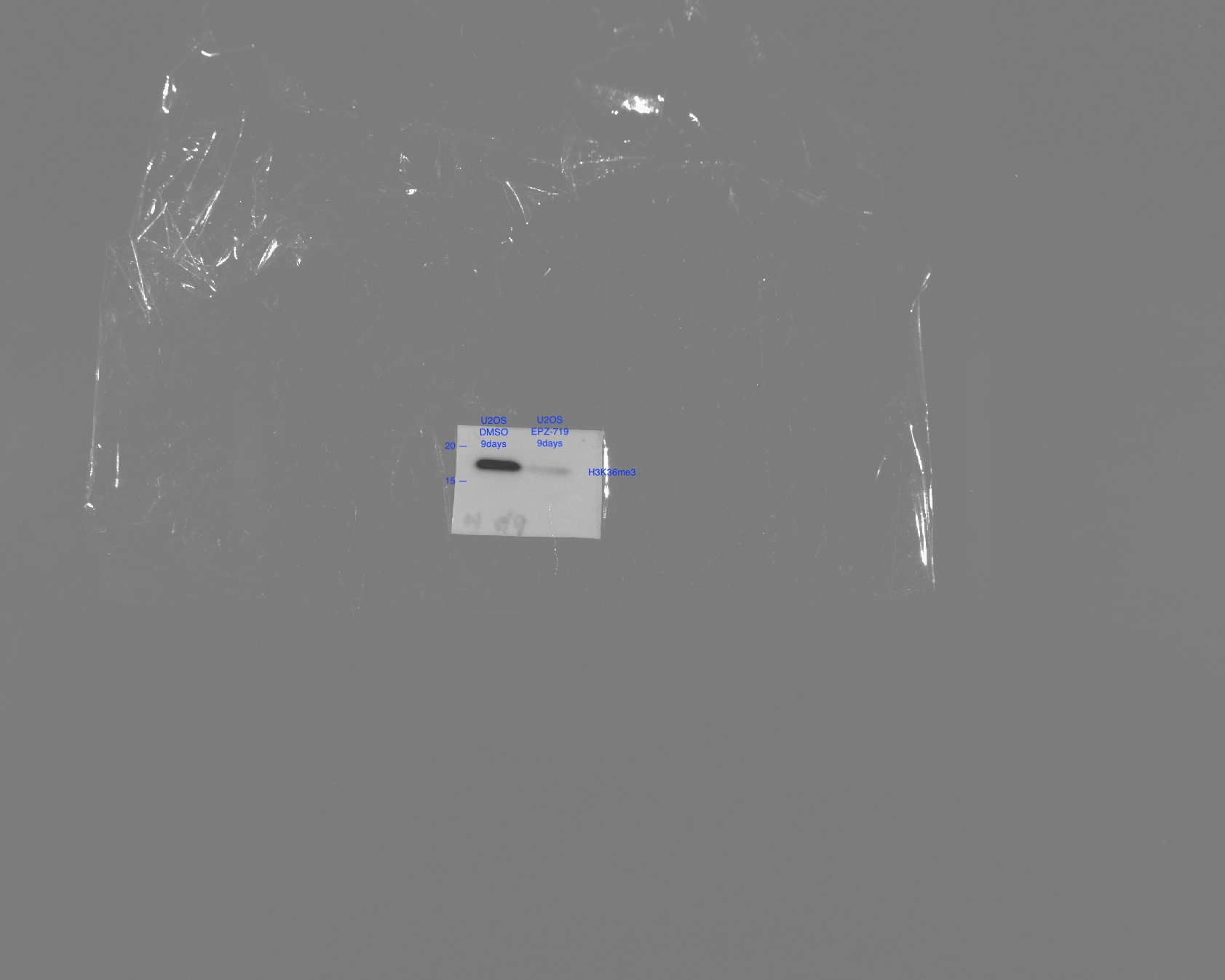

Supplement: Supplementary file 7 — Figure EV4 Source Data [file 44319_2026_744_MOESM7_ESM.zip › Figure 4/4A/day3/2025-01-09 h3k36m3 d9(Composite).jpg]

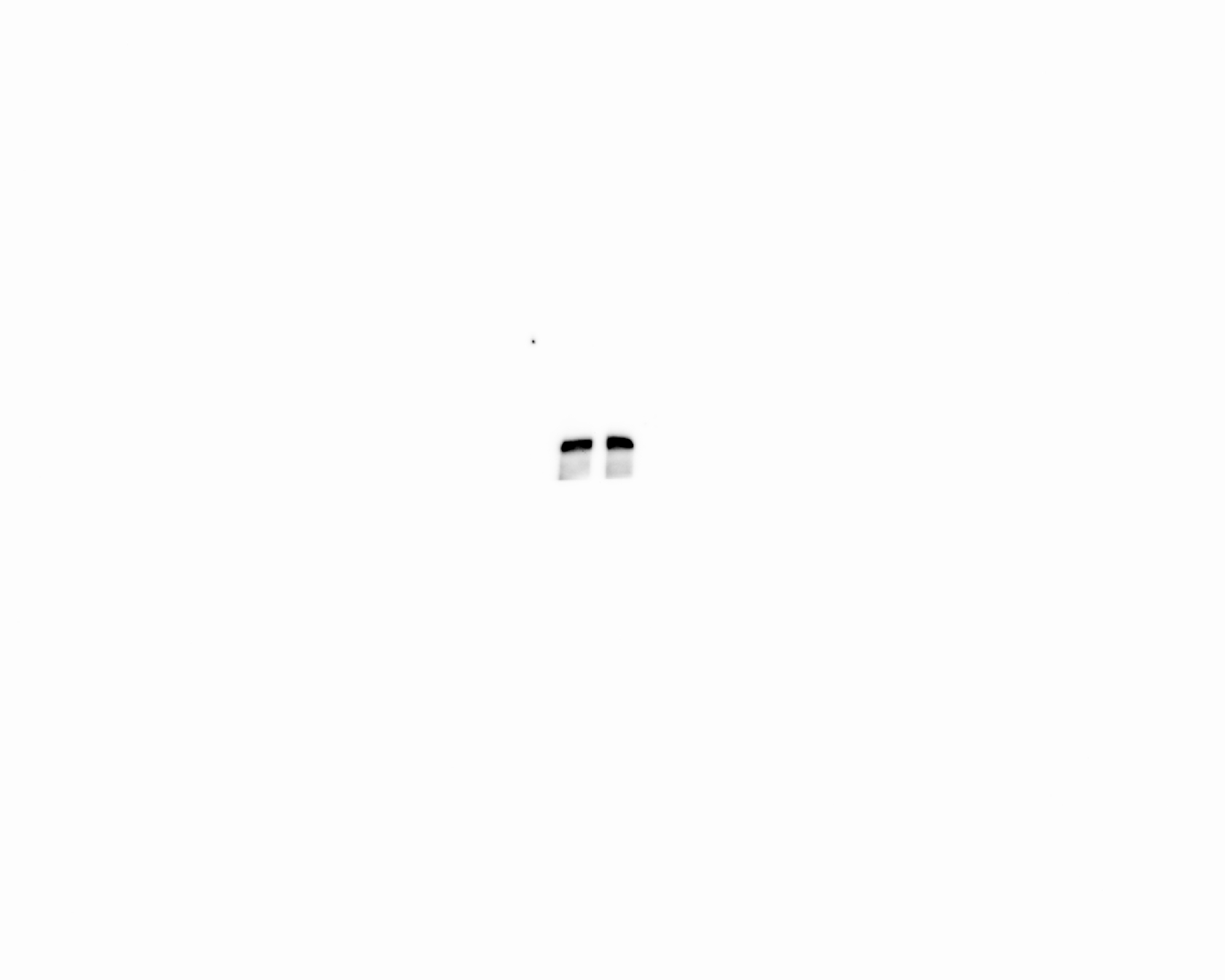

Supplement: Supplementary file 7 — Figure EV4 Source Data [file 44319_2026_744_MOESM7_ESM.zip › Figure 4/4A/day3/2025-01-09 setd2 d3(Chemiluminescence).jpg]

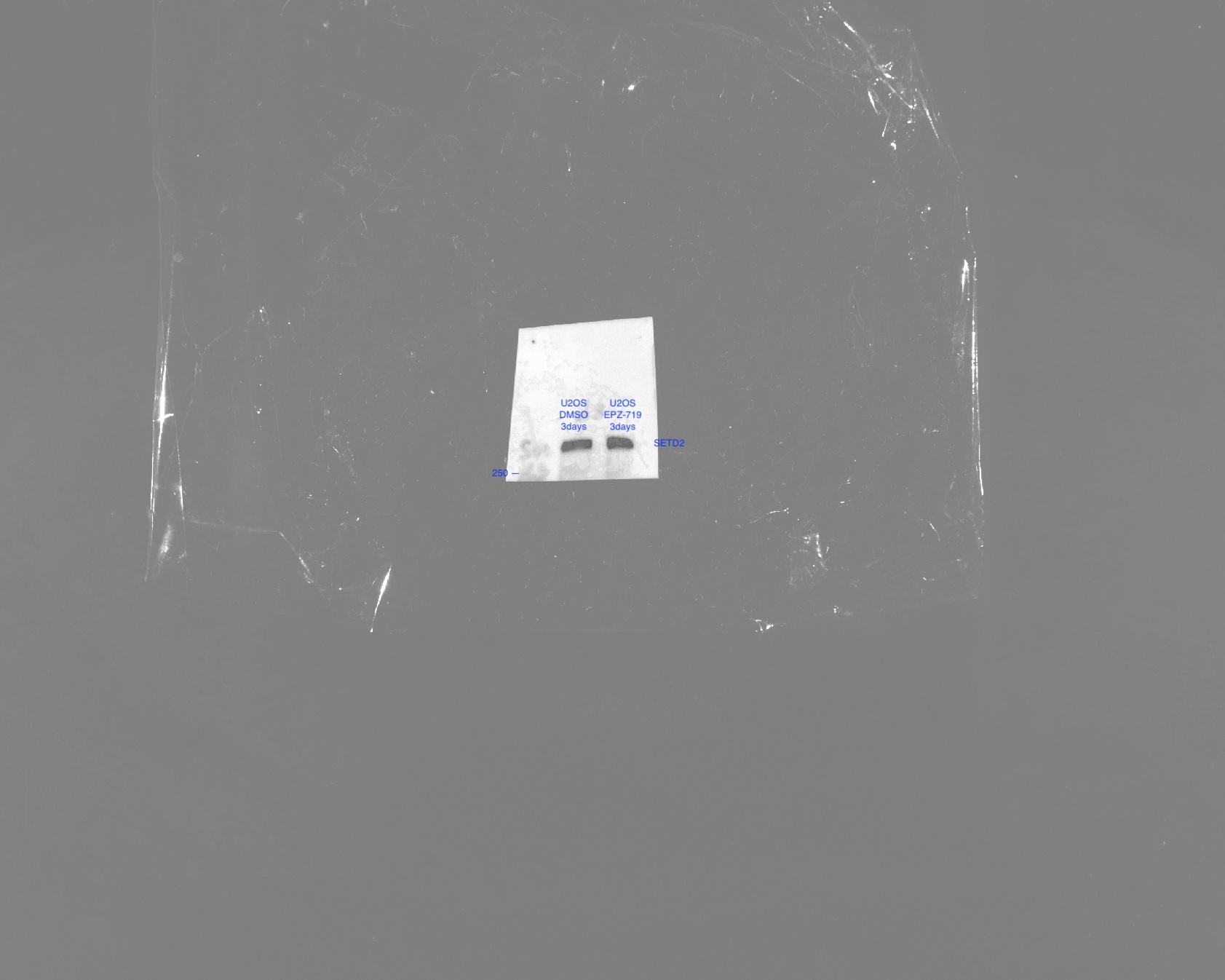

Supplement: Supplementary file 7 — Figure EV4 Source Data [file 44319_2026_744_MOESM7_ESM.zip › Figure 4/4A/day3/2025-01-09 setd2 d3(Composite).jpg]

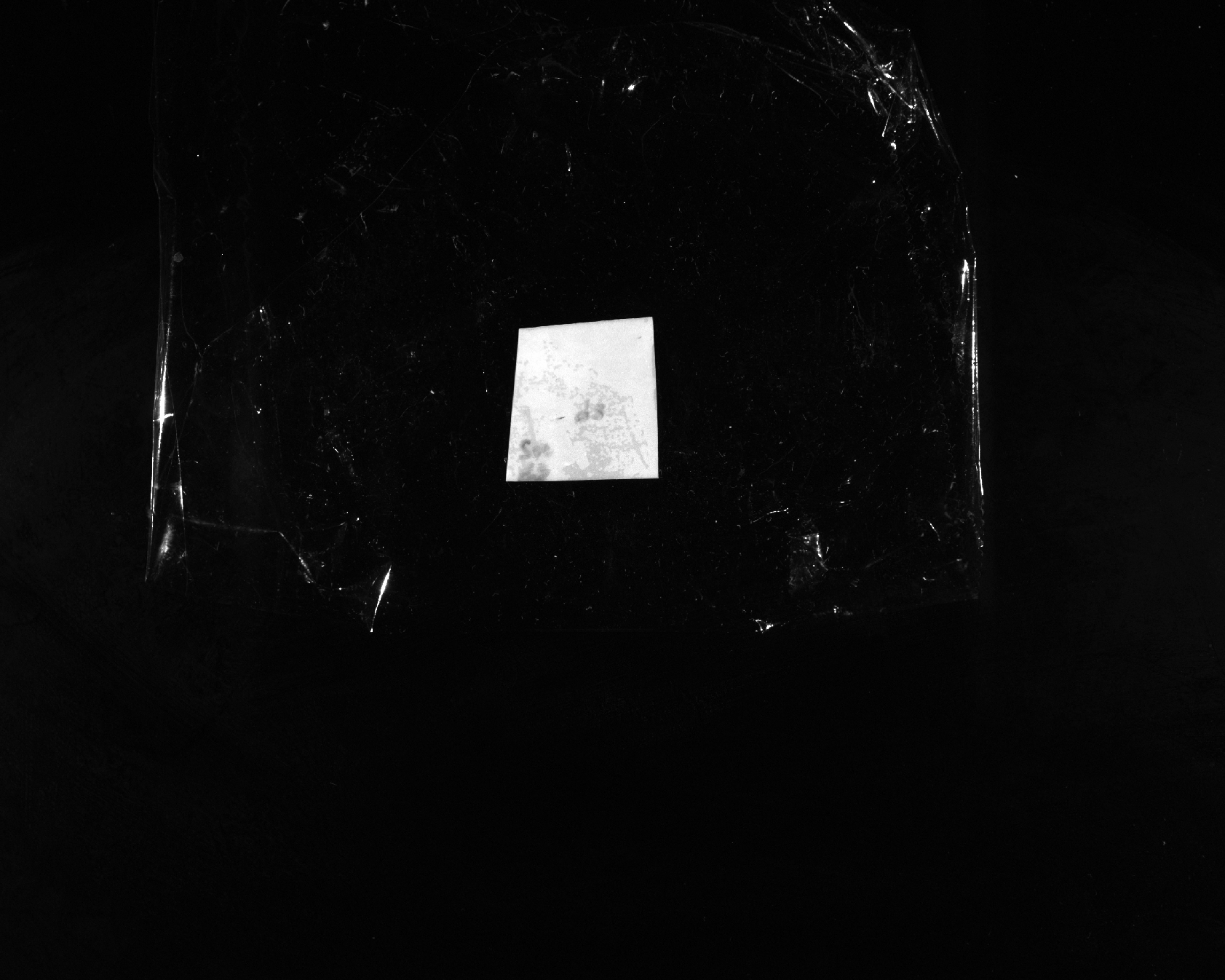

Supplement: Supplementary file 7 — Figure EV4 Source Data [file 44319_2026_744_MOESM7_ESM.zip › Figure 4/4A/day3/2025-01-09 setd2 d3(Ponceau S).jpg]

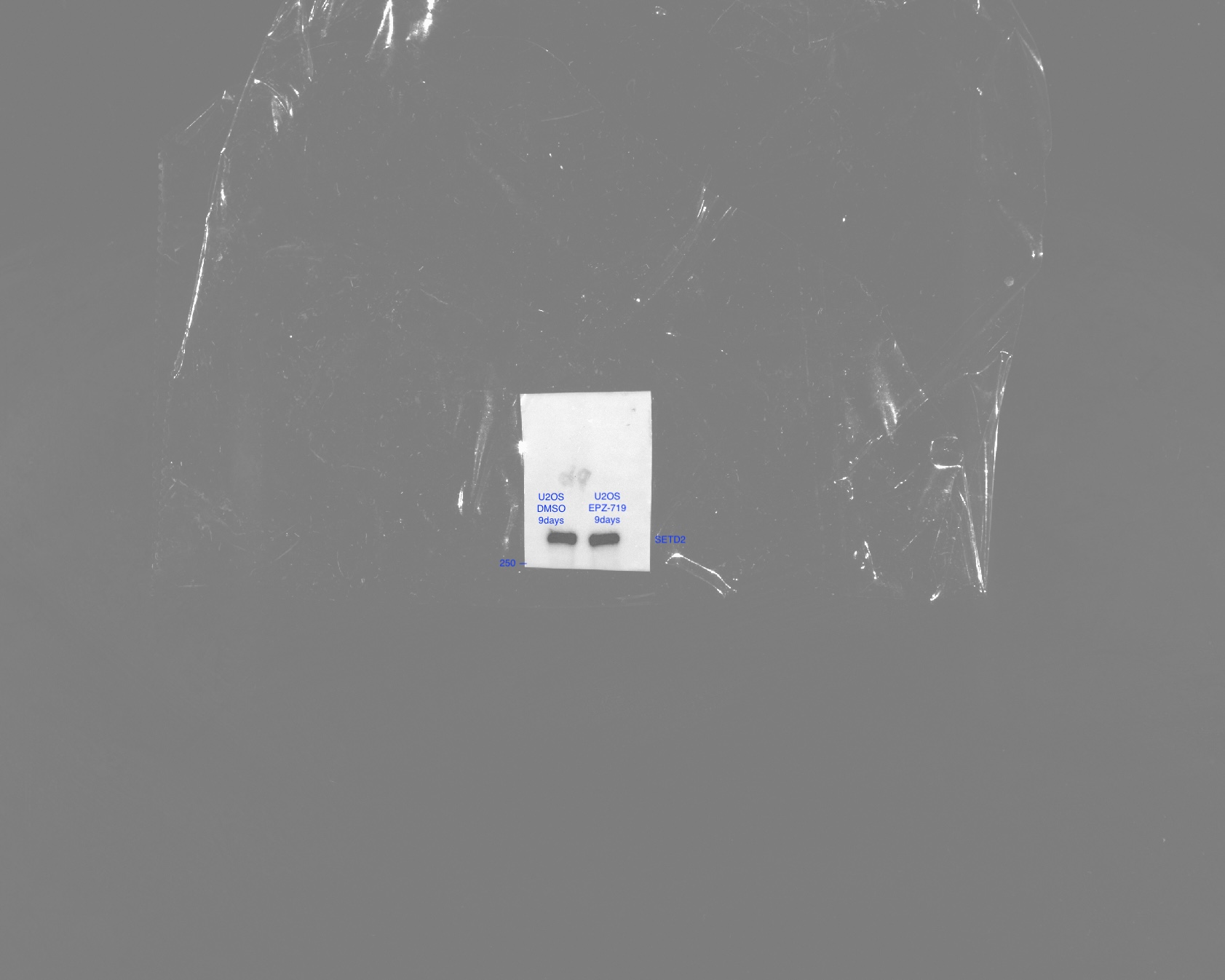

Supplement: Supplementary file 7 — Figure EV4 Source Data [file 44319_2026_744_MOESM7_ESM.zip › Figure 4/4A/day3/2025-01-09 setd2 d9(Composite).jpg]

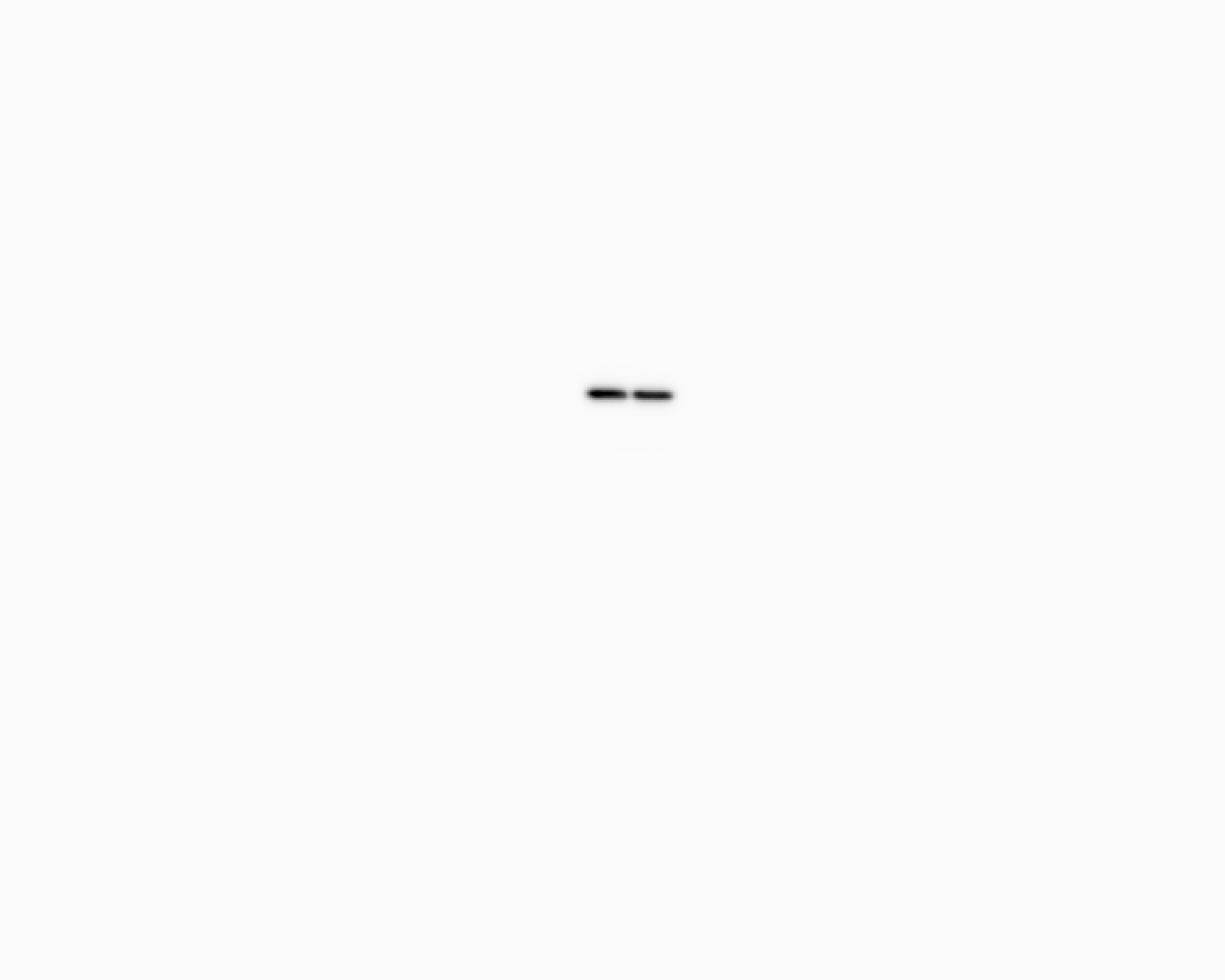

Supplement: Supplementary file 7 — Figure EV4 Source Data [file 44319_2026_744_MOESM7_ESM.zip › Figure 4/4A/day3/2025-01-21 h3 d3(Chemiluminescence).jpg]

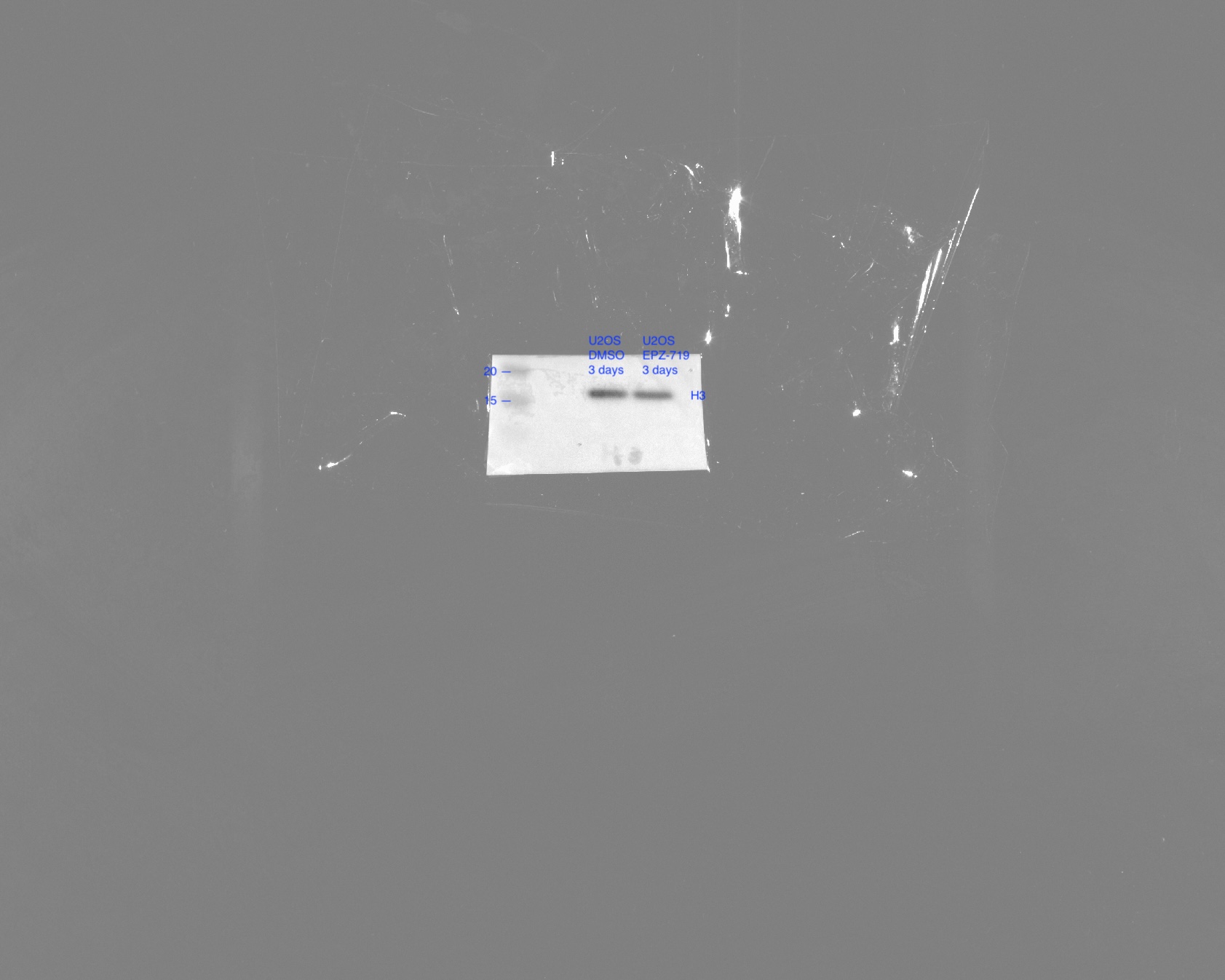

Supplement: Supplementary file 7 — Figure EV4 Source Data [file 44319_2026_744_MOESM7_ESM.zip › Figure 4/4A/day3/2025-01-21 h3 d3(Composite).jpg]

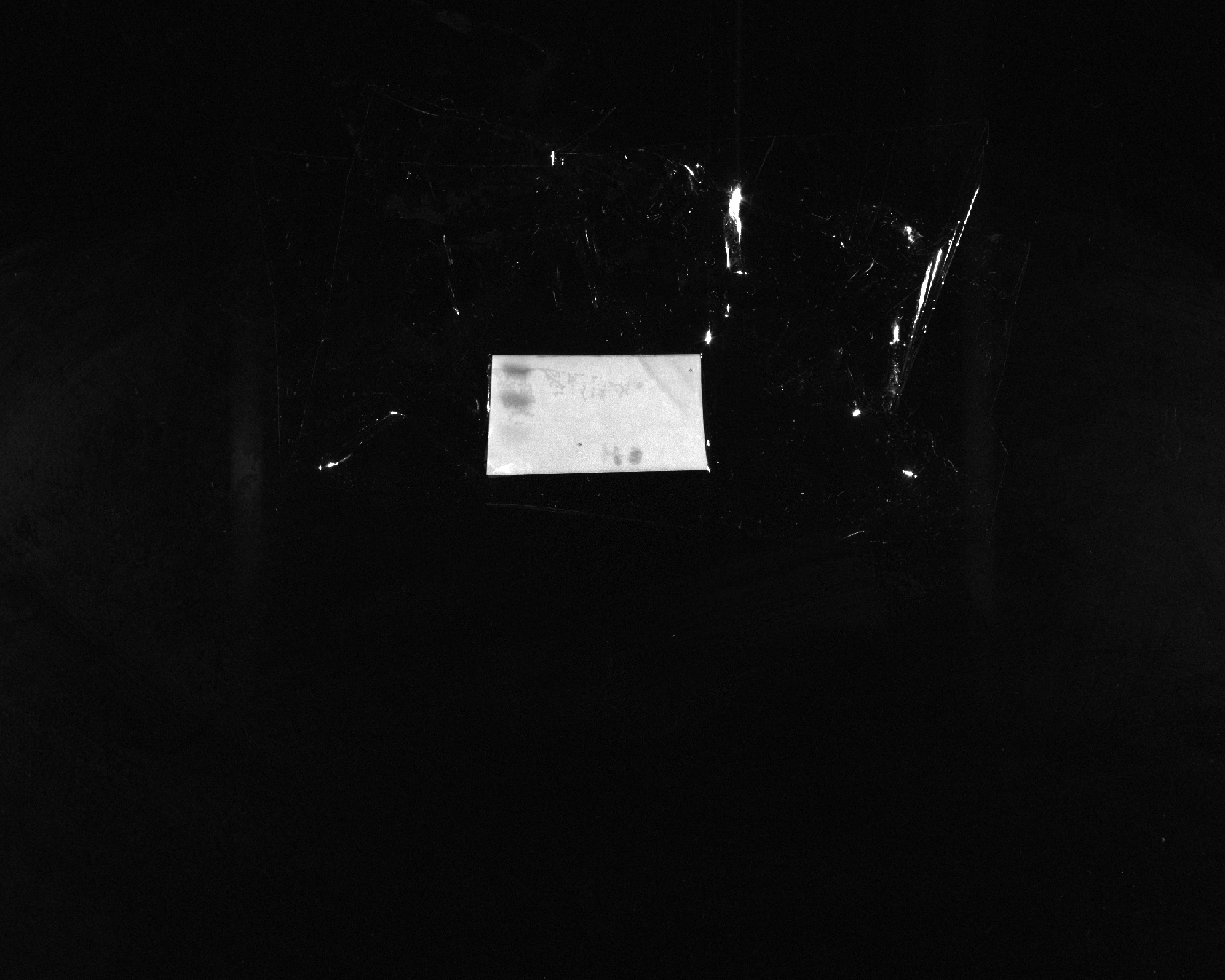

Supplement: Supplementary file 7 — Figure EV4 Source Data [file 44319_2026_744_MOESM7_ESM.zip › Figure 4/4A/day3/2025-01-21 h3 d3(Ponceau S).jpg]

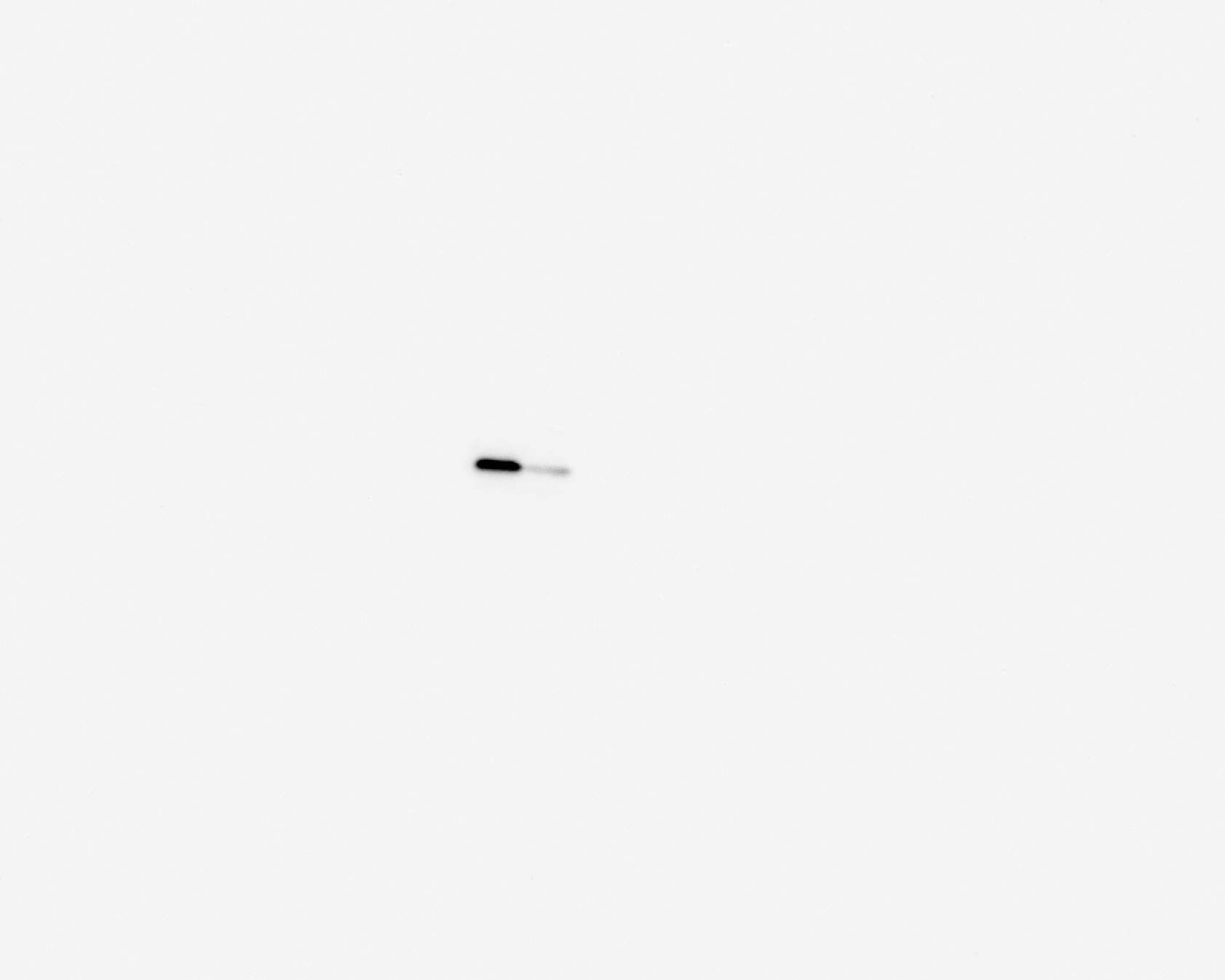

Supplement: Supplementary file 7 — Figure EV4 Source Data [file 44319_2026_744_MOESM7_ESM.zip › Figure 4/4A/day9/2025-01-09 h3k36m3 d9(Chemiluminescence).jpg]

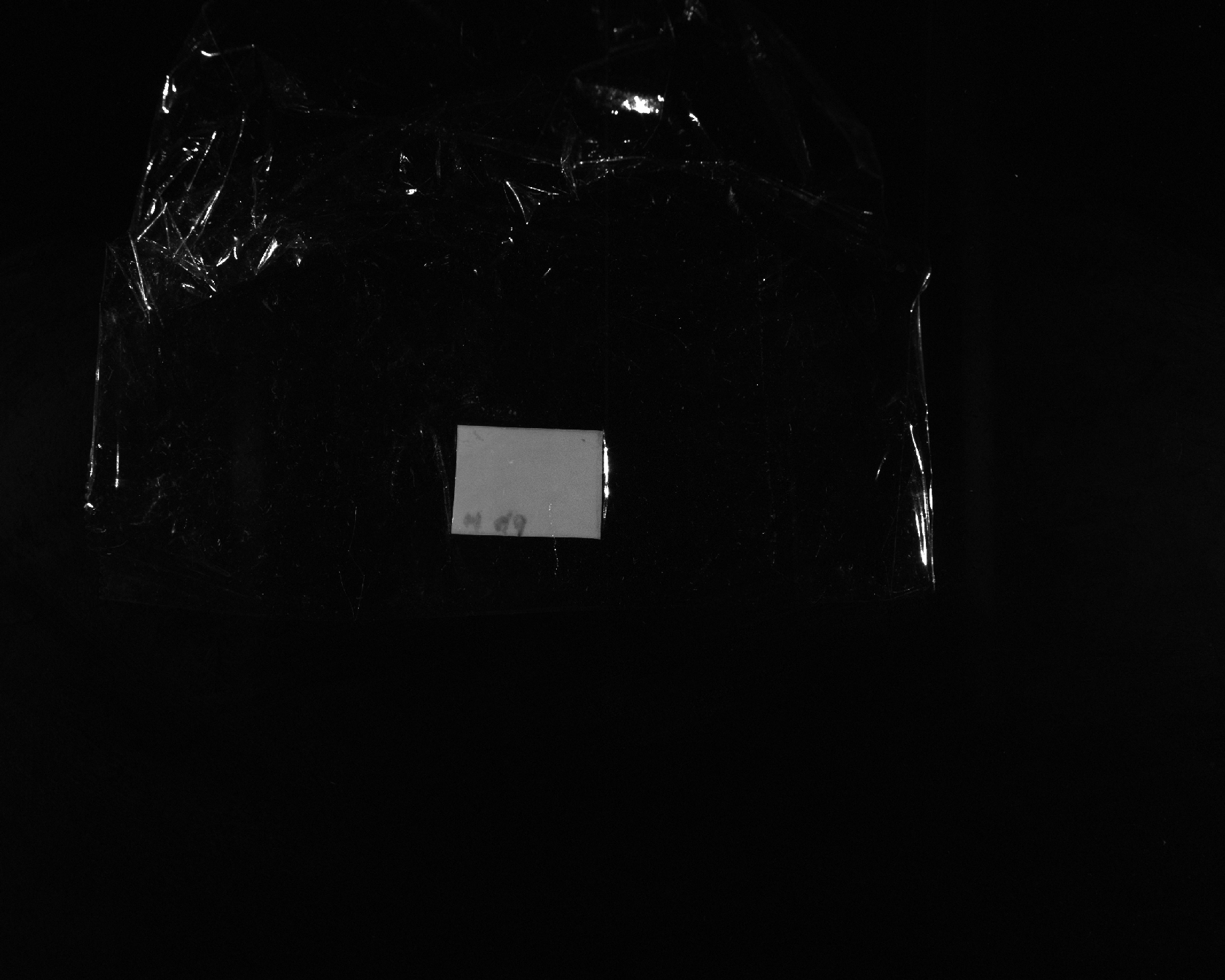

Supplement: Supplementary file 7 — Figure EV4 Source Data [file 44319_2026_744_MOESM7_ESM.zip › Figure 4/4A/day9/2025-01-09 h3k36m3 d9(Ponceau S).jpg]

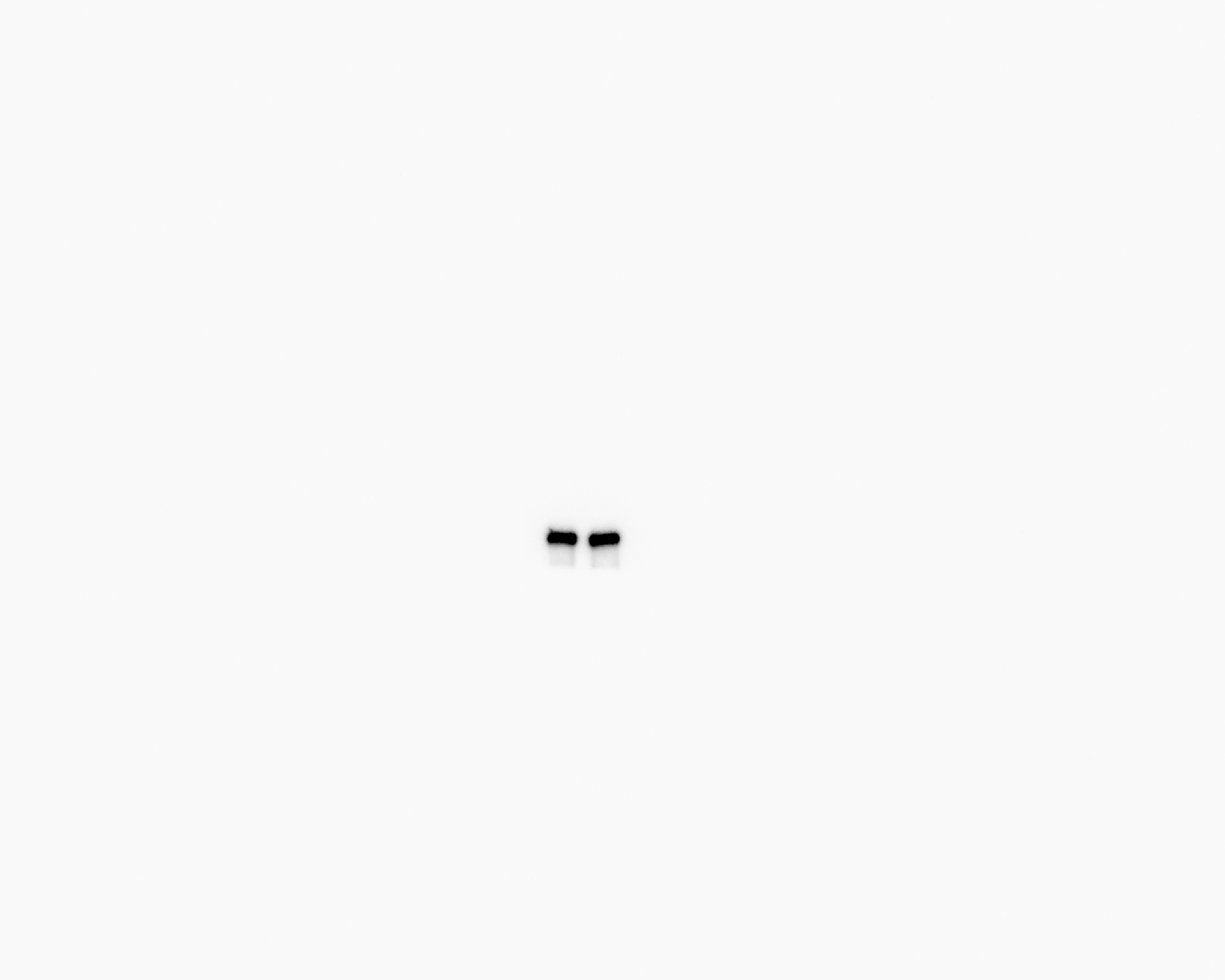

Supplement: Supplementary file 7 — Figure EV4 Source Data [file 44319_2026_744_MOESM7_ESM.zip › Figure 4/4A/day9/2025-01-09 setd2 d9(Chemiluminescence).jpg]

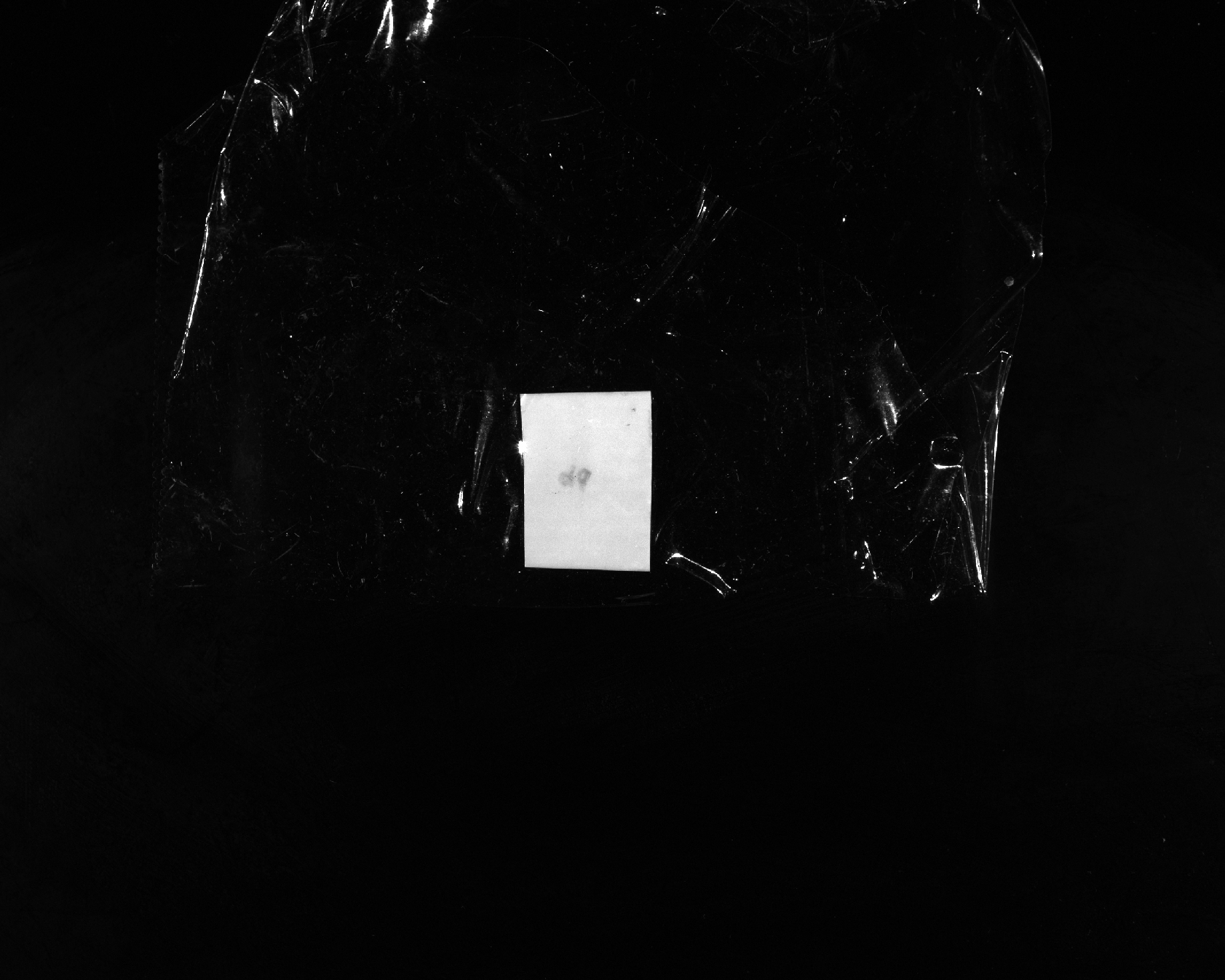

Supplement: Supplementary file 7 — Figure EV4 Source Data [file 44319_2026_744_MOESM7_ESM.zip › Figure 4/4A/day9/2025-01-09 setd2 d9(Ponceau S).jpg]

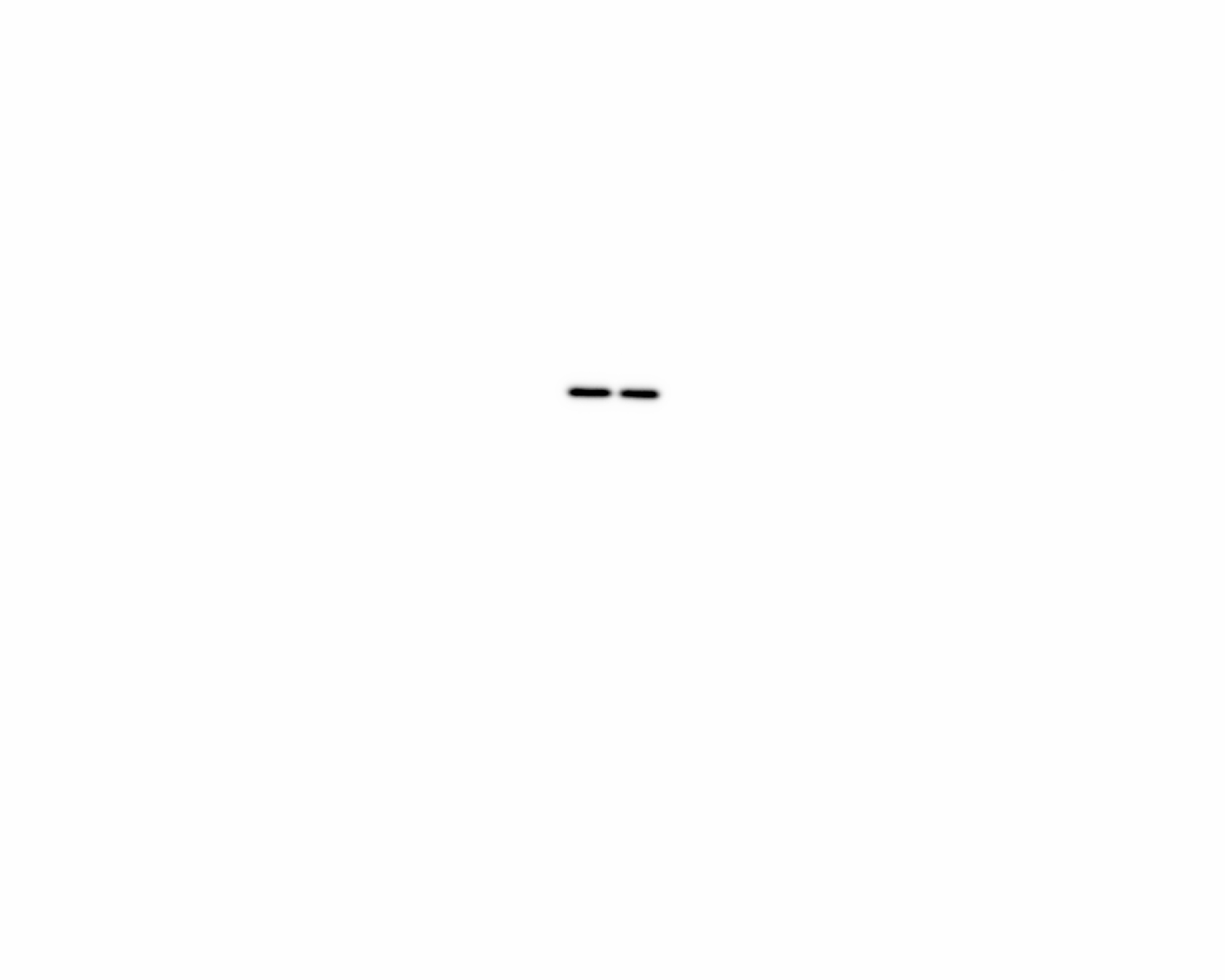

Supplement: Supplementary file 7 — Figure EV4 Source Data [file 44319_2026_744_MOESM7_ESM.zip › Figure 4/4A/day9/2025-01-21 h3 d9(Chemiluminescence).jpg]

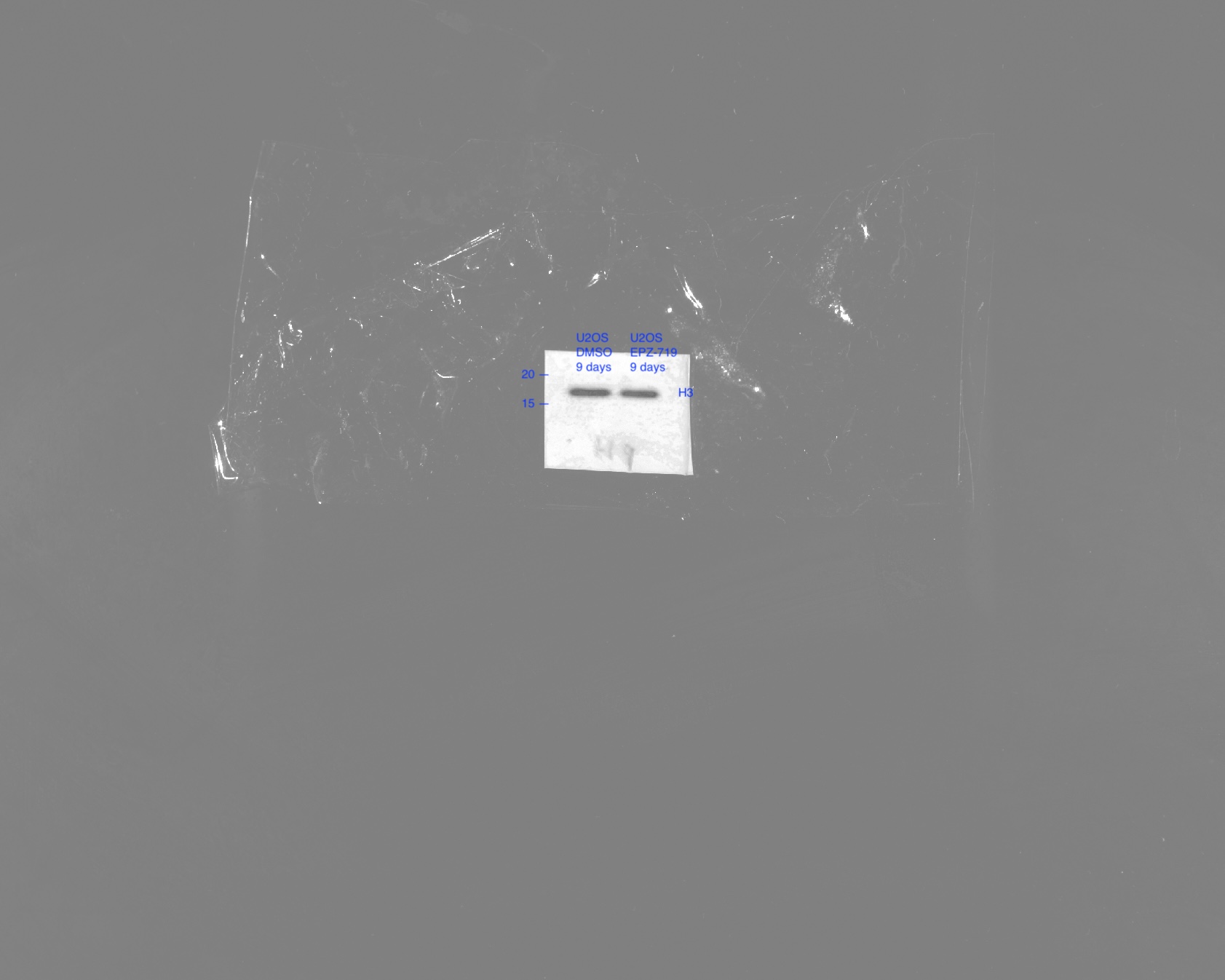

Supplement: Supplementary file 7 — Figure EV4 Source Data [file 44319_2026_744_MOESM7_ESM.zip › Figure 4/4A/day9/2025-01-21 h3 d9(Composite).jpg]

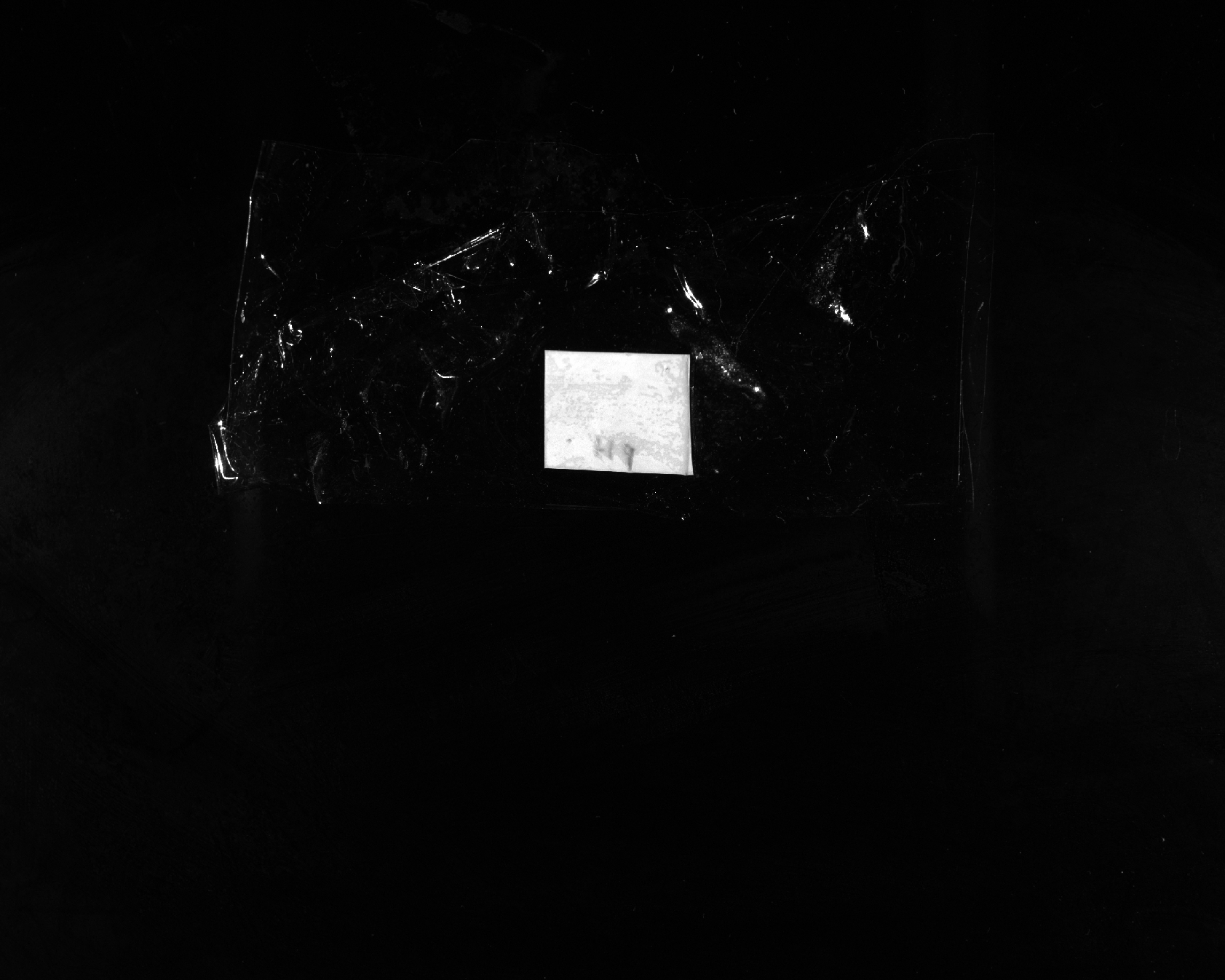

Supplement: Supplementary file 7 — Figure EV4 Source Data [file 44319_2026_744_MOESM7_ESM.zip › Figure 4/4A/day9/2025-01-21 h3 d9(Ponceau S).jpg]
