# Supplementary material for: SETD2 methyltransferase activity promotes correct transcription initiation and termination
Source: EMBO Rep. 2026 Mar 23;27(9):2218–42. doi: 10.1038/s44319-026-00744-1 (PMC13172057; doi:10.1038/s44319-026-00744-1)
Supplement: Supplementary file 8 — Expanded View Figures [file 44319_2026_744_MOESM8_ESM.pdf]

Expanded View Figures

Figure EV1. Characteristic features of class I and II genes.

(**A**) Biological replicates of POINT-seq and T4ph mNET-seq on *VASP* gene in U2OS. (**B**) Principal Component Analysis (PCA) of POINT-seq replicates in U2OS cells. (**C**) Illustration of class II gene definition: POINT-seq enrichment windows were identified and genes were classified as class II when the enrichment window for SETD2 KO or mutation was extended at least 1 kb in comparison to WT. (**D**) PCA for T4ph mNET-seq replicates in U2OS. (**E**) H3K36me3 signal on class I and class II genes in WT condition in U2OS (Wen et al, 2014; data ref: Wen et al, 2014). (**F**) H3K4me3 signal on class I and class II genes (Rane et al, 2024; data ref: Rane et al, 2024). (**G**) H3K27ac signal on class I and class II genes (Wu et al, 2024; data ref: Wu et al, 2024). (**H**) H4K16ac signal on class I and class II genes (Radzisheuskaya et al, 2021; data ref: Radzisheuskaya et al, 2021). (**I**) H3K27me3 signal on class I and class II genes (Jawhar et al, 2025; data ref: Jawhar et al, 2025). (**J**) H3K9me3 signal on class I and class II genes (data ref: Arroyo-Gómez and Reverón-Gómez, 2025). (**K**) Expression comparison between class I and class II genes in U2OS WT based on POINT-seq. (**L**) RNAPII elongation rates measured by 4sU-seq after (4, 8 and 16 min after DRB release), *n* class I = 257, *n* class II = 179 (Baluapuri et al, 2019; data ref: Baluapuri et al, 2019). (**E–L**) All dataset analyzed were derived from U2OS cells. (**M**) Gene length comparison between class I and class II genes in U2OS. (**N**) Intron length comparison (first, inner and last) between class I and class II genes in U2OS. (**O**) Copy number alterations in *SETD2* gene in RCC (blue) and ccRCC (gray). Data from TCGA accessed from https://www.cbioportal.org. (**P**) Expression of *SETD2* in ccRCC in normal (blue) and primary tumor (red), data from TCGA accessed from UALCAN (Chandrashekar et al, 2017). (**Q**) PCA for POINT-seq replicates in RCC. (**R**) POINT-seq replicates on *HK2* gene in RCC. (**S**) Overlap of class II genes between U2OS and RCC models. (**T**) Expression comparison between class I and class II genes in RCC SETD2 WT based on POINT-seq. (**U**) Gene length comparison between class I and class II genes in RCC. (**V**) Intron length comparison (first, inner and last) between class I and class II genes in RCC. Data information: Boxplots on (**K**), (**L**), (**N**), (**P**), (**T**), (**V**) show the median (center line), first and third quartiles (lower and upper bounds of the box), and whiskers extending to the most extreme data points within 1.5×IQR of the lower and upper quartiles, Mann–Whitney test was applied, $^{**}p \leq 0.01$, $^{****}p \leq 0.0001$. On (**F–K**), (**M**), (**N**) *n* class I = 4370, *n* class II = 1399. On (**T**), (**U**), (**V**) *n* class I = 4892, *n* class II = 862. The results on (**O**) and (**P**) are based upon data generated by the TCGA Research Network: https://www.cancer.gov/tcga.

© The Author(s)

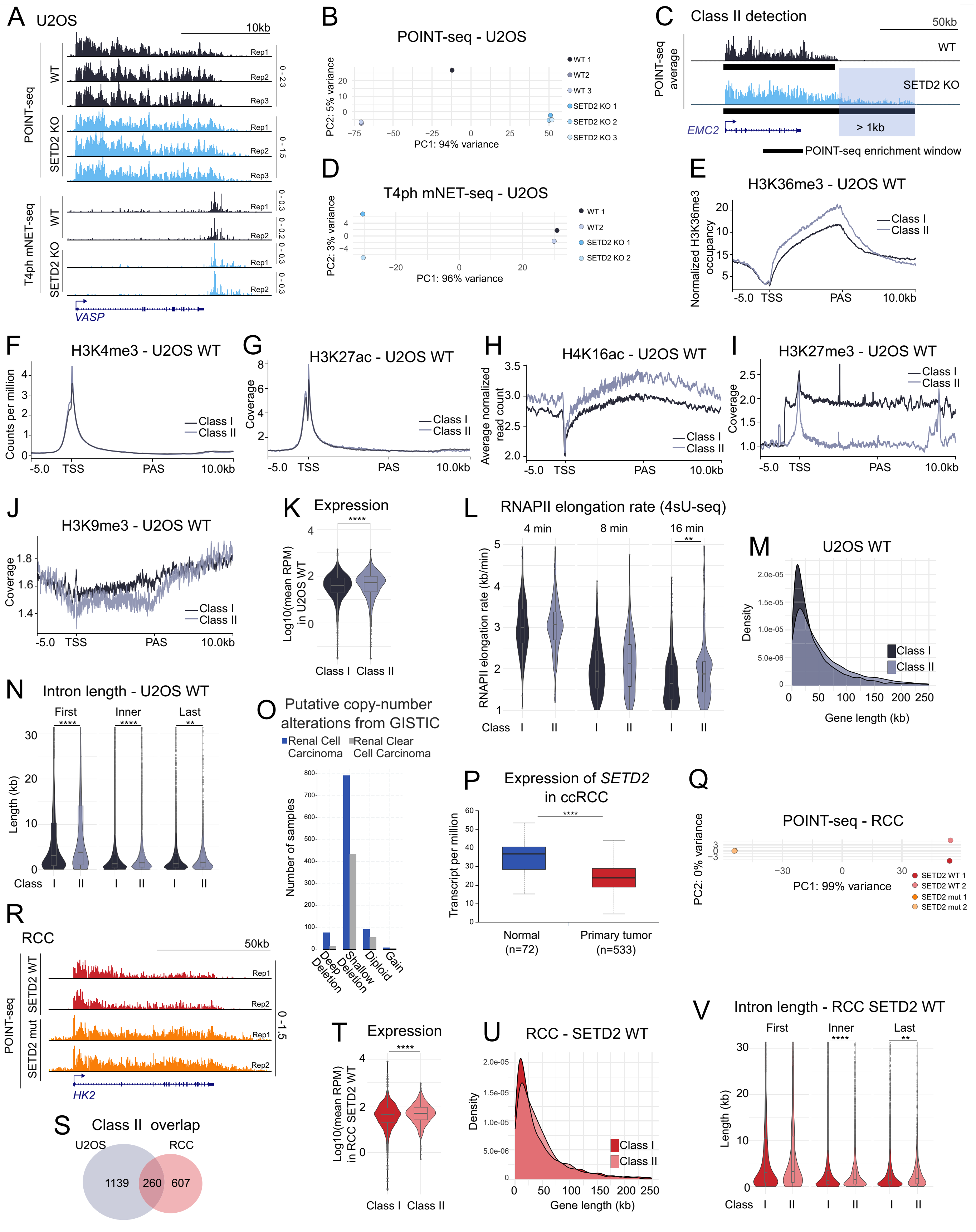
A U2OS
10kb
POINT-seq
WT
SETD2 KO
T4ph mNET-seq
Rep1
Rep2
Rep3
VASP
B POINT-seq - U2OS
PC1: 94% variance
PC2: 5% variance
WT 1
WT2
WT 3
SETD2 KO 1
SETD2 KO 2
SETD2 KO 3
C Class II detection
50kb
POINT-seq average
WT
SETD2 KO
EMC2
> 1kb
POINT-seq enrichment window
D T4ph mNET-seq - U2OS
PC1: 96% variance
PC2: 3% variance
E H3K36me3 - U2OS WT
Normalized H3K36me3 occupancy
Class I
Class II
-5.0 TSS PAS 10.0kb
F H3K4me3 - U2OS WT
Counts per million
G H3K27ac - U2OS WT
Coverage
H H4K16ac - U2OS WT
Average normalized read count
I H3K27me3 - U2OS WT
J H3K9me3 - U2OS WT
K Expression
Log10(mean RPM) in U2OS WT
Class I Class II
L RNAPII elongation rate (4sU-seq)
4 min
8 min
16 min
RNAPII elongation rate (kb/min)
M U2OS WT
Density
Gene length (kb)
N Intron length - U2OS WT
First
Inner
Last
Length (kb)
O Putative copy-number alterations from GISTIC
Renal Cell Carcinoma
Renal Clear Cell Carcinoma
Number of samples
Deep Deletion
Shallow Deletion
Diploid
Gain
P Expression of SETD2 in ccRCC
Transcript per million
Normal (n=72)
Primary tumor (n=533)
Q POINT-seq - RCC
PC1: 99% variance
PC2: 0% variance
SETD2 WT 1
SETD2 WT 2
SETD2 mut 1
SETD2 mut 2
R RCC
50kb
POINT-seq
SETD2 WT
SETD2 mut
HK2
S Class II overlap
U2OS
RCC
1139
260
607
T Expression
Log10(mean RPM) in RCC SETD2 WT
U RCC - SETD2 WT
V Intron length - RCC SETD2 WT

 © The Author(s)

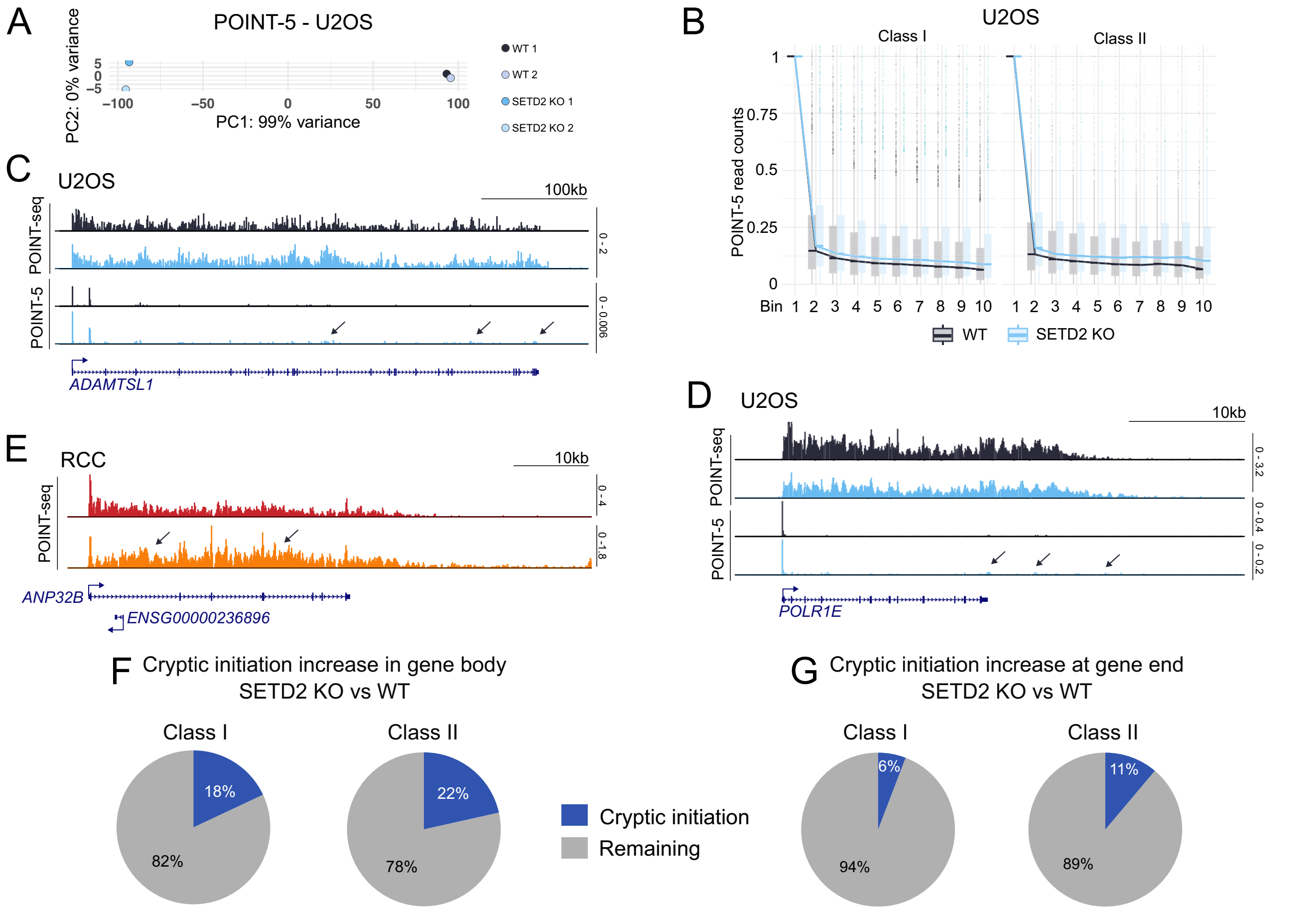

Figure EV2. Cryptic initiation upon SETD2 loss happens more frequently in class II genes.

(**A**) PCA for POINT-5 biological replicates in U2OS cells. (**B**) Boxplot representing the number of POINT-5 read counts calculated as described in Fig. 2D for class I, $n = 4229$ (left) and class II, $n = 1359$ (right) genes in U2OS WT (black) and SETD2 KO (blue). (**C**, **D**) Examples of genes with cryptic initiation in the gene body (indicated by arrows) in U2OS. (**E**) Example of a gene with cryptic initiation in the gene body (indicated by arrows) in RCC. (**F**, **G**) Quantification of genes which show cryptic initiation increase upon SETD2 KO, either in the entire gene body (bins 2–10, **F**), or specifically at the gene end (bins 9 and 10, **G**). For each gene, its gene body signal was normalized to TSS-proximal signal (bin 1) to account for changes in normal transcriptional activity following KO (see also Methods). Data information: Boxplot on (**B**) shows the median (center line), first and third quartiles (lower and upper bounds of the box), and whiskers extending to the most extreme data points within 1.5×IQR of the lower and upper quartiles.

© The Author(s)

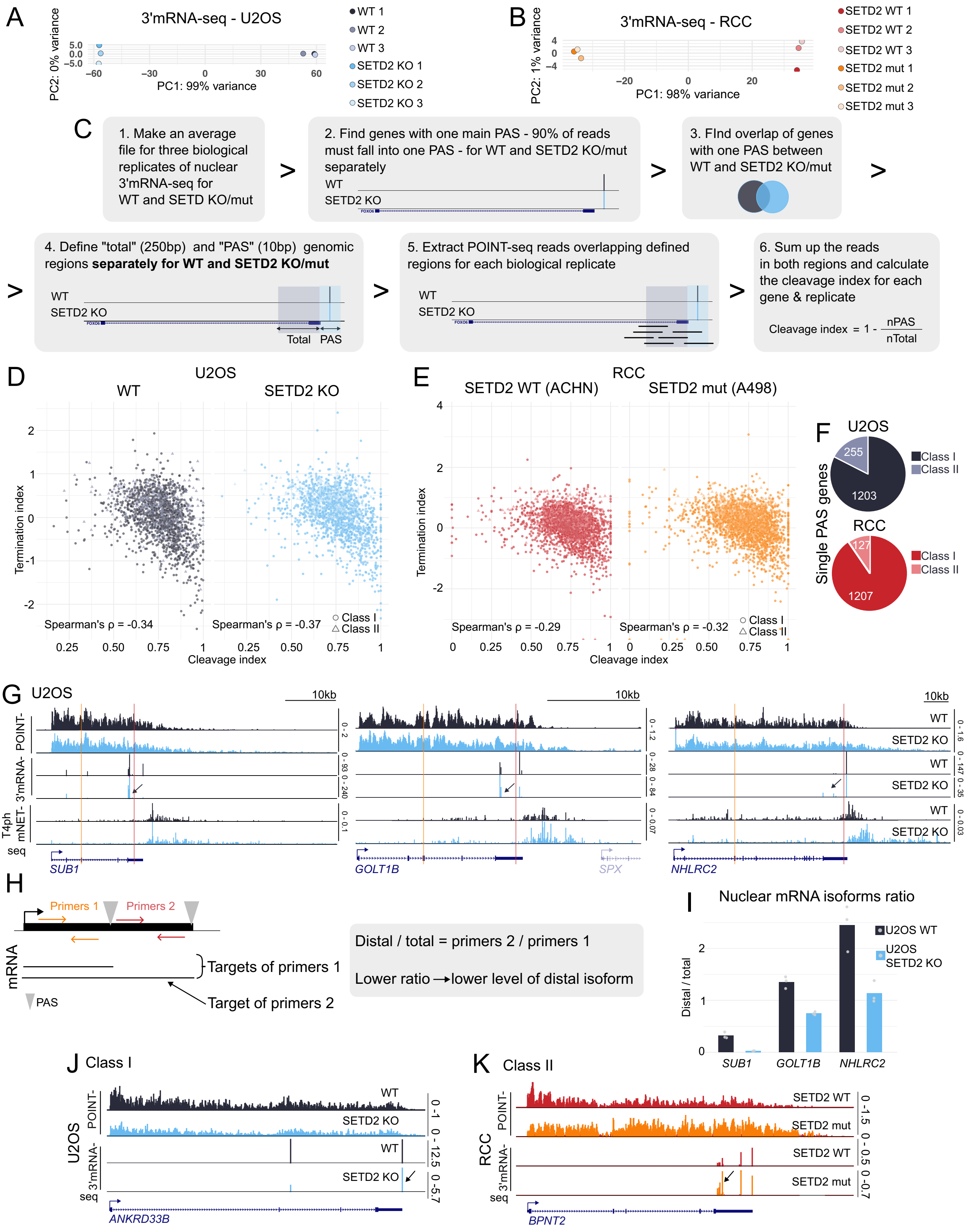
A
3'mRNA-seq - U2OS
PC2: 0% variance
PC1: 99% variance
WT 1
WT 2
WT 3
SETD2 KO 1
SETD2 KO 2
SETD2 KO 3
B
3'mRNA-seq - RCC
PC2: 1% variance
PC1: 98% variance
SETD2 WT 1
SETD2 WT 2
SETD2 WT 3
SETD2 mut 1
SETD2 mut 2
SETD2 mut 3
C
1. Make an average file for three biological replicates of nuclear 3'mRNA-seq for WT and SETD KO/mut
2. Find genes with one main PAS - 90% of reads must fall into one PAS - for WT and SETD2 KO/mut separately
3. FInd overlap of genes with one PAS between WT and SETD2 KO/mut
4. Define "total" (250bp) and "PAS" (10bp) genomic regions separately for WT and SETD2 KO/mut
Total
PAS
5. Extract POINT-seq reads overlapping defined regions for each biological replicate
6. Sum up the reads in both regions and calculate the cleavage index for each gene & replicate
Cleavage index = 1 - nPAS / nTotal
D
U2OS
WT
SETD2 KO
Termination index
Spearman's ρ = -0.34
Spearman's ρ = -0.37
Class I
Class II
Cleavage index
E
RCC
SETD2 WT (ACHN)
SETD2 mut (A498)
Termination index
Spearman's ρ = -0.29
Spearman's ρ = -0.32
Class I
Class II
Cleavage index
F
U2OS
255
1203
Class I
Class II
Single PAS genes
RCC
127
1207
Class I
Class II
G
U2OS
10kb
POINT-
3'mRNA-
T4ph mNET-
seq
WT
SETD2 KO
SUB1
GOLT1B
SPX
NHLRC2
H
Primers 1
Primers 2
mRNA
Targets of primers 1
Target of primers 2
PAS
Distal / total = primers 2 / primers 1
Lower ratio → lower level of distal isoform
I
Nuclear mRNA isoforms ratio
Distal / total
U2OS WT
U2OS SETD2 KO
SUB1
GOLT1B
NHLRC2
J
Class I
U2OS
POINT-
3'mRNA-
seq
WT
SETD2 KO
ANKRD33B
K
Class II
RCC
POINT-
3'mRNA-
seq
SETD2 WT
SETD2 mut
BPNT2

© The Author(s)

Figure EV3. Cleavage index calculation and APA validation.

(**A**, **B**) PCA for 3′mRNA-seq biological replicates in U2OS cells (**A**) and RCC cells (**B**) either WT or SETD2 KO/mut. (**C**) Schematic representation of the cleavage index calculation methodology. (**D**) Scatterplot representing correlation between termination index and cleavage index in U2OS WT (black) vs SETD2 KO (blue), $n = 1769$. (**E**) Scatterplot representing correlation between termination index and cleavage index in RCC SETD2 WT (red) vs SETD2 mutation (orange), $n = 2322$. (**F**) Pie charts of class I and class II gene numbers in single PAS gene category in U2OS (top) and RCC (bottom) models. (**G**) Genome browser snapshots of genes used for APA validation: *SUB1*, *GOLT1B* and *NHLRC2*. Orange and red highlights represent the regions targeted by the primers. (**H**) Schematic representation of qPCR assay used for calculating distal/total isoforms ratio. Primers were designed to target all isoforms (primers 1) and the most distal isoform (primers 2). Distal to total ratio was calculated by dividing the amount of product targeted by primers 2 by product of primers 1. (**I**) Distal/total ratio of nuclear mRNA isoforms for *SUB1*, *GOLT1B* and *NHLRC2* genes. Each biological replicate is represented by a dot ($n = 3$). (**J**) Gene example of class I gene with distal APA (indicated by an arrow) in U2OS. (**K**) Gene example of class II gene with proximal APA (indicated by an arrow) in RCC.

© The Author(s)

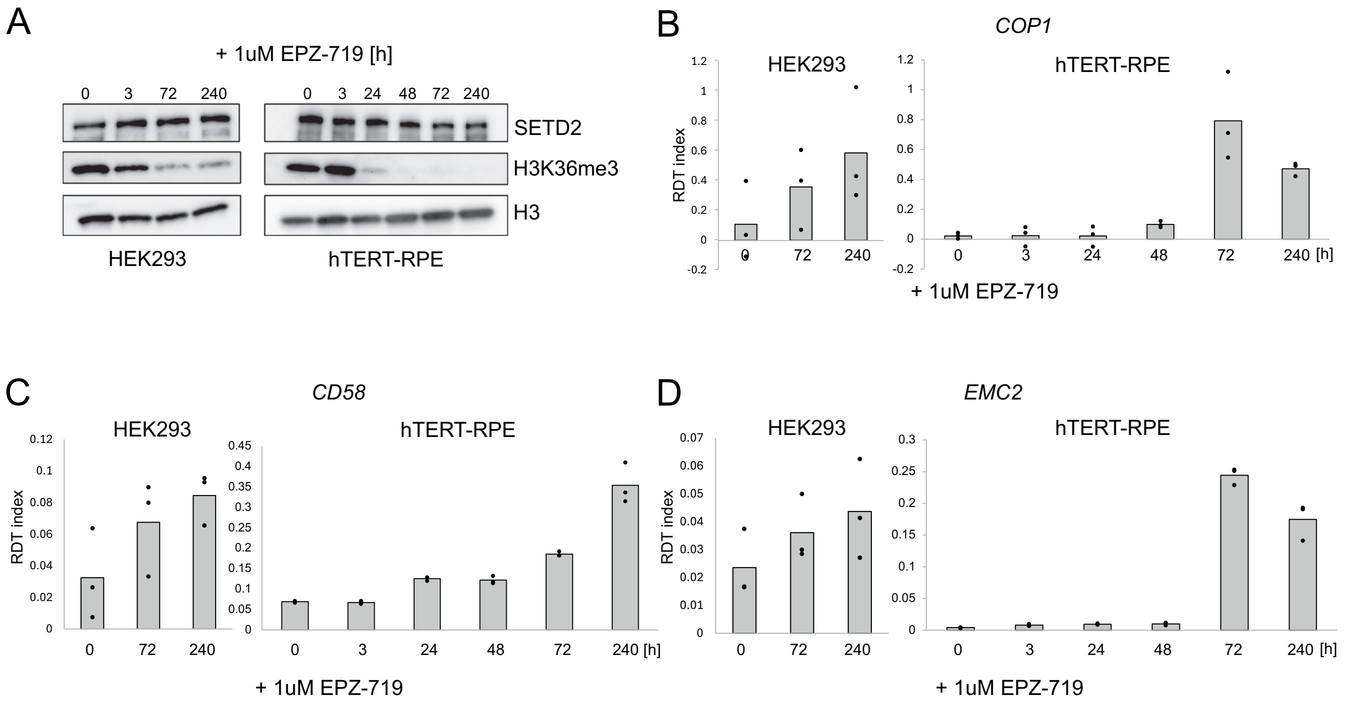

Figure EV4. SETD2 inhibition leads to transcription readthrough induction also in non-cancerous cells.

(**A**) Western Blot of SETD2, H3K36me3 and H3 in HEK293 (left) and hTERT-RPE cells (right) treated with the SETD2 inhibitor EPZ-719 for the indicated number of hours. (**B–D**) RDT index calculated for *COP1*, *CD58* and *EMC2* genes in HEK293 (left) and hTERT-RPE (right) cells treated with EPZ-719 for the indicated number of hours. Individual dots represent distinct biological replicates ($n = 3$). Source data are available online for this figure.

 © The Author(s)

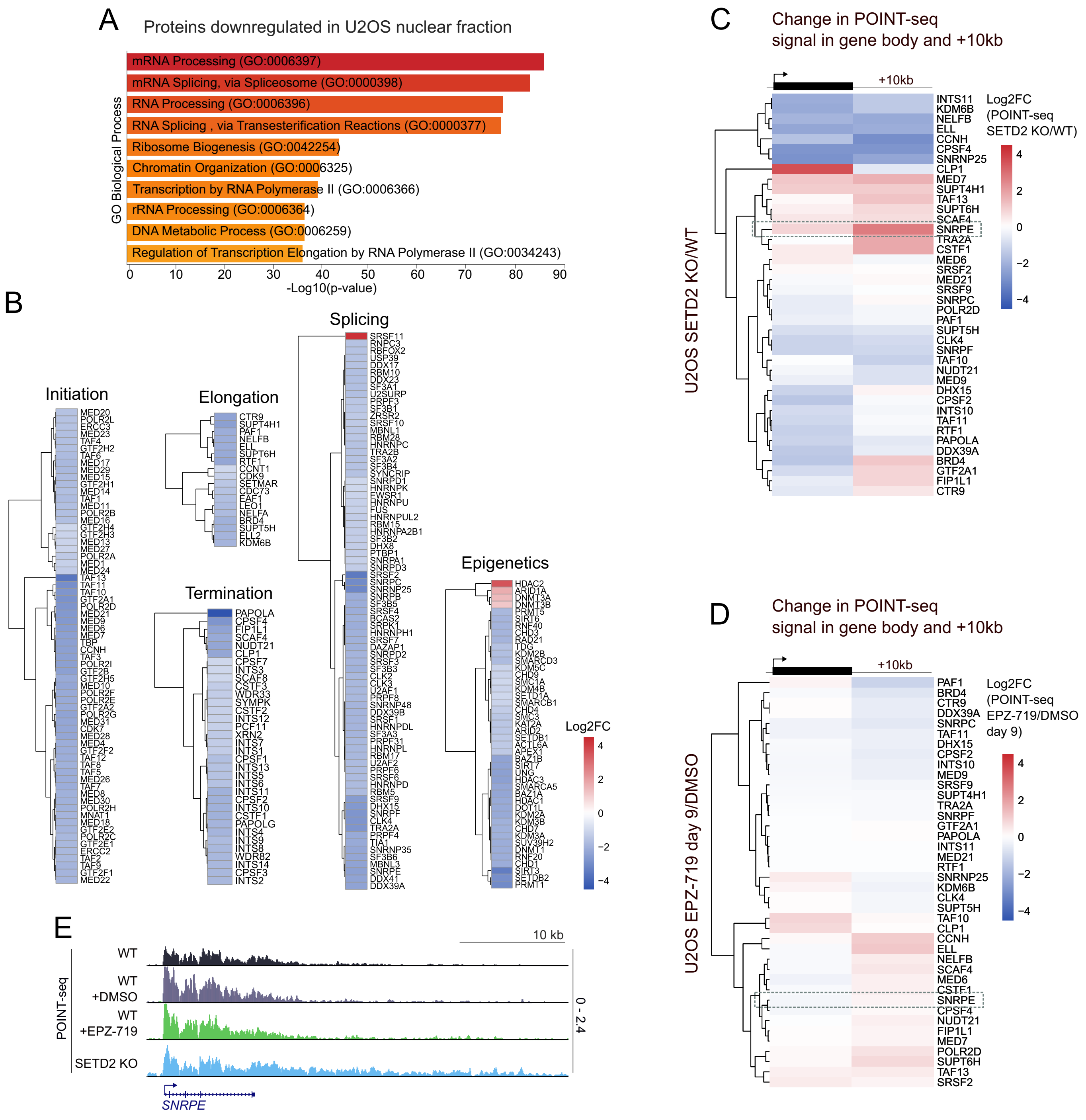

Figure EV5. SETD2 loss leads to downregulation of key transcription-related proteins in the nuclear fraction.

(**A**) Gene ontology results representing biological process that proteins downregulated in U2OS nuclear fraction are involved in. (**B**) Heatmaps representing log2fc of protein abundance in SETD2 KO vs WT in nuclear fraction of U2OS cells. Proteins were divided into groups corresponding to their involvement in different stages or transcriptional control. (**C**) Heatmap representing change in POINT-seq signal in gene body and 10 kb downstream of the annotated gene end in U2OS SETD2 KO/WT. (**D**) Heatmap representing change in POINT-seq signal in gene body and 10 kb downstream of the annotated gene end in U2OS WT + DMSO/WT + EPZ-710. (**E**) Gene example of POINT-seq signal (marked on (**C**) and (**D**) by a dotted rectangle) in U2OS WT (black), WT + DMSO (gray), WT + EPZ-719 (green) and SETD2 KO (blue). Data information: for (**A**) Fisher's exact test was applied.

© The Author(s)
